# QTL associated with resistance to cassava brown streak and cassava mosaic diseases in a bi-parental cross of two Tanzanian farmer varieties, Namikonga and Albert

**DOI:** 10.1007/s00122-017-2943-z

**Published:** 2017-07-13

**Authors:** E. A. Masumba, F. Kapinga, G. Mkamilo, K. Salum, H. Kulembeka, S. Rounsley, J. V. Bredeson, J. B. Lyons, D. S. Rokhsar, E. Kanju, M. S. Katari, A. A. Myburg, N. A. van der Merwe, M. E. Ferguson

**Affiliations:** 1 Sugarcane Research Institute, P. O. Box 30031, Kibaha, Tanzania; 2 Naliendele Agricultural Research Institute, P. O. Box 509, Mtwara, Tanzania; 3 Ukiriguru Agricultural Research Institute, P. O. Box 1433, Mwanza, Tanzania; 4 Genus plc, DeForest, WI USA; 5 Molecular and Cell Biology Department, University of California, Berkeley, CA USA; 6 International Institute of Tropical Agriculture (IITA), P.O. Box 2066, Dar es Salaam, Tanzania; 7 New York University, New York, USA; 8 Department of Genetics, Forestry and Agricultural Biotechnology Institute (FABI), University of Pretoria, Private Bag X20, Hatfield, 0028 South Africa; 9 IITA, P.O. Box 30709-00100, Nairobi, Kenya

## Abstract

*****Key message***:**

**QTL consistent across seasons were detected for resistance to cassava brown streak disease induced root necrosis and foliar symptoms. The CMD2 locus was detected in an East African landrace, and comprised two QTL**.

**Abstract:**

Cassava production in Africa is compromised by cassava brown streak disease (CBSD) and cassava mosaic disease (CMD). To reduce costs and increase the precision of resistance breeding, a QTL study was conducted to identify molecular markers linked to resistance against these diseases. A bi-parental F_1_ mapping population was developed from a cross between the Tanzanian farmer varieties, Namikonga and Albert. A one-step genetic linkage map comprising 943 SNP markers and 18 linkage groups spanning 1776.2 cM was generated. Phenotypic data from 240 F_1_ progeny were obtained from two disease hotspots in Tanzania, over two successive seasons, 2013 and 2014. Two consistent QTLs linked to resistance to CBSD-induced root necrosis were identified in Namikonga on chromosomes II (qCBSDRNFc2Nm) and XI (qCBSDRNc11Nm) and a putative QTL on chromosome XVIII (qCBSDRNc18Nm). qCBSDRNFc2Nm was identified at Naliendele in both seasons. The same QTL was also associated with CBSD foliar resistance. qCBSDRNc11Nm was identified at Chambezi in both seasons, and was characterized by three peaks, spanning a distance of 253 kb. Twenty-seven genes were identified within this region including two LRR proteins and a signal recognition particle. In addition, two highly significant CMD resistance QTL (qCMDc12.1A and qCMDc12.2A) were detected in Albert, on chromosome 12. Both qCMDc12.1A and qCMDc12.2A lay within the range of markers reported earlier, defining the CMD2 locus. This is the first time that two loci have been identified within the CMD2 QTL, and in germplasm of apparent East African origin. Additional QTLs with minor effects on CBSD and CMD resistance were also identified.

**Electronic supplementary material:**

The online version of this article (doi:10.1007/s00122-017-2943-z) contains supplementary material, which is available to authorized users.

## Introduction

Cassava (*Manihot esculenta* Crantz) (2*n* = 36), a starchy root crop, is widely grown in many parts of sub-Saharan Africa, with the major producers being Nigeria, Democratic Republic of Congo, Angola, Ghana, Mozambique, Uganda, and Tanzania (FAOSTAT 2015). In these regions, cassava provides the primary food source for millions of people (FAO 2010) and is strategically grown for food security and income generation (El-Sharkawy 2004; Legg et al. 2014). Cassava has the largest productivity per unit area of any crop grown in Africa (FAOSTAT 2017) and accounts for over 55% of the total world production (FAOSTAT 2015; Legg et al. 2014). The crop is consumed boiled, or dried and pounded into a flour which is known as ‘gari’ in Nigeria. It is also used as an industrial raw material for starch and bio-ethanol as well as for animal feed (Balagopalan 2002; Ceballos et al. 2010; Maziya-Dixon et al. 2006).

Tanzania is the second largest producer of cassava in East Africa after Uganda (FAOSTAT 2015) with average yields of 5.5 tha^−1^ (FAOSTAT 2015). This is far below the estimated yield potential of cassava in East Africa (50–60 tha^−1^) (Fermont et al. 2009), and represents a dramatic yield decline from 1996, in which 10.5 tha^−1^ was reported (FAOSTAT 2015). Among other biotic and abiotic factors such as increased whitefly and cassava green mite populations (Campo et al. 2011) and low external inputs (Howeler et al. 2013), cassava mosaic (CMD) and cassava brown streak (CBSD) diseases are major contributors to this decline (Hillocks et al. 2001; Legg et al. 2007). Both diseases were first recognized in northeastern coastal areas of Tanzania, CMD in 1894 (Thresh 2003) and CBSD in the early 1930s (Storey 1936). CMD is widely distributed across the African continent and Indian sub-continent (Alabi et al. 2011; Hillocks 1997), whereas CBSD was initially restricted to low-altitude areas of East Africa along the Indian Ocean (Hillocks and Jennings 2003; Jennings 2003), but later reported from high-altitude areas (>1000 meters above sea level) (Ntawuruhunga and Legg 2007). These areas include those surrounding Lake Victoria in northwestern Tanzania, western Kenya and central Uganda (Alicai et al. 2007; Ntawuruhunga and Legg 2007), Burundi (Bigirimana et al. 2011), and some areas of the Democratic Republic of Congo (Mulimbi et al. 2012). Further spread of CBSD towards West Africa, the largest cassava-producing region in Africa, is projected (Legg et al. 2014; Patil et al. 2014).

CBSD and CMD are caused by different groups of viruses, but are transmitted by a common vector, *Bemisia tabaci* (whitefly) (Maruthi et al. 2005). Two virus species have been reported to cause CBSD, namely cassava brown streak virus (CBSV) and Ugandan cassava brown streak virus (UCBSV), both belonging to the genus *Ipomovirus* (family *Potyviridae*) (Mbanzibwa et al. 2009, 2011; Winter et al. 2010). In this publication, we use CBSVs to imply both CBSV and UCBSV. Since these viruses have only recently been recognized, descriptions of further CBSD-causing viruses can be expected (Ndunguru et al. 2015).

Symptoms of CBSD occur throughout the plant, on leaves, stems and roots (Hillocks and Thresh 2000). It is manifested on leaves by various leaf chlorosis patterns, starting from the leaf veins towards the entire leaf surface (Hillocks and Jennings 2003). Symptoms on stems lead to the occurrence of purplish brown lesions (Hillocks and Thresh 2000). In severe cases, the stem symptoms cause death of the axillary buds (Hillocks and Jennings 2003) leading to a condition known as ‘dieback’ (Hillocks and Thresh 2000). Root symptoms are the most economically damaging, appearing as yellow to brown corky necrotic patches in the storage roots rendering them inedible (Hillocks and Thresh 2000; Ntawuruhunga and Legg 2007).

Efforts to control CBSD and CMD were initiated in the early 1930s at the East African Cassava Research Institute at Amani in northeastern Tanzania (Jennings 1976, 2003; Nichols 1950). Due to a lack of resistance in cassava, breeders resorted to introgression of disease resistance through interspecific crosses with wild *Manihot* species (Nichols 1950). The breeding work successfully developed several hybrids including 46106/27, which showed high levels of field resistance to CBSD (Hillocks and Jennings 2003; Jennings 2003). Many of these hybrids dissipated into local farming systems. It has been shown that hybrid 46106/27, known as Amani in Tanzania, is closely related to, but not identical to, a Tanzanian local cultivar Namikonga (Kulembeka 2010; Pariyo et al. 2013). Namikonga is, therefore, suspected to be an interspecific hybrid from the Amani program that was subsequently adopted by the farming communities and given a local name. At present, Namikonga still expresses field resistance to CBSD and is used as one of the best sources of CBSD resistance in conventional breeding programs (Jennings 2003; Kanju et al. 2010; Kaweesi et al. 2014; Maruthi et al. 2014; Pariyo et al. 2013; Rwegasira and Rey 2012). The variety is grown by farmers to a limited extent in southeastern Tanzania, although the yield is low. More recently, breeders have been exploiting other natural sources of CBSD resistance (Kawuki et al. 2016); however, immunity to virus infection has so far been elusive. Genetic engineering has generated immunity to CBSVs in the model cassava cultivar 60444 (Vanderschuren et al. 2012).

Analysis of whole genome shotgun sequencing by Bredeson et al. (2016) revealed a parent–offspring relationship of Namikonga with a Nigerian landrace TME117. Namikonga shares an entire haplotype with TME117. One explanation for this is that, prior to interspecific hybridization, many *M. esculenta* varieties from different cassava-growing regions of the world, including West Africa, were evaluated for virus resistance at Amani (Nichols 1947). It is suspected that TME117 was amongst these varieties as it was presumably used as a parent in the Amani breeding program (Jennings 2015). Evidence that Namikonga was derived from the Amani breeding program comes from the fact that 14.4% of the Namikonga genome was of the *M. glaziovii*–*M. esculenta* hybrid type, and it contains an indicative introgression segment on chromosome 1 (Bredeson et al. 2016). A diallel analysis conducted by Kulembeka et al. (2012) found that CBSD resistance in Namikonga was due to two or more genes with additive effects.

Albert, another Tanzanian local cultivar and putative full-sib of TME117 (Bredeson et al. 2016), is extremely susceptible to CBSD, although it shows high levels of field resistance to CMD (Maruthi et al. 2014; Mtunda et al. 2003; Rwegasira and Rey 2012). Two known sources of CMD resistance are recognized, one largely influenced by a genomic region known as CMD2 discovered in a Nigerian landrace TME3 (Akano et al. 2002; Rabbi et al. 2014), and a more quantitative source of CMD resistance called CMD1, derived from an Amani interspecific cross, now known as TMS 30572 (now TMS-I30572) (Fregene et al. 2000; Mohan et al. 2013). A third putative source of resistance, known as CMD3, has also been described (Okogbenin et al. 2012). Novel sources and additional information on the genetic basis of CBSD and CMD resistance is urgently needed by breeding programs in Africa.

Most cassava breeding programs in Africa use purely conventional breeding methods that are hindered by long breeding cycles, genotype x environment interactions and large, expensive field trials (Ceballos et al. 2004, 2015). The use of molecular markers in breeding for disease resistance has yielded successful results in wheat (Kuchel et al. 2007), regardless of logistical challenges (Heffner et al. 2009; Xu and Crouch 2008). Selection based on molecular markers that define a quantitative trait locus (QTL) can effectively increase the heritability of the associated trait by negating environmental influence. Marker-assisted selection (MAS) enables selection of progeny at the seedling stage, meaning that only individuals with the preferred allelic composition are planted for further evaluation. Through increases in heritability, it is likely that a reduced number of breeding cycles may be necessary in the varietal development process.

In cassava, MAS has not been widely adopted but has been used to a limited extent to introgress the CMD2 locus into Latin American germplasm using Simple Sequence Repeat (SSR) and Sequence Characterized Amplified Regions (SCAR) markers (Ceballos et al. 2015; Egesi et al. 2006; Okogbenin et al. 2007). Additionally, the identification of molecular markers associated with CBSD resistance would enable pre-emptive breeding through MAS for CBSD resistance in those countries not yet affected, but threatened, by the disease. The current study aimed at the identification of QTL associated with CBSD and CMD resistance in the Tanzanian landraces Namikonga and Albert, and the characterization of QTL genomic regions associated with CBSD resistance in Namikonga.

## Materials and methods

Varieties Namikonga and Albert differ in terms of their response towards CBSVs infection (Kaweesi et al. 2014; Maruthi et al. 2014; Rwegasira and Rey 2012). A filial 1 (F_1_) mapping population was developed from a cross between Namikonga, a CBSD-tolerant but CMD-susceptible variety, and Albert which contrastingly is CBSD susceptible and CMD resistant. Namikonga was used as the female parent and Albert, a prolific pollen producer, as the male parent. Stakes of parental genotypes were collected from farmers’ fields and research stations and planted in two crossing blocks at Kibaha and Naliendele research centers in eastern and southern Tanzania, respectively. The two sites were selected based on the adaptation of the parental genotypes to the hot, humid conditions of coastal Southeast Tanzania (Kanju et al. 2010). To facilitate movement during pollination, stakes were planted at a spacing of 1 m by 2 meters as intra-row and inter-row spacing, respectively. Pollinations were performed by hand. Each morning before pollination, transparent mesh bags were used to cover mature female flowers on Namikonga to avoid pollen contamination. Pollen was collected from the male parent Albert and stored in a perforated and well-aerated paper container. Pollinations were performed from around midday by uncovering the mature female flowers and dusting the stigma with the collected pollen. All non-mature flowers were detached from the inflorescence immediately after pollination and the pollinated inflorescence was labeled with the date of crossing, names of the parents and the number of pollinated flowers. About four weeks after pollination the mature fruits were bagged in well-aerated seed collection bags as fruits undergo biocidal dehiscence (Chavarriaga-Aguirre and Halsey 2005). Mature seeds were harvested from 75 to 90 days after pollination (Alves 2002) and stored for a dormancy period of about 2 to 3 months prior to germination. Seeds were germinated in seed trays in a clean disinfected screen house and seedlings transplanted into a CBSD and CMD free site at Makutupora research station (5.97°S:3, 5.76°E) in central Tanzania for production of planting stakes. At three months after planting (MAP), leaf samples were collected from 569 vigorous individuals for DNA extraction and subsequent analysis.

### Validation of true crosses using simple sequence repeat markers

Due to the outcrossing nature of cassava, and the fact that parental stakes were partly derived from farmers’ fields, the integrity of the putative mapping population was assessed for ‘off-types’ and ‘selfs’ (which are synonymous with outcross progeny derived from two identical genotypes of this clonally propagated crop) using SSR fingerprinting. Genomic DNA was extracted using a Miniprep extraction protocol, a modification of Dellaporta et al. (1983). DNA quantity and quality was assessed using NanoDrop (NanoDrop^®^ ND-1000 Spectrophotometer) and confirmed using 1% agarose gel electrophoresis (Treseder-laboratory 2007) stained with GelRed^TM^ nucleic acid gel stain (Biotium) (Warren 2007). Reaction conditions were according to Kawuki et al. (2013). SSR markers that were polymorphic between the two parents were identified and 15 primer pairs which produced unambiguous amplification products (SSRY9, 19, 27, 44, 55, 165, 185, 229, 253, 282, 32, 122, 151, NS160 and NS909) (Mba et al. 2001) were used to select F_1_ true-cross progeny. Amplification products were resolved using capillary electrophoresis on an ABI 3730 and scored using GeneMapper v4.1 software.

### Genotyping-by-sequencing (GBS) library preparation, sequencing and variant calling

Genotyping of the population was performed using a reduced representation approach, namely genotyping-by-sequencing (GBS) (Elshire et al. 2011) with modifications (International Cassava Genetic Map Consortium 2015) at the University of California, Berkeley. Due to the presence of highly repetitive sequences in the cassava genome (Prochnik et al. 2012), sites with an average read depth exceeding 120 reads per individual were excluded. High-quality read data, free from adapter sequences, were trimmed using a custom BWA-like trimming script. Burrows Wheeler Aligner (BWA) (Li and Durbin 2009) suitable for short-read alignment was used to align individual genomes to the cassava reference assembly version 5.1. Single nucleotide polymorphic markers (SNPs) were extracted using the HaplotypeCaller tool from the Genome Analysis Toolkit (GATK) (v2.7-2) (International Cassava Genetic Map Consortium 2015). Stringent filtering of the variant sites was performed and variant sites with *P* < 0.05 segregation distortion were excluded. SNP markers were named according to the chromosome number (Roman numerals in v5.1 of the cassava genome assembly) and base pair (bp) position.

### Construction of genetic linkage maps

Genotyping data of the F_1_ progeny obtained from GBS were used for linkage mapping. Markers and individuals with more than 20% missing data were excluded from the analysis. SNP data were formatted according to the cross-pollinated (CP) option in JoinMap^®^ 4.1. (van Ooijen 2006), which is appropriate for outcrossing species in which both parents are heterozygous and the linkage phase is unknown. Bi-allelic and tri-allelic SNP data provided segregation types *lmxll*, *nnxnp, hkxhk*, and *efxfg*. Tetra-allelic SNPs were excluded from the analysis, together with markers with 99% or greater similarity. The remaining SNP data with less than 20% missing values were used to generate a one-step high-density genetic linkage map using JoinMap^®^ version 4.1 (van Ooijen 2006). Linkage groups were established using a minimum LOD of 5.0 per group and marker order was defined using the regression mapping algorithm (Wu et al. 2014) and Kosambi’s mapping function (van Ooijen 2009). The linkage groups were named according to the corresponding chromosome as defined by the International Cassava Genetic Map Consortium (2015). Using the high-density mapping results, a low-density, high-confidence framework map with markers approximately 5 centiMorgans (cM) apart was generated (Darvasi et al. 1993). The order of the markers for both the high-density and framework maps was consistent with that of version 5.1 of the cassava genome sequence (International Cassava Genetic Map Consortium 2015) [http://www.cassavabase.org/cview/map.pl?map_id=3] [http://portal.nersc.gov/dna/plant/cassava_wgs/assmV5.1/]. The framework map was initially used for QTL detection, but in an attempt to close a large inter-marker distance at a CBSD resistance QTL, and to obtain more resolution at a CMD locus, the high-density map was also used (Stange et al. 2013). As most of the markers are not completely informative, including all markers in a linkage group may improve the power and accuracy of estimates, especially in outbreeding populations (Knott et al. 1997).

### Phenotypic data analysis and detection of QTL associated with CBSD and CMD resistance

**Fig. 1:**
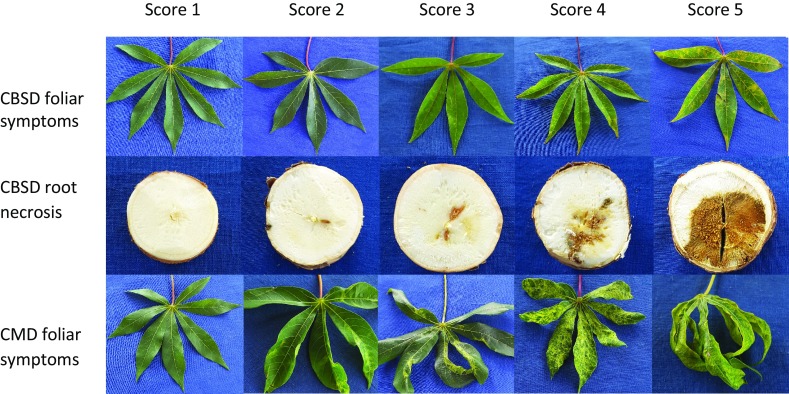
CBSD and CMD symptom scoring scale (1–5)

Cassava stakes (cuttings) were collected from F_1_ individuals previously maintained and bulked at Makutupora research center, a CBSD- and CMD-free site. Phenotyping trials were established in CMD and CBSD hot spot areas at Naliendele (10.38°S, 40.16°E) and Chambezi (6.55°S, 38.91°E) research centers in the southern and eastern coastal areas of Tanzania. Genotypes were evaluated in two consecutive seasons, namely 2013 and 2014. The site–season combinations were designated as experiments N1 and N2 for Naliendele in season 1 and 2, and experiments C1 and C2 for Chambezi for the same seasons. The number of genotypes to be evaluated per trial per season was determined by the number of individuals having sufficient stakes (cuttings) to establish a trial. Therefore, 223 genotypes were evaluated in 2013 and 280 genotypes in 2014. Due to the large number of individuals being evaluated, an alpha lattice experimental design with incomplete blocks was used (Kashif et al. 2011). Five plants per genotype were used per plot, in two or three replications, and planted at 1 m x 1 m spacing. To increase the disease pressure and the chance that all plants were equally exposed to the diseases, spreader rows with infected plants were planted adjacent to each row and surrounding the trial. Cuttings planted for spreaders were taken from plants that clearly showed CMD and CBSD symptoms. These were obtained from farmer fields close to the experimental sites. Separate phenotypic data for CMD and CBSD (both leaf and root) symptoms were scored on a scale of 1–5 (Fig. 1) (Hillocks and Thresh 2000; National Cassava Research 2006). Measurements for foliar symptoms of CBSD and CMD were taken at 3-month intervals at 3, 6 and 9 MAP, whereas CBSD root necrosis measurements were taken at harvest (12 MAP). Shapiro–Wilk normality (SWILK) (Shapiro and Wilk 1965), incorporated in the Genetic Analysis of Clonal F1 and Double Cross population (GACD v 1.1) mapping software (Zhang et al. 2015), was used to determine the normality of the trait frequency distributions across the locations in both seasons.

### Detection of CBSV and UCBSV in experimental sites

To ascertain the relative incidence of CBSV and UCBSV in the experiments, leaf samples were taken from 26 to 30 randomly selected genotypes from Chambezi and Naliendele, respectively, each genotype having three replications. The genotypes included individuals that scored class 1 (no symptom), class 3 (mild symptoms on leaves and stem), and class 5 (severely infected) on the CBSD severity scale. In each selected plant, the second fully expanded central leaf from the shoot apex was picked and press dried on herbarium newspaper (GLCI 2010). Total RNA was extracted using a pine tree RNA extraction method (Chang et al. 1993), with modifications adopted from Moreno et al. (2011). The quantity and quality of each RNA sample was assessed using NanoDrop (NanoDrop ^®^ ND-1000 Spectrophotometer). Real-time reverse transcription polymerase chain reaction (RT-PCR) assays based on Taqman chemistry was used to detect and distinguish the two virus species (CBSV and UCBSV) on a GeneAmp PCR System 9700 (Applied Biosystems) (Adams et al. 2013).

### QTL analysis

QTL analysis to identify genomic regions that associate with CBSD resistance in Namikonga based on both foliar and root symptoms, and CMD resistance in Albert was performed through interval mapping (IM) and inclusive composite interval mapping (ICIM) using low- and high-SNP density maps initially generated by JoinMap ^®^ 4.1. software (van Ooijen 2006). QTL mapping by IM was performed by MapQTL^®^ 6 QTL mapping software (van Ooijen 2009) and ICIM by GACD (v 1.1) (Zhang et al. 2015). ICIM using a high-density map was done to increase the mapping resolution and help to identify multiple loci associated with the traits. A regression mapping algorithm was applied for both mapping methods. The significance threshold levels of the logarithm of odds (LOD) scores (*P* ≤ 0.05) were assigned both by permutation tests in IM and manually assigned at a threshold of 3.0 in ICIM. Flanking markers, LOD scores, and percentage phenotypic variance explained (PVE%) are reported from the .RIC output file. QTL was named as q for QTL followed by the trait abbreviation, c for chromosome and the number of the chromosome. If more than one QTL was defined per chromosome for a specific trait then a point followed by a sequential number was used. A suffix ‘Nm’ was added to specify that the QTL was identified in Namikonga, or ‘A’ for Albert. All QTLs and markers are in version 5.1 of the cassava genome sequence, unless indicated.

### Identification of additional SNPs in the qCBSDRNc11 QTL region

To further investigate a QTL region of interest on chromosome XI, Namikonga and Albert whole genome sequence raw reads as available from Sequence Read Archive (SRA) of the National Centre for Biotechnology Information (NCBI) database were retrieved. Sequence quality was verified by FastQC and the quality-passed reads (in FastQ format) were aligned against version 6.1 of the cassava reference assembly [http://phytozome.jgi.doe.gov/pz/portal.html#!info?alias=Org_Mesculenta] using Bowtie2 genome alignment tool (Langmead et al. 2009). Alignment results were obtained in Sequence Alignment/Map (SAM) format and compressed into a Binary Alignment/Map (BAM) format using SamTools (Li et al. 2009). Markers defining the QTL region on chromosome XI were identified using BLASTn on v6.1 of the assembly (Supplementary Note 1). Genome annotation of the QTL region was done using vcf-annotate in the VCTtools package. Inferred protein-coding genes were characterized based on PFAM, PANTHER, KOG, KEGGORTH, GO, and TAIR domains. In addition, further SNPs were identified within the QTL region and SNP variation was characterized between Namikonga and Albert using a Genome Analysis Toolkit (GATK v3.3.0) (https://www.broadinstitute.org/gatk/) (Auwera et al. 2014). To provide confidence in SNP calling between the two genotypes, sequences from four additional genotypes (Kiroba, Muzege, Nachinyaya and AR37-80) were included.

## Results

### Population development and full sibling validation

A total of 10,238 pollinations were made in both crossing blocks from April to October 2010. From these pollinations, 2844 seeds were obtained, but only 876 seeds (30.8%) germinated. From the germinated seedlings, 806 were transplanted at Makutupora research center for bulking of the planting materials. Due to the outcrossing nature of cassava, each plant represented an independent genotype (Ceballos et al. 2004). At 3 MAP, 569 surviving individuals were genotyped using SSRs, and 305 individuals, which were confirmed to be Namikonga–Albert true progeny, were further genotyped by GBS. The remaining plants were off-types (98) and self (148) individuals.

### Construction of a genetic linkage map

GBS generated 3123 well-supported SNP markers across 252 Namikonga–Albert F1 progeny, after exclusion of three off-types (open pollination crosses) and nine self-derived individuals (S1), which had been further identified by GBS. In addition, 12 F_1_ individuals with more than 20% missing data were removed, leaving a population of 240 F_1_ individuals, which were used in the analysis. A high-density map with a length of 1776.2 cM consisting of 943 SNP markers was obtained with the highest marker density on chromosome IV (average 0.88 cM between markers) and the lowest on chromosome VIII (average 3.41 cM between markers) (Table 1; Supplementary Note 2). The average marker density was 1.88 SNPs per cM (Table 1). In addition, a framework genetic linkage map consisting of 243 SNP markers was generated (Supplementary Note 2). This map spanned 1784.0 cM, with an overall average marker interval ranging from 6.97 to 9.85 cM per linkage group (Supplementary Note 2). Both maps had 18 linkage groups, which corresponded to the 18 chromosomes of cassava (Table 1) (International Cassava Genetic Map Consortium 2015).

**Table 1:** Summary of a high-density genetic linkage map based on cassava genome assembly version 5.1 using the Namikonga × Albert mapping population

Chromosome (v5.1)	Number of mapped loci	Map length (cM)	Average distance between markers (cM)
I	58	131.7	2.27
II	86	122.5	1.42
III	44	91.4	2.08
IV	54	47.7	0.88
V	53	135.2	2.55
VI	56	77.7	1.39
VII	43	106.2	2.47
VIII	28	95.5	3.41
IX	63	99.1	1.57
X	64	111.5	1.74
XI	46	129.5	2.82
XII	41	108.3	2.64
XIII	44	76.0	1.73
XIV	43	65.7	1.53
XV	58	109.9	1.89
XVI	62	107.0	1.73
XVII	55	76.0	1.38
XVIII	45	85.5	1.90

### Phenotypic evaluation for response to CBSD and CMD

The two experimental locations, namely Chambezi and Naliendele, differed in CBSD severity. Experiments C1 and C2 (Chambezi) showed a much higher CBSD root necrosis mean score than N1 and N2 (Naliendele) (Supplementary Note 3). A similar trend was observed in the case of CMD (Supplementary Note 3). Within the same growing season, severity scores for CBSD root necrosis indicated high population means of 3.82 and 3.24 (i.e., 26–50% range of root necrotic area) in C1 and C2, as compared to 2.63 and 2.12 (i.e., less than 25% of root necrotic area) in N1 and N2, respectively (Table 2). For the case of CBSD foliar symptoms, the mean severity was low 1.14 (N1–3) (Naliendele season 1, 3 MAP) to 2.67 (C2–9), indicating mild expression of the symptoms (Table 2). An exception was observed at C1–6 where a higher mean severity score of 3.27 was obtained, and a non-significant (*P* > 0.05) normality test (*W* = 0.959) was obtained (Table 2). SWILK test results from GACD (v 1.1) basic statistics of the phenotypes revealed a highly significant normal distribution (*P* ≤ 0.001) (*W* ≥ 0.908 ≤ 0.941) for CBSD root necrosis mean scores in all experiments N1, N2, C1, and C2 (Table 2).

**Table 2:** GACD results showing basic statistics of the phenotypes, CBSDRN, CBSDF, and CMD obtained from the phenotyping experiments N1, N2, C1, and C2

Trait^a^	Experiment^b^	Population mean (1**–**5 scale)	Variance	SE^c^	SWILK normality (w)	*P* value
CBSDRN	N1	2.63	0.82	0.90	0.941	0.000***
	N2	2.12	0.82	0.91	0.908	0.000***
	C1	3.82	0.78	0.89	0.922	0.000***
	C2	3.24	1.19	1.09	0.940	0.000***
CBSDF	C1-3	1.04	0.01	0.11	0.487	0.000***
	C1-6	3.27	0.88	0.94	0.959	0.500*
	C1-9	1.91	0.62	0.79	0.892	0.000***
	N1-3	1.14	0.12	0.35	0.475	0.000***
	N1-6	1.29	0.26	0.51	0.632	0.000***
	N1-9	1.50	0.48	0.69	0.746	0.000***
	C2-3	1.19	0.08	0.28	0.727	0.000***
	C2-6	1.38	0.21	0.46	0.788	0.000***
	C2-9	2.67	1.18	1.08	0.942	0.000***
	N2-3	1.72	0.55	0.74	0.850	0.000***
	N2-6	1.52	0.62	0.79	0.697	0.000***
	N2-9	2.17	1.28	1.13	0.867	0.000***
CMD	C1-3	1.92	0.44	0.66	0.939	0.000***
	C1-6	2.32	0.63	0.80	0.938	0.000***
	C1-9	3.93	0.74	0.86	0.918	0.000***
	N1-3	2.31	0.97	0.98	0.928	0.000***
	N1-6	2.38	0.96	0.98	0.935	0.000***
	N1-9	2.04	0.75	0.87	0.894	0.000***
	C2-3	1.87	1.06	1.03	0.654	0.000***
	C2-6	2.29	1.30	1.14	0.885	0.000***
	C2-9	2.60	1.96	1.40	0.858	0.000***
	N2-3	1.95	0.92	0.96	0.832	0.000***
	N2-6	1.76	0.90	0.95	0.759	0.000***
	N2-9	1.64	0.61	0.78	0.783	0.000***

^a^ CBSDRN—CBSD root necrosis, CBSDF—CBSD foliar symptoms

^b^ N1 = Naliendele 2013, N2 = Naliendele 2014, C1 = Chambezi 2013, C2 = Chambezi 2014, -3 = 3MAP, -6 = 6MAP, and -9 = 9MAP

^c^ SE = standard error, * *P* ≤ 0.05; ** *P* ≤ 0.01; *** *P* ≤ 0.001

For CMD, Chambezi also had relatively high means of 3.93 (C1–9) and 2.60 (C2–9), while lower mean severity scores of 2.38 (N1–6) and 1.95 (N2–3) were observed at Naliendele at different time points (Table 2). SWILK normality tests revealed a moderate to highly significant normal CMD frequency distribution in all experiments N1, N2, C1, and C2 (*P* ≤ 0.001) (0.654 ≤ W ≤ 0.939) (Table 2). The parental genotype Namikonga expressed mild CBSD symptoms on leaves and roots with a maximum mean score of less than 2.0 across sites/seasons (less than 10% CBSD necrotic area), while Albert was highly affected with sites/seasons mean scores above class 3.0 (>26% CBSD necrotic area). The opposite was observed for CMD as Namikonga had a minimum mean score of 2.0 (2013) and maximum mean score of 5.0 (2014), while Albert showed milder symptoms with mean scores between 1.0 and 2.0 (Table 2).

Both CBSV and UCBSV were detected by qRT-PCR in plants that were randomly sampled from experiments C1 and N1 (Table 3). Results indicated that CBSV was more prevalent across the sites than UCBSV, although in most cases both viruses appeared as co-infections (Table 3). In Chambezi, 88.5% (69 out of 78 plants) of the tested plants were infected by at least one virus and in the rest of the tested plants (11.5%) neither virus was detected. A smaller proportion of plants (76.6%; 69 out of 90) were infected, according to diagnostics, in Naliendele as compared to Chambezi (Table 3). In addition, a few of the CBSV-negative plants were also free of CBSD foliar and root symptoms (16.7%) at N1, indicating possible escapes. At C1 all plants were either virus positive and/or had CBSD symptoms, indicating no escapes within the sample.

**Table 3:** Detection of CBSD-causing viruses in the sampled plants collected from C1 and N1

Experiment	CBSD score	Number of plants (%)					
		Number of plants tested	CBSV (%)	UCBSV (%)	CBSVs Co-infection (%)	CBSVs negative (%)	Possible CBSVs escapes* (%)
C1	1	6	3	0	3	0	0
	3	36	9	6	21	0	0
	5	36	3	0	24	9	0
	All	78	15 (19.2)	6 (7.7)	48 (61.5)	9 (11.5)	0 (0)
N1	1	36	9	3	9	15	15
	3	36	11	0	22	3	0
	5	18	6	0	9	3	0
	All	90	26 (28.9)	3 (3.3)	40 (44.4)	21 (23.3)	15 (16.7)

* Plants which were CBSD symptom free and CBSV or UCBSV negative according to real-time RT-PCR

### Identification of QTL associated with CBSD and CMD resistance

#### QTL associated with resistance to CBSD root necrosis

**Fig. 2:**
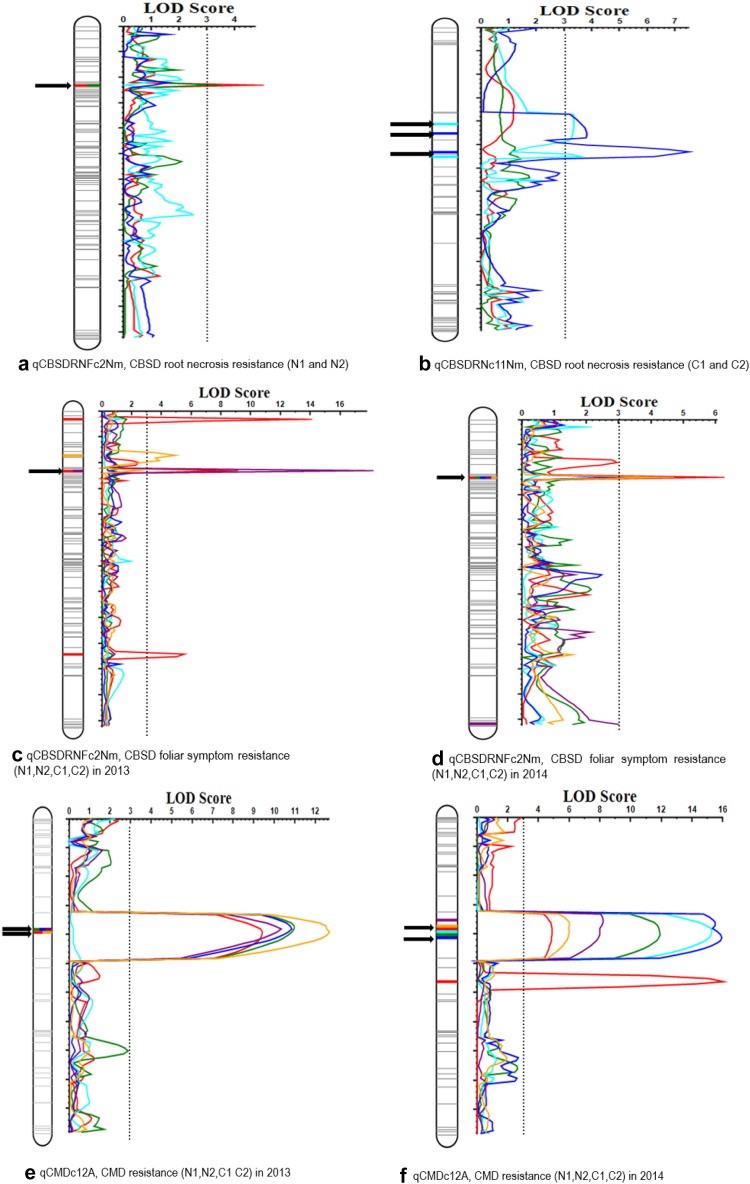
GACD LOD profile showing major QTL associated with resistance to CBSD root necrosis **a** qCBSDRNFc2Nm (N1 and N2) and **b** qCBSDRNc11Nm (C1 and C2); CBSD foliar symptoms **c** qCBSDRNFc2Nm (N1 and C1) and **d** qCBSDRNFc2Nm (N2 and C2); CMD symptoms **e** qCMDc12A (N1 and C1) and **f** qCMDc12A (N2 and C2). *Arrows* point to the QTL position

Mapping for resistance to root necrosis induced by CBSD infection in Namikonga identified two QTL with consistent flanking markers across seasons on chromosomes XI and II, qCBSDRNc11Nm and qCBSDRNFc2Nm, respectively (Fig. 2a, b; Table 4). A putative QTL was also detected on chromosome 18, qCBSDRNc18Nm, although the flanking markers at this QTL were less consistent across sites and seasons. qCBSDRNc11Nm was identified in both years in Chambezi under high disease pressure. The qCBSDRNc11Nm region stretched between two flanking markers, namely cXI:4502175 and cXI:4760631 (18.75 cM apart), corresponding to 5507842 and 5761172 bp in the v 6.1 cassava assembly. Three neighboring QTL peaks could be discerned within this region, although only one was consistent across seasons: qCBSDRNc11.1Nm (cXI:4527454–cXI:4617294 (v5.1)) (C2 only) with the highest LOD score, qCBSDRNc11.2Nm (cXI:4502175–cXI:4527454 (v5.1)) with the second largest LOD score (C1 and C2 only), and qCBSDRNc11.3Nm (cXI:4617294–cXI:4760631 (v5.1)) (C1 only) (Fig. 2b; Table 4). The highest LOD of 7.5 was detected at qCBSDRNc11.1Nm in experiment C2 with a percentage phenotypic variance explained (PVE) by the QTL of 17.39% in C2. Corresponding v6.1 markers, detected using BLASTn, can be found in Supplementary Note 1. Although the physical map distance between these markers (cXI:4527454–cXI:4617294 (v5.1)) was reasonably small (89.8 Kb), a relatively large distance on the genetic map was observed (6.2 cM)(14.5 kb/cM). In addition, between markers cXI:4502175 and cXI:4527454 at qCBSDRNc11.2Nm, there was a physical gap of 25.3 kb with a relatively large genetic map-based distance of 11.5 cM (2.2 kb/cM) (Fig. 2b; Supplementary Note 4). At qCBSDRNc11.3Nm, there was 143.3 Kb between markers (cXI:4617294–cXI:4760631 (v5.1)), yet a small genetic map distance of 1.05 cM (136.5 kb/cM). The theoretical distance is 434 kb/cM based on the current map distance and a genome size of 770 Mb (Awoleye et al. 1994). When the low-density map was used, although a QTL was detected in the same region in C1, C2, and N1, with maximum LOD 5.2, the map could not be extended below cXI:4527454 and there was a large gap between these markers and cXI:6227716 (Supplementary Note 5).

**Table 4:** QTL putatively associated with CBSD root necrosis resistance in ‘Namikonga’ consistently identified using phenotyping data in Chambezi and Naliendele field experiments during seasons 2013 and 2014

QTL name	Chromo.	Experiment^a^	Flanking markers (v 5.1)		Position (cM)	Parental effects			LOD	Unadjusted PVE (%)
			Left marker	Right marker		*F*	*M*	FM		
qCBSDRNFc2Nm	II	N1	cII:3454303	cII:3552915	22.1–23.7	0.30	0.32	0.50	4.76	47.38
qCBSDRNFc2Nm	II	N2	cII:3454303	cII:3552915	22.1–23.7	−0.44	0.26	0.27	3.24	42.46
qCBSDRNc11.1Nm	XI	C2	cXI:4527454	cXI:4617294	47.8–54.0	−0.34	0.24	0.21	7.50	17.39
qCBSDRNc11.2Nm	XI	C2	cXI:4502175	cXI:4527454	36.3–47.8	0.00	0.30	−0.02	3.81	7.54
qCBSDRNc11.2Nm	XI	C1	cXI:4502175	cXI:4527454	36.3–47.8	0.11	0.17	0.05	3.35	5.19
qCBSDRNc11.3Nm	XI	C1	cXI:4617294	cXI:4760631	54.0–55.0	−0.03	−0.04	0.20	3.60	5.32
qCBSDRNc18.1Nm	XVIII	C2	cXVIII:8650285	cXVIII:8943971	70.0–75.9	0.10	0.01	0.64	5.10	36.83
qCBSDRNc18.2Nm	XVIII	C2	cXVIII:3106706	cXVIII:3705640	23.8–27.8	0.18	0.15	0.42	3.99	16.10
qCBSDRNc18.3Nm	XVIII	N2	cXVIII:6320754	cXVIII:6502253	38.0–41.4	0.01	0.08	0.56	3.31	37.00

SNP marker names are as per *Manihot esculenta* v5.1 reference genome. Effect *F* is the estimated additive effect of the female parent (Namikonga). Effect *M* is the estimated additive effect of the male parent (Albert). Effect FM is the dominance effect between male and female markers

^a^ N1 = Naliendele 2013, N2 = Naliendele 2014, C1 = Chambezi 2013, C2 = Chambezi 2014 and PVE = % phenotypic variation explained

The estimated additive effect of the female parent (Namikonga) at qCBSDRNc11.1Nm was highest at −0.335 and that of the male parent (Albert) 0.2411 (Table 4). A negative additive effect was expected as the disease scoring scale was 1 (no symptoms) to 5 (maximum symptoms); therefore, the effect of resistant female parent should tend to reduce scores. The mean value of different QTL genotypes showed the largest difference between M(AC) 3.3832 and M(AD) 2.4715, where A and D are segregating in the female parent (Supplementary Note 6). The estimated additive effect of Namikonga at qCBSDRNc11.2Nm was 0.1136 (C1) and −0.0021 (C2), and for qCBSDRNc11.3Nm −0.0291 (C1) (Table 4).

A QTL, qCBSDRNFc2Nm, was consistently identified for resistance to root necrosis in both years at Naliendele under lower disease pressure with a maximum LOD = 4.76 (PVE = 13.36%), which was obtained in the N1 experiment (Table 4; Fig. 2a). qCBSDRNFc2Nm was flanked by markers cII:3454303 and cII:3552915 with 1.6 cM between the markers (Table 4). Interestingly, the additive effects of Namikonga and Albert were not consistent across locations at this QTL. An interesting putative QTL was detected on chromosome XVIII, qCBSDRNc18Nm, with two peaks detected towards the end of the left arm of the chromosome in C2, and another peak, located between them, detected in N2 (Table 4). Furthermore, additional putative QTLs were also identified on chromosomes III, IV, V, VI, VII, X, XII, XV, and XVI (Supplementary Notes 4 and 6).

#### QTL associated with resistance to CBSD foliar symptoms

QTL analysis to identify genomic regions that associate with resistance to CBSD foliar symptoms, on a high-density map, revealed several QTL on all chromosomes (Supplementary Note 7). The most interesting QTL was on chromosome 2, namely qCBSDRNFc2Nm. This QTL was detected using all site/season combinations and had a highest LOD = 17.81 (PVE = 4.6) detected at N1 (Fig. 2c, d; Table 5). It co-locates with the QTL for root necrosis, being flanked by the same markers (cII:3454303 and cII:3552915) and thus has both traits, root necrosis and foliar symptoms, indicated in its name (qCBSDRNFc2Nm). Additional QTL, namely qCBSDFc14Nm and qCBSDFc17Nm, were consistently identified in three out of the four experiments (Table 5). Inconsistent QTL associated with this resistance were identified on chromosomes III (qCBSDFc3Nm), VI (qCBSDFc6Nm), VIII (qCBSDFc8Nm), XI (qCBSDFc11Nm), XII (qCBSDFc12Nm), XVI (qCBSDFc16Nm), and XVIII (qCBSDFc18Nm) (Supplementary Note 7). Inconsistent QTLs were also identified when IM was performed on a low-density map (Supplementary Note 8).

**Table 5:** QTLs putatively associated with CBSD foliar resistance in ‘Namikonga’ consistently identified using phenotypic data from Chambezi and Naliendele field experiments during seasons 2013 and 2014

QTL	Chromosome	Experiment	Flanking markers (v5.1)		Position (cM)	Parental effects			LOD	Unadjusted PVE (%)
			Left marker	Right marker		*F*	*M*	FM		
qCBSDRNFc2Nm	II	N1-6	cII:3454303	cII:3552915	22.1–23.7	−0.33	0.31	0.31	17.81	74.39
qCBSDRNFc2Nm	II	C1-3	cII:3454303	cII:3552915	22.1–23.7	0.08	0.08	0.08	8.69	58.52
qCBSDRNFc2Nm	II	C2-3	cII:3454303	cII:3552915	22.1–23.7	−0.13	0.13	0.14	6.05	49.38
qCBSDRNFc2Nm	II	C2-6	cII:3454303	cII:3552915	22.1–23.7	−0.17	0.18	−0.19	3.34	35.54
qCBSDRNFc2Nm	II	N2-3	cII:3454303	cII:3552915	22.1–23.7	0.23	0.28	0.34	3.03	44.65
qCBSDRNFc2Nm	II	N2-6	cII:3454303	cII:3552915	22.1–23.7	−0.33	−0.35	0.35	4.08	45.34
qCBSDRNFc2Nm	II	N2-9	cII:3454303	cII:3552915	22.1–23.7	−0.36	−0.40	0.66	4.50	58.20
qCBSDFc14Nm	XIV	C1-3	cXIV:7870761	cXIV:8305501	31.8–35.7	0.12	0.12	0.11	14.32	58.65
qCBSDFc14Nm	XIV	N1-3	cXIV:7870761	cXIV:8305501	31.8–35.7	−0.26	−0.25	0.26	8.98	45.98
qCBSDFc14Nm	XIV	N1-9	cXIV:7870761	cXIV:8305501	31.8–35.7	−0.29	−0.22	0.30	3.90	31.80
qCBSDFc14Nm	XIV	C1-6	cXIV:7870761	cXIV:8305501	31.8–35.7	−0.32	−0.06	−0.06	3.19	10.40
qCBSDFc14Nm	XIV	C2-6	cXIV:7870761	cXIV:8305501	31.8–35.7	−0.32	−0.06	−0.06	4.77	26.89
qCBSDFc17Nm	XVII	N1-6	cXVII:15341988	cXVII:15982729	46.5–53.8	−0.33	−0.31	0.35	17.98	71.61
qCBSDFc17Nm	XVII	C2-3	cXVII:15341988	cXVII:15982729	46.5–53.8	−0.13	−0.14	0.18	4.13	52.43
qCBSDFc17Nm	XVII	N2-6	cXVII:15341988	cXVII:15982729	46.5–53.8	−0.31	−0.34	0.45	3.94	47.05

SNP marker names are as per *Manihot esculenta* v5.1 reference genome. Effect *F* is the estimated additive effect of the female parent (Namikonga). Effect *M* is the estimated additive effect of the male parent (Albert). Effect FM is the dominance effect between male and female markers

** N1 = Naliendele 2013; N2 = Naliendele 2014; C1 = Chambezi 2013; C2 = Chambezi2014; PVE = % explained variation

#### QTL associated with CMD resistance

**Fig. 3:**
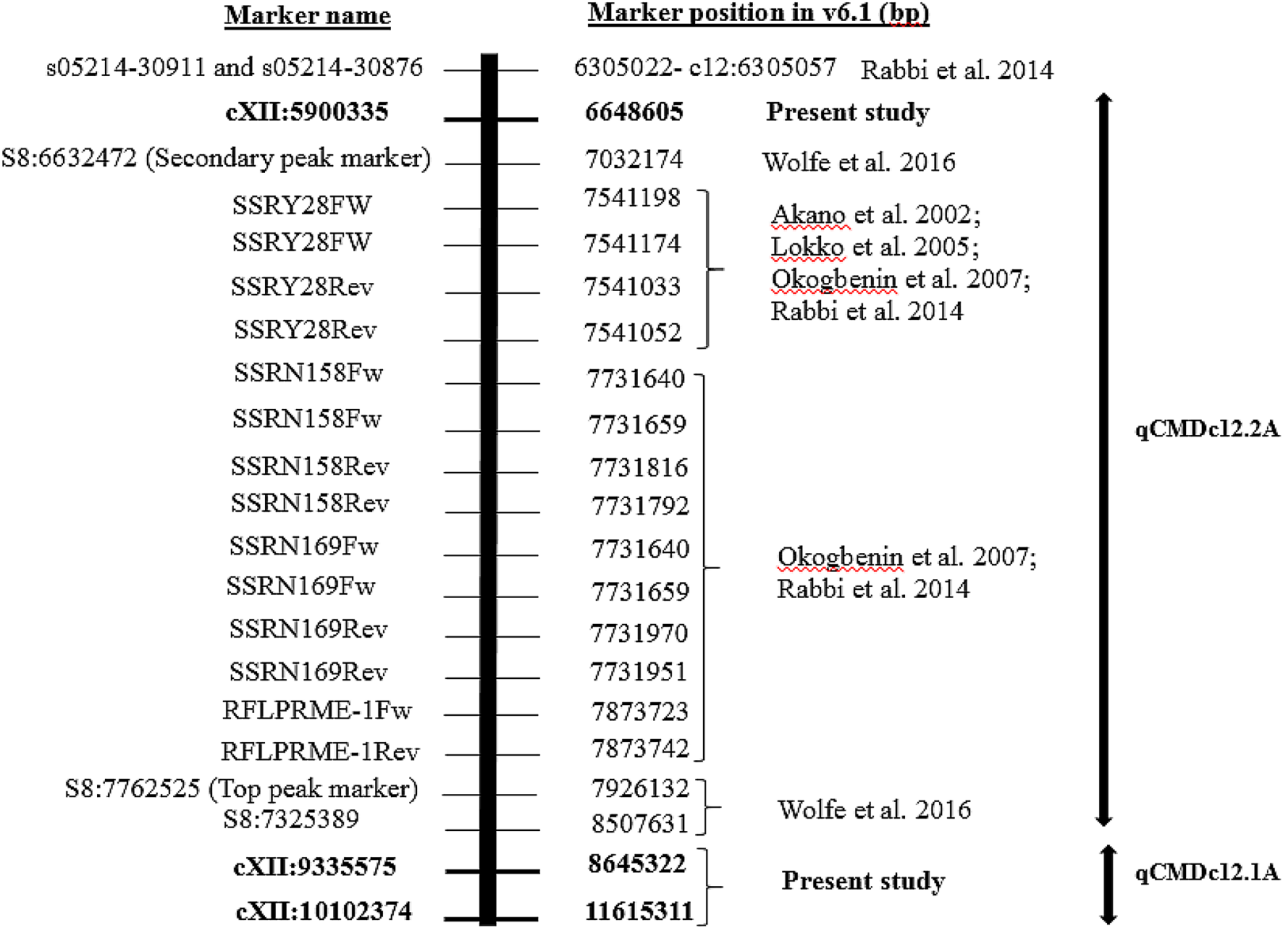
Positions of the earlier identified RFLP and SSR markers that tag the CMD resistance locus (CMD2) in West African germplasm with multiple QTL CMD resistance (qCMDc12.1A and qCMDc12.1A) identified in a Tanzanian landrace Albert (bolded) on chromosome 12 of the v6.1 cassava assembly

Albert, a parent of the bi-parental mapping population, is resistant to CMD and several significant QTLs associated with CMD resistance were detected. A major multiple QTL (qCMDc12A) was consistently detected on chromosome XII, in all site/season combinations and using phenotyping data collected at all time points: 3, 6, and 9 MAP (Fig. 2e, f). Initially, using a low-density map and IM in MapQTL v 6.0, qCMDc12A was thought to be a single QTL which spanned across a very large region of approximately 6.75 Mbp (30.53 cM) between markers cXII:3352898 and cXII:10102374 (Supplementary Note 9); however, greater resolution was achieved when using a high-density map and ICIM approach, as multiple QTL were detected. The qCMDc12A QTL had two peaks, designated as qCMDc12.1A and qCMDc12.2A. qCMDc12.1A was flanked by markers cXII:9335575 and cXII:10102374 having a maximum LOD = 15.92 (PVE = 16.43%), obtained in C2 at 3 MAP (Table 6). The second peak (qCMDc12.2A) was flanked by markers cXII:5900335 and cXII:9335575 and had a peak LOD of 10.95 (PVE = 13.51%) obtained at C1 at 6 MAP. In addition, ICIM reduced the total length of the qCMDc12A QTL, covering both QTL qCMDc12.1A and qCMDc12.2A, between cXII:5900335 and cXII:10102374, to 4.97 Mbp (~15.8 cM) (Fig. 3; Supplementary Note 10). Based on v6.1 of the cassava genome assembly, the qCMDc12.1A QTL region lies between 8645322 and 11615311 bp and qCMDc12.2A between 6648605 and 8645322 bp (Fig. 3). Estimated additive effects of the male and female parents and dominance effects between the male and female parents can be found in Supplementary Note 6.

**Table 6:** QTLs putatively associated with CMD resistance identified using phenotypic data from Chambezi and Naliendele field experiments during seasons 2013 and 2014

QTL	Chromosome	Experiment	Flanking markers (v5.1)		LOD	Unadjusted PVE (%)
			Left marker	Right marker		
qCMDc12.1A	XII	N1-9	cXII:9335575	cXII:10102374	12.68	23.84
qCMDc12.1A	XII	C1-3	cXII:9335575	cXII:10102374	9.39	15.00
qCMDc12.1A	XII	N2-3	cXII:9335575	cXII:10102374	15.92	27.01
qCMDc12.1A	XII	C2-9	cXII:9335575	cXII:10102374	15.29	27.33
qCMDc12.1A	XII	C2-6	cXII:9335575	cXII:10102374	11.90	19.01
qCMDc12.2A	XII	C1-6	cXII:5900335	cXII:9335575	10.95	14.32
qCMDc12.2A	XII	N1-3	cXII:5900335	cXII:9335575	10.83	20.38
qCMDc12.2A	XII	N1-6	cXII:5900335	cXII:9335575	10.32	20.61
qCMDc12.2A	XII	N2-6	cXII:5900335	cXII:9335575	8.20	16.32
qCMDc12.2A	XII	N2-9	cXII:5900335	cXII:9335575	6.03	14.04
qCMDc12.2A	XII	C2-3	cXII:5900335	cXII:9335575	4.90	4.46
qCMDc12.3A	XII	C2-3	cXII:10022137	cXII:11747091	16.00	50.37
qCMDc10.1A	X	N2-3	cX:109425	cX:690891	3.12	6.56
qCMDc10.2A	X	C1-9	cX:1986981	cX:2500662	5.93	10.15
qCMDc10.3A	X	N2-6	cX:8151902	cX:8859608	4.30	17.35
qCMDc10.3A	X	N2-9	cX:8151902	cX:8859608	3.53	23.90
qCMDc10.4A	X	C1-3	cX:8859608	cX:9048199	3.57	5.93
qCMDc1.1A	I	N2-9	cI:12617571	cI:14956077	3.07	7.66
qCMDc1.2A	I	N2-9	cI:15891396	cI:16140931	3.64	7.97
qCMDc1.3A	I	C2-9	cI:16444969	cI:17133348	3.25	5.18
qCMDc6A	VI	N2-3	cVI:14734153	cVI:18400898	4.78	19.27
qCMDc6A	VI	N2-6	cVI:14734153	cVI:18400898	4.25	11.22
qCMDc5.1A	V	N1-3	cV:6374034	cV:6348932	3.70	11.17
qCMDc5.1A	V	N2-3	cV:6374034	cV:6348932	5.39	7.60
qCMDc5.2A	V	C2-9	cV:7989459	cV:8525487	3.11	4.44
qCMDc5.3A	V	C1-6	cV:9268941	cV:10924271	9.71	14.05
qCMDc3A	III	C2-6	cIII:20233083	cIII:17215597	7.92	16.33
qCMDc3A	III	C2-9	cIII:20233083	cIII:17215597	4.10	10.67
qCMDc2.1A	II	C2-3	cII:4722413	cII:6114475	3.68	7.03
qCMDc2.2A	II	N2-3	cII:13956600	cII:14143547	3.41	5.47

Only QTL on chromosomes that are significant at more than one sampling time or site are given

Information for additional putative QTL is given in Supplementary Note 6

** N1 = Naliendele 2013, N2 = Naliendele 2014, C1 = Chambezi 2013, C2 = Chambezi 2014, -3 = 3MAP, -6 = 6MAP, -9 = 9MAP and PVE = % phenotypic variation explained

With the exception of the qCMDc5A (chromosome V), which was identified at Naliendele in both seasons, thirteen additional putative QTL associated with CMD resistance in Albert were detected in one site and season only: chromosomes I (qCMDc1A), II (qCMDc2A), III (qCMDc3A), IV (qCMDc4A), VI (qCMDc6A), VIII (qCMDc8A), IX (qCMDc9A), X (qCMDc10A), XIII (qCMDc13A), XV (qCMDc15A), XVI (qCMDc16A), XVII (qCMDc17A), and XVIII (qCMDc18A) (Supplementary Note 10). The additional regions were inconsistently identified across sites/seasons.

### Relationship between qCMDc12A and the CMD2 locus

To compare the position of qCMDc12A with previously identified CMD QTL, BLASTn was used to position SSR markers (SSRY28, SSRY106, SSRN198, SSRN158, SSRN169); a SCAR marker, namely RFLPRME-1 (Akano et al. 2002; Lokko et al. 2005; Okogbenin et al. 2007; Rabbi et al. 2014) and SNP markers (s05214:30911 and s05214:30876) (Rabbi et al. 2014) reported earlier to be associated with CMD2, on v6.1 of the cassava genome sequence (Fig. 3). The genomic regions associated with moderate and strong CMD resistance, recently reported by Wolfe et al. (2016) were also included. The QTL identified here spanned the majority of these markers, although the peak at 8645322 bp (v6.1) was within 150 kb of a Wolfe et al. (2016) flanking marker S8:7325389 at 8507631 bp (v6.1) (Fig. 3). In addition RFLPRME-1 reverse SCAR primer was only 0.77 Mbp from cXII:9335575 (v5.1) positioned at 8645322 bp (v6.1), which is the left flanking marker of qCMDc12.1A and the right flanking marker of qCMDc12.2A (Fig. 3).

### Sequence variation between Namikonga and Albert in genomic regions associated with CBSD root necrosis

#### Identification of additional SNP markers in the qCBSDRNc11Nm QTL region

**Fig. 4:**
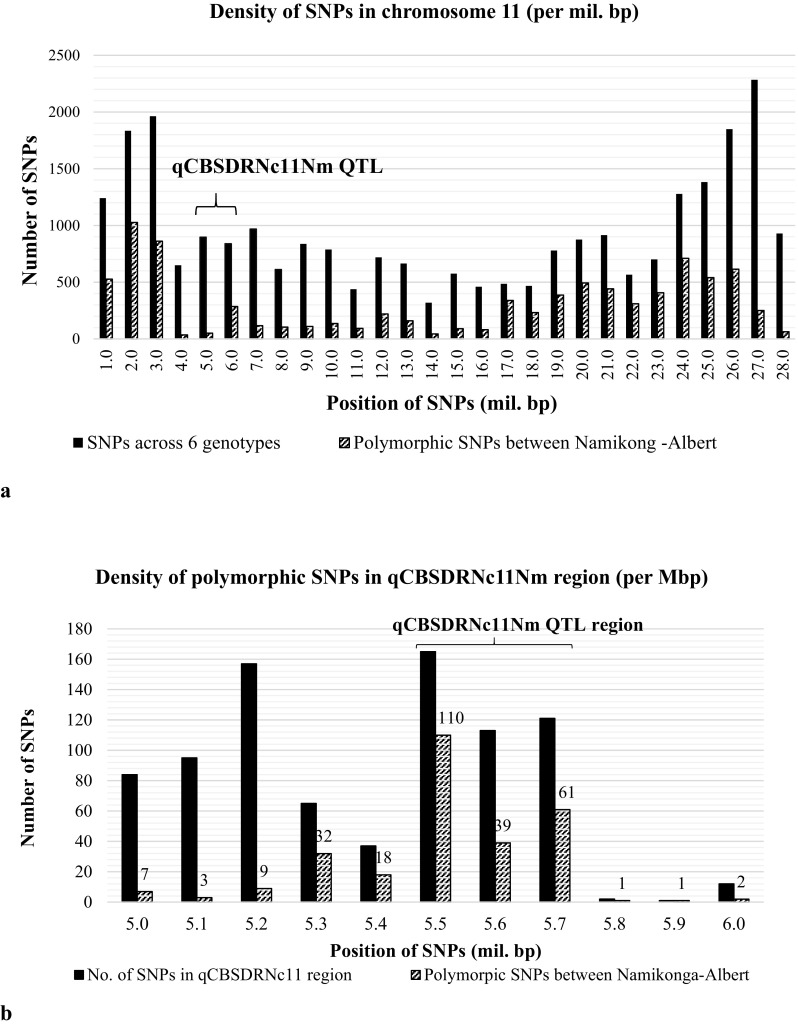
SNP density **a** across chromosome 11 (27 Mb) and **b** qCBSDRNc11Nm region between 5507842 and 5761172 bp (v6.1)

**Fig. 5:**
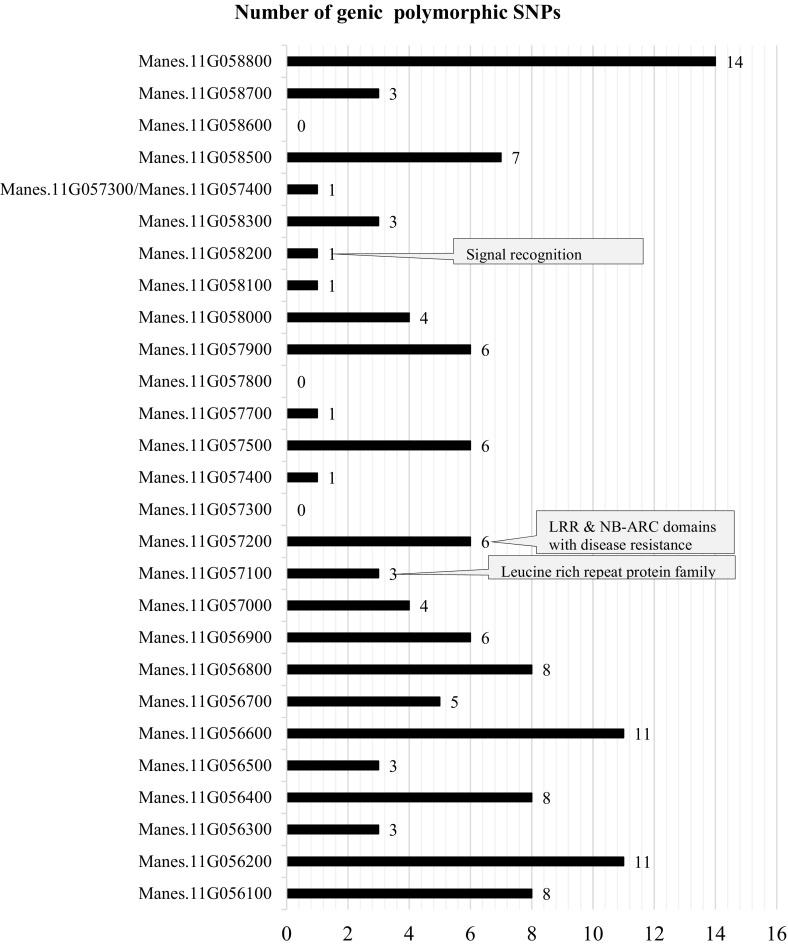
Genes that are linked to SNPs polymorphic between Namikonga and Albert in the qCBSDRNc11Nm region

After alignment of the six genotypes to the reference genome (v6.1) and SNP calling using GATK, 1,338,691 genome-wide SNP markers were identified, of which 26,320 (~2.0%) were located on chromosome XI, where the main CBSDRN QTL is located (Fig. 4a). Of these SNPs only 1.36% (i.e., only 359 SNPs of the total genome-wide SNPs) were identified within the qCBSDRNc11Nm region, between cXI:4502175 and cXI:4760631, corresponding to 5507873–5761172 bp (v6.1) (Supplementary Note 11). Approximately 51.5% (185 out of 359) of SNPs identified in the qCBSDRNc11Nm region were polymorphic between the closely related target parental genotypes Namikonga and Albert (Supplementary Note 11) [hpc.ilri.cgiar.org/emasumba/Genotypes2015_filter_only.vcf], among which only 46.3% (124 SNPs) were genic SNPs linked to 24 annotated genes (Fig. 5).

#### Estimation of SNP density within the qCBSDRNc11Nm QTL region

SNP density was higher at both ends of chromosome 11, as opposed to the centromeric region (Fig. 4a). However, the low density of polymorphic SNPs between Namikonga and Albert appeared to extend into the region between 4.0 and 16.0 Mbp, with a moderate increase in SNP density in the qCBSDRNc11Nm region from 5.5 to 5.8 Mbp (v6.1) (Fig. 4a). A closer examination of the qCBSDRNc11Nm QTL region revealed nearly twice the density of SNP markers (0.07 SNPs/100 bp; 210 SNPs across 0.3 Mbp) polymorphic between the parental genotypes Namikonga and Albert (Fig. 4b), compared to the overall mean SNP density on chromosome 11 (0.03 SNPs/100 bp; 8738 SNPs across 28.0 Mbp).

#### Genic SNPs within the qCBSDRNc11Nm region, their characteristics and association with disease resistance

A total of 27 genes were identified within the qCBSDRNc11Nm QTL region (5507842–5761172 bp v6.1) (Supplementary Note 12) with 24 containing SNPs that were polymorphic between Namikonga and Albert. Of these 24 genes, two were leucine-rich repeat (LRR) protein-coding genes that are annotated as non-host-specific defense-related processes (Torii 2004). These genes were Manes.11G057100.v6.1 and Manes.11G057200.v6.1 (Fig. 5, Supplementary Note 12) and contain nine SNPs polymorphic between Albert and Namikonga (Table 7). These genes contribute towards signal transduction and virus recognition activity, which are amongst the most important roles of R proteins of the LRR–NBS type (Supplementary Note 13) (Belkhadir et al. 2004; Forsthoefel et al. 2005). Nine of the ten R-gene-linked SNPs (loci) identified in the qCBSDRNc11Nm region were heterozygous (0/1) for Namikonga and homozygous (1/1) for Albert (Table 7). In addition, another gene (Manes.11G058200) which encodes a signal recognition protein (SRP) was linked to one of the polymorphic SNP markers positioned at 5583187 bp in the qCBSDRNc11Nm QTL region (Fig. 5) (Supplementary Note 13). Only two of the ten SNPs, one in each of the LRR genes, were non-synonymous, having moderate effect (Table 7).

**Table 7:** Characteristics and position of polymorphic SNPs between Namikonga and Albert within R-genes in the qCBSDRNc11Nm QTL region

SNP position in bp (v6.1)	Gene	Encoding protein	Genic region	Segregation type		Effect prediction	Predicted effect strength	Predicted change
				Namikonga	Albert			
c11:5574099	Manes.11G057100	LRR	CDS	0/1	1/1	Coding; non-synonymous, missense	Moderate	Ttc±/Ctc; F23L
c11:5574320	Manes.11G057100	LRR	CDS	0/1	1/1	Coding; synonymous, silent	Low	gaC/gaT; D96
c11:5574671	Manes.11G057100	LRR	CDS	0/1	1/1	Coding; synonymous, silent	Low	aaA/aaG; K213
c11:5579072	Manes.11G057200	LRR NB-ARC	5′ UTR	0/1	1/1	Non-coding	–	–
c11:5579208	Manes.11G057200	LRR NB-ARC	5′ UTR	0/1	1/1	Non-coding	–	–
c11:5579409	Manes.11G057200	LRR NB-ARC	Intron	0/1	1/1	Non-coding	–	–
c11:5579541	Manes.11G057200	LRR NB-ARC	Intron	0/1	1/1	Non-coding	–	–
c11:5579630	Manes.11G057200	LRR NB-ARC	Intron	0/1	1/1	Non-coding	–	–
c11:5583187	Manes.11G057200	LRR NB-ARC	CDS	1/1	0/0	Coding; Non-synonymous, missense	Moderate	Gat/Aat; D740 N
c11:5663225	Manes.11G058200	Signal recognition particle	Intron	0/1	1/1	Non-coding	–	–

## Discussion

A relatively large population of 240 F_1_ individuals, phenotyped across two sites in two successive seasons, was used to identify QTL associated with the two most devastating virus diseases of cassava in South, East, and Central Africa. Two QTLs and a third putative QTL associated with CBSD root necrosis resistance were identified in the Tanzanian cassava variety Namikonga. In addition, three consistent QTLs associated with CBSD foliar symptom resistance were identified. Once validated, markers underlying these QTLs could be used in genomic breeding approaches to preemptively select for CBSD resistance in West Africa ahead of the disease pandemic front, with important implications for food security in the region. These markers could also be used for marker-assisted breeding in regions such as South, East, and Central Africa which are already affected by CBSD. Here we also identified SNP markers linked to two closely positioned QTLs associated with CMD resistance, one of which co-locates with the previously identified CMD2 locus detected in West African germplasm. This is the first time that the CMD2 locus has been detected in an East African landrace.

Cassava is a highly heterozygous and heterogeneous outcrossing crop with a breeding cycle of one year (Ceballos et al. 2004). It is known to have a large deleterious genetic load and suffers high levels of inbreeding depression (Ceballos et al. 2010). For these reasons, QTL mapping is largely done in F_1_ populations (Hayashi and Awata 2004), although occasionally F_2_ populations have been generated (Tong et al. 2012). The advantages of an F_1_ population are that it is relatively quick to generate (one season/year) and a reasonable population size can be obtained, although it is difficult to detect purely recessive QTL that requires the homozygous state for expression.

Poor germination rates were achieved, which was likely due to high diurnal temperature ranges in the screen house (over 50 °C day temperatures and 19 °C night temperatures). Germination was attempted in pots on benches, thereby unintentionally allowing the soil temperatures to fluctuate more easily with air temperatures. Once pots were placed on the ground, protecting the soil, higher germination rates were achieved. This could have imposed some bias on the population. The population was further reduced when a large number of off-types and self-individuals were detected. This is likely due to the fact that flowers were not covered (bagged) for up to 3 days following pollination, thereby allowing unintended pollinations.

Both CBSVs were detected in both phenotyping sites; however, CBSD-related trait mean scores indicated that the disease pressure was much lower in N1 and N2 compared to C1 and C2 (Table 2). No significant departure from a normal distribution was detected for CBSD-related traits with adequate segregation in both sites. This indicates a lack of complete dominance or recessiveness in genetic control, or that multiple genes are involved. The QTL qCBSDRNc11Nm associated with resistance to CBSD root necrosis was consistently detected at Chambezi in both seasons, but not in Naliendele, whereas qCBSDRNFc2Nm was associated with root necrosis resistance in Naliendele but not Chambezi. This discrepancy could be attributed to differences in disease pressure and/or differences in virus strains. In fact, a new CBSV sub-population, tentatively called CBSV-Tanzania (CBSV-TZ), has been identified predominantly in southern Tanzania (including the Naliendele phenotyping site) and in Malawi (Mbewe et al. 2017). It is interesting to note that qCBSDRNFc2Nm was associated with resistance to CBSD foliar symptoms in all four environments (sites and seasons) (Supplementary Note 7), a trait which gave inconsistent results when a low-density map was used with IM (Supplementary Note 8). The highest LOD for this trait was 17.8 explaining 74.39% PVE.

The QTL qCBSDRNc11.2Nm on chromosome 11 associated with resistance to CBSD root necrosis had the highest LOD score of 7.50, explaining 17.3% of the phenotypic variation. Although three QTLs are indicated in this region, further analysis is required to confirm this observation. It is interesting to note that a large genetic distance is indicated for a small physical distance. Although there is no constant ratio to convert cM to bp, cM is an estimate of the likelihood of recombination within an interval, so here we expect a high rate of recombination, or ‘recombination hotspot’ or alternatively an error in the map, although marker order is consistent with the integrated map for cassava (International Cassava Genetic Map Consortium 2015).

Kulembeka (2010) using SSR markers and a different Namikonga x Albert F_1_ population with phenotyping in Chambezi and Naliendele over two seasons found an association of the SSR marker NS945 with CBSD root necrosis. NS945 is positioned on chromosome 4 from 565803 to 566195 bp (v6.1). Further SNP genotyping using a Goldengate assay (Illumina) (Rabbi et al. 2012) and QTL analysis using IM in MapQTL v6 (van Ooijen 2009) and using the same phenotyping data, on only 60 genotypes, identified a QTL with a peak defined by flanking markers Me.MEF.c.1513 (LOD 4.11, PVE27.8) and Me.MEF.c.2120 (LOD 4.09, PVE 27.7) (Ferguson, per. comm.) which are located at 5551588 and 5508564 bp (v6.1) on chromosome 11 (Supplementary note 15). These markers which span 43 kb lie within the 122-kb region of qCBSDRNc11.1Nm and qCBSDRNc11.2Nm, supporting results of the current study. It is interesting to note that chromosomes 4 and 11 are homeologous chromosomes (Bredeson et al. 2016).

Besides qCBSDRNc11Nm and qCBSDRNFc2Nm, a putative QTL was identified on chromosome XVIII associated with root necrosis. This QTL occurred over a rather dispersed area with two peaks detected in C2 between 3106706 and 3705640 bp, and 8650285 and 8943971 bp, and a third peak, between the other two, in N2 between 6320754 and 6502253 bp (Table 3). Although the peaks detected are inconsistently positioned, they are close enough to warrant attention. Additional inconsistently positioned peaks with minor effects on CBSD root necrosis resistance in Namikonga were identified on chromosomes III, IV, V, VI, VII, X, XII, XV, and XVI. Results here are consistent with a diallel study conducted by Kulembeka et al. (2012) which indicated that CBSD resistance was quantitative, being controlled by at least two genes of minor effect which are additive in nature.

When parental genotypes Albert and Namikonga were first selected, the genetic relationship between these varieties was unknown. It later became apparent from the alignment of whole genome sequence that these genotypes are genetically related. Albert is a putative full-sib of the Nigerian cassava landrace TME117 and Namikonga has a parent–offspring relationship with the same genotype, TME 117 (Bredeson et al. 2016). Despite the overall low density of polymorphic SNPs between Namikonga and Albert, an increase in SNP density was observed at a region close to the most significant qCBSDRNc11Nm locus, cXI:4527454. Almost 52.6% (210 out of 399 SNPs) of the GATK-based SNPs which were identified in the qCBSDRNc11Nm region between 5.5 and 5.7 Mbp (v6.1) were polymorphic between the parental genotypes clustered in this region, indicating past recombination events. Of the 27 genes found within this region (Supplementary Note 11 and 12), 24 contained SNPs that were polymorphic between Namikonga and Albert. Three annotated cassava genes, Manes.11G057100, Manes.11G057200, and Manes.11058200, were of particular interest in terms of potentially contributing to the observed CBSD root necrosis resistance. Manes.11G057100 and Manes.11G057200 encode LRR proteins that are associated with signal transduction in plant defense-related processes and each contains one non-synonymous SNP (Forsthoefel et al. 2005; Torii 2004). The third gene, Manes.11G058200, encodes SRP, which in eukaryotes binds to the signal sequence of a newly synthesized peptide as it emerges from the ribosome. This binding leads to “elongation arrest”, which is a slowing down of protein synthesis. This warrants further investigation as reduced CBSV load has been recognized in Namikonga, which indicates inhibition of viral replication (Kaweesi et al. 2014; Maruthi et al. 2014) and the fact that disruptive binding of elongation initiation factors (EIf) is the most common form of resistance to Potyviruses (Truniger and Aranda 2009). The majority of genic polymorphic SNPs segregated in the female parent, Namikonga. These could efficiently be used in MAS either for controlled or open-pollinated populations, as their allelic segregation in Namikonga facilitates segregation in F_1_ which is the generation at which most cassava breeders do selections.

To date, three loci associated with CMD resistance have been reported: CMD1 (Fregene et al. 2000), CMD2 (Akano et al. 2002; Lokko et al. 2005; Rabbi et al. 2014), and CMD3 (Okogbenin et al. 2012). The CMD2 locus was identified in West African cassava landraces TME3 and TME7 (now TMEB3 and TMEB7, respectively) (Akano et al. 2002; Lokko et al. 2005; Rabbi et al. 2014) and validated in other West African improved genotypes, namely TMS-97/2205 and TMS-98/0505 (now TMS-I972205 and TMS-I980505, respectively) (Okogbenin et al. 2012). CMD2 was initially referenced by several SSR (SSRY28, SSRY158 and SSRY169) and SCAR (RFLPRME-1) markers, and more recently by SNP markers (Rabbi et al. 2014; Wolfe et al. 2016). CMD3, which co-locates with SSR marker NS198, was also identified in IITA improved genotypes TMS 97/2205 and TMS 98/0505 (Okogbenin et al. 2012). BLASTn positioned the genomic regions that are associated with both CMD2 and CMD3 loci on chromosome 12 (v6.1) of the cassava genome assembly. The present study detected a highly consistent QTL, qCMDc12A, associated with CMD resistance in the East African variety, Albert, located on the same chromosome. The largest peak of qCMDc12A is very close to marker cXII:9335575 positioned at 8645322 bp (v6.1). This marker is only 0.77 Mbp away from the SCAR marker RFLPRME1-Rev identified earlier as tagging CMD2 (Akano et al. 2002; Lokko et al. 2005) (Fig. 3), indicating that these loci are likely to be the same. The CMD3 marker, NS198, is located over 5.0 Mbp (between 1353175 and 1353375 bp v6.1) from qCMDc12A (Supplementary Note 14). The field performance of Albert in Tanzania (Mtunda et al. 2003; Rwegasira and Rey 2012) is consistent with strong field resistance to CMD infection, which is characteristic of the CMD2 locus. This is the first time that this locus has been identified in an East African landrace. However, it is possible that the resistance was originally derived from West African germplasm, as Albert is a full-sib of West African landrace TME117 or closely related genotype (Bredeson et al. 2016), although TME117 is susceptible to CMD. Cassava clones were brought to the Amani breeding program in Tanzania, from all over the world, including West Africa (Nichols 1947). This could explain the early movement of the CMD2 locus from West Africa to East Africa.

Wolfe et al. (2016), through a large genome-wide association study (GWAS), identified one genomic region of large effect at S8:7762525 (7926132 bp; v6.1) and a second closely located and interacting region from S8:6632472 (7032174 bp; v6.1) to S8:4919667 (4953572 bp; v6.1). It appears that the large genomic region covered by qCMDc12A identified in this study [~4.97 Mb; 6648605 bp to 11615311 bp (v6.1)] encompasses two very closely positioned QTL, supporting the findings of Wolfe et al. (2016). qCMDc12.1A has flanking markers cXII:9335575 [8645322 bp (v6.1)] and cXII:10102374 [11615311 bp (v6.1)] close to the Wolfe et al. (2016) main peak marker at 7926132 bp (v6.1). The second QTL, qCMDc12.2A, is flanked by cXII:5900335 [6648605 bp (v6.1)] and the same marker as qCMDc12.1A, cXII:9335575 [8645322 bp (.v6.1)] qCMDc12.2A, is just 0.89 Mb from one of the CMD2 tagging markers, SSRY28, earlier reported by Akano et al. (2002) and 0.34 Mb from the two GBS-SNPs s05214:30911 and s05214:30876 identified by Rabbi et al. (2014). The identification of two QTLs within the CMD2 region could indicate the presence of two loci or reflect different allelic forms of the same locus. In addition, as in this study, Wolfe et al. (2016) identified a number of other QTL of small effect, distributed across many chromosomes.

In conclusion, a significant QTL associated with resistance to CBSD-induced root necrosis, qCBSDRNc11Nm, was detected on chromosome XI and was consistent across two seasons in a high-CBSD pressure site at Chambezi in coastal Tanzania. A second QTL on chromosome II (qCBSDRNFc2Nm), associated with resistance to both CBSD root and foliar symptoms, was detected in a second site, Naliendele, in southeastern Tanzania. Results indicate that QTL affecting root necrosis and foliar symptoms may be different, although not exclusively so. Once validated, markers underlying these QTL will be useful for genomic-based approaches to breeding, including MAS, both in CBSD-affected areas as well as in a pre-emptive manner in areas yet unaffected. Interestingly, the peak of the qCBSDRNc11Nm QTL coincided with a region of unexpectedly high SNP density and polymorphism between the two closely related parents, Namikonga and Albert, indicating a region of past recombination. A number of candidate resistance genes were identified within this region, including two LRR genes and a gene encoding a signal recognition protein. These should be further investigated for causative effect. In addition, for the first time, two QTL which co-locate with the earlier identified CMD2 locus, namely qCMDc12.1A and qCMDc12.2A, have been identified, and these have been found in an East African landrace, Albert.

### **Author contribution statement**

EAM: conducted the study, collected phenotyping data, analyzed the data and drafted the manuscript; FK: assisted with phenotyping data collection; GM: project manager from Agriculture Research Institute, Tanzania, financial and logistical support; SK: assisted with phenotyping data collection; HK: planning of phenotyping trials; SR: provided scientific and bioinformatics support; JVB: filtering and calling of SNP data, identification of off-types, and selfs; JBL: conducted genotyping-by-sequencing; DSR: technical advice on GBS, manager of UC-Berkeley/JGI project component; EK: advice on the selection of genotypes and phenotyping trials; MSK: training and technical support on bioinformatics analyses; AAM: scientific guidance; NAvdM: advice and editorial support; MEF: conceived the study, coordinated research activities, and provided technical, analytical and editorial support.

## Electronic supplementary material

Below is the link to the electronic supplementary material.

Note 1: BLASTn results - qCBSDRNc11Nm, qCBSDRNFc2Nm and qCMDc12A flanking markers (v5.1) to (v6.1) (XLSX 21 kb)

Note 2: Map and genotype data of the low- and high-density maps (XLSX 993 kb)

Note 3: Frequency distributions and basic statistics of the traits CBSDRN, CBSDFOL and CMD obtained from the phenotyping experiments N1, N2, C1 and C2 (DOCX 191 kb)

Note 4: GACD based LOD profiles showing the QTL regions associated with CBSD root necrosis resistance in ‘Namikonga’ (DOCX 560 kb)

Note 5: MapQTL Profiles showing the QTL regions putatively associated with CBSDRN resistance in ‘Namikonga’ based on N1, N2, C1 and C2 phenotyping experiments (DOCX 50 kb)

Note 6: GACD outputs presenting QTL that are associated with the resistance to CBSD-induced root necrosis (qCBSDRN) and CMD (qCMD) in Namikonga and Albert (XLSX 23 kb)

Note 7: GACD outputs presenting QTL that are associated with the resistance to CBSD foliar symptoms (qCBSDRNF and qCBSDF) in ‘Namikonga’ (XLSX 25 kb)

Note 8: MapQTL outputs showing QTL associating with CBSD foliar symptoms resistance in ‘Namikonga’ (DOCX 98 kb)

Note 9: MapQTL Profiles showing QTL putatively associated with CMD resistance in ‘Albert’ (DOCX 86 kb)

Note 10: GACD LOD Profiles showing the genomic regions in Namikonga-Albert F_1_ individuals that are associated with CMD resistance (DOCX 1563 kb)

Note 11: List of SNPs identified in qCBSDRNc11Nm QTL region (XLSX 61 kb)

Note 12:List of genes linked to SNPs identified in a narrowed qCBSDRNc11Nm QTL region on a high density map (XLSX 14 kb)

Note 13:Functional annotation of genes linked to SNPs identified in qCBSDRNc11Nm QTL region (XLSX 16 kb)

Note 14:BLASTn results showing the relationship between monogenic CMD resistance CMD2 and the newly identified quantitative CMD resistance (qCMDc12A) (XLSX 19 kb)

Note 15: BLASTn results showing positions, LOD and PVE (%) of the previously identified SNP markers associated with CBSD resistance in Namikonga (Kulembeka, Unpublished work) (XLSX 12 kb)
