# Supplementary material for: QTL associated with resistance to cassava brown streak and cassava mosaic diseases in a bi-parental cross of two Tanzanian farmer varieties, Namikonga and Albert
Source: Theor Appl Genet. 2017 Jul 13;130(10):2069–90. doi: 10.1007/s00122-017-2943-z (PMC5606945; doi:10.1007/s00122-017-2943-z)
Supplement: Supplementary file 3 — Note 3: Frequency distributions and basic statistics of the traits CBSDRN, CBSDFOL and CMD obtained from the phenotyping experiments N1, N2, C1 and C2 (DOCX 191 kb) [file 122_2017_2943_MOESM3_ESM.docx]

**Supplementary Note 3**

**Article title**: QTL associated with resistance to cassava brown streak and cassava mosaic diseases in a bi-parental cross of two Tanzanian farmer-varieties, Namikonga and Albert

**Journal Name**: Theoretical and Applied Genetics

**Author names**: E. A. Masumba, F. Kapinga, G. Mkamilo, S. Kasele, H. Kulembeka, S. Rounsley, J. V. Bredeson, J. B. Lyons, D. S. Rokhsar, E. Kanju, M. S. Katari, A. A. Myburg, N. A. van der Merwe and M. E. Ferguson

**Affiliation and email of corresponding author:** Morag Ferguson, International Institute of Tropical Agriculture (IITA), P.O. Box 30709, Nairobi 00100, Kenya; m.ferguson@cgiar.org


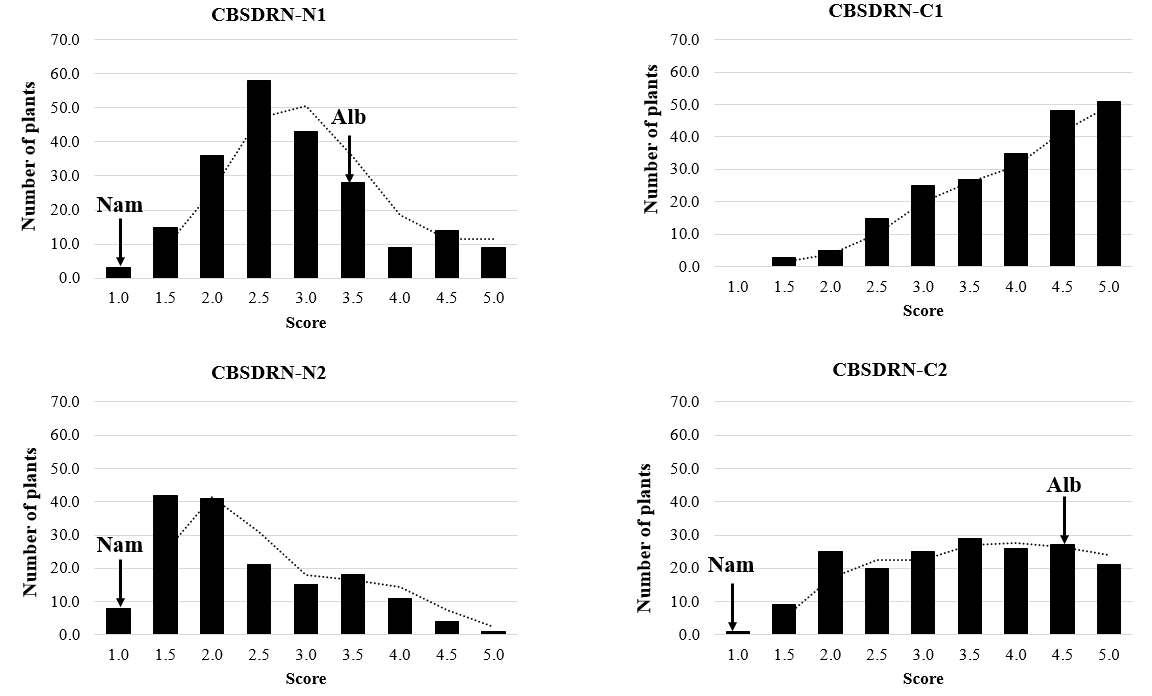


N1=Naliendele season 1, N2=Naliendele season 2, C1=Chambezi season 1, C2=Chambezi season 2, Nam=Namikonga and Alb=Albert

Supplementary Note 3 Figure 1a: Frequency distribution of mean CBSD root symptoms obtained from phenotyping trials in two seasons, namely 2013 and 2014 at Naliendele and Chambezi.


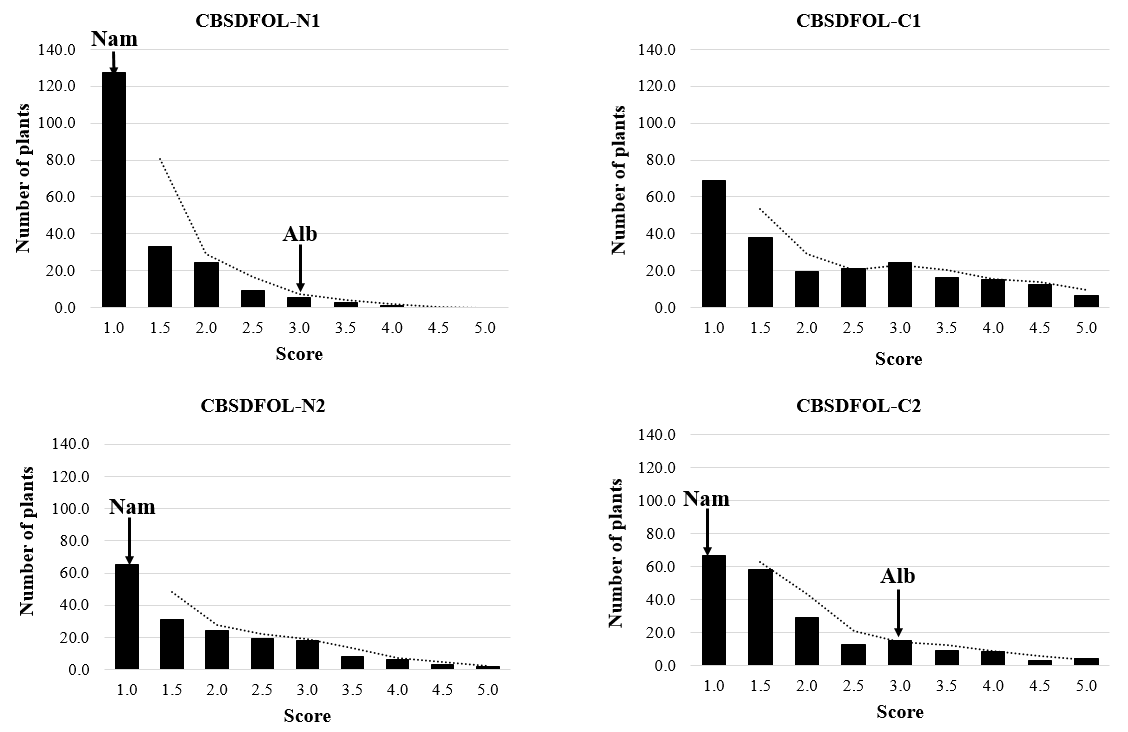


N1=Naliendele season 1, N2=Naliendele season 2, C1=Chambezi season 1, C2=Chambezi season 2, Nam=Namikonga and Alb=Albert

Supplementary Note 3 Figure 1b: Frequency distribution of mean CBSD foliar symptoms obtained from phenotyping trials in two seasons, namely 2013 and 2014 at Naliendele and Chambezi.


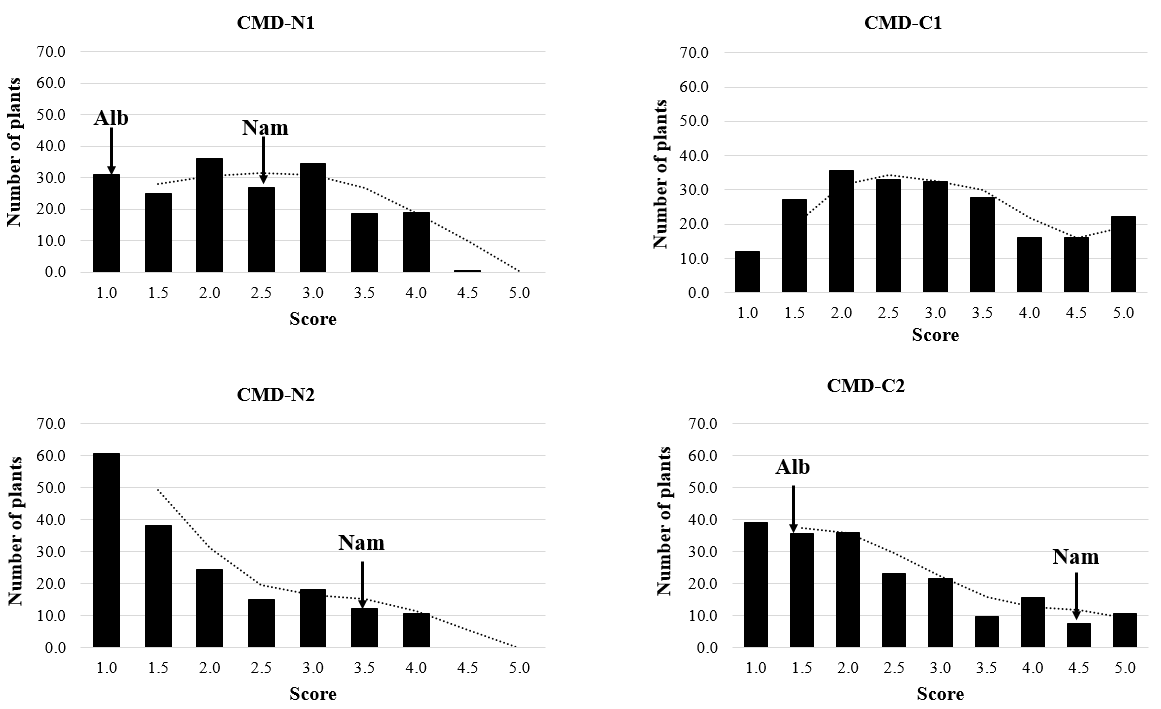


N1=Naliendele season 1, N2=Naliendele season 2, C1=Chambezi season 1, C2=Chambezi season 2, Nam=Namikonga and Alb=Albert

Supplementary Note 3 Figure 1c: Frequency distribution of mean CMD symptoms obtained from phenotyping trials in two seasons, namely 2013 and 2014 at Naliendele and Chambezi.
