# Supplementary material for: QTL associated with resistance to cassava brown streak and cassava mosaic diseases in a bi-parental cross of two Tanzanian farmer varieties, Namikonga and Albert
Source: Theor Appl Genet. 2017 Jul 13;130(10):2069–90. doi: 10.1007/s00122-017-2943-z (PMC5606945; doi:10.1007/s00122-017-2943-z)

**SUPPLEMENTARY NOTE 4:**

**Article title**: QTL associated with resistance to cassava brown streak and cassava mosaic diseases in a bi-parental cross of two Tanzanian farmer-varieties, Namikonga and Albert

**Journal Name**: Theoretical and Applied Genetics

**Author names**: E. A. Masumba, F. Kapinga, G. Mkamilo, S. Kasele, H. Kulembeka, S. Rounsley, J. V. Bredeson, J. B. Lyons, D. S. Rokhsar, E. Kanju, M. S. Katari, A. A. Myburg, N. A. van der Merwe and M. E. Ferguson

**Affiliation and email of corresponding author:** Morag Ferguson, International Institute of Tropical Agriculture (IITA), P.O. Box 30709, Nairobi 00100, Kenya; m.ferguson@cgiar.org

**GACD based LOD profiles showing the QTL regions associated with CBSD root necrosis resistance in ‘Namikonga’.**

**N1**

qCBSDRNFc2Nm


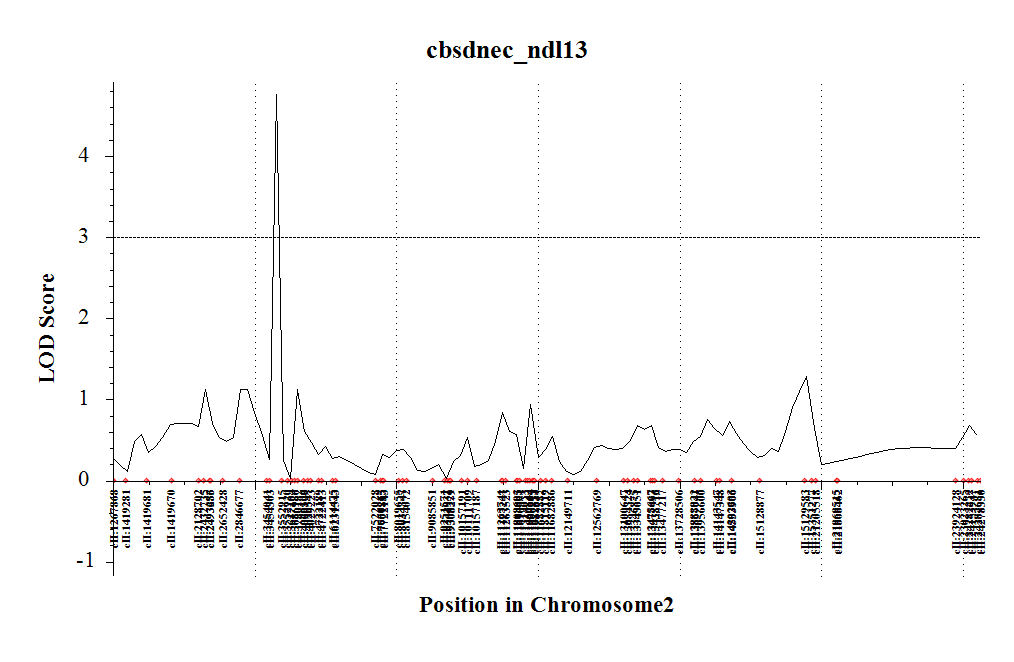


qCBSDRNc7Nm


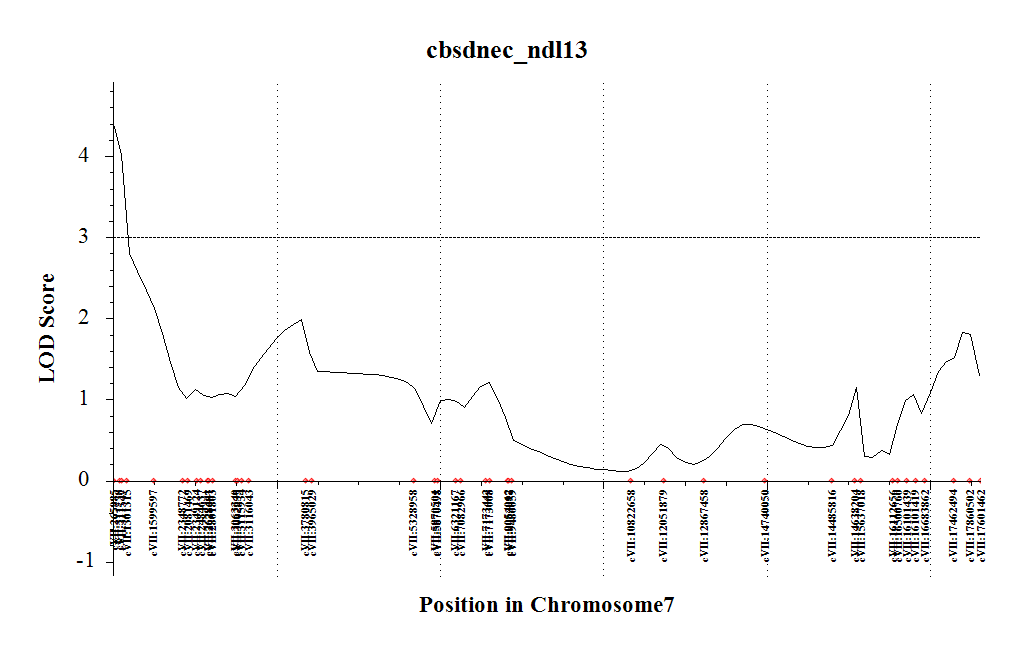


qCBSDRNc10Nm
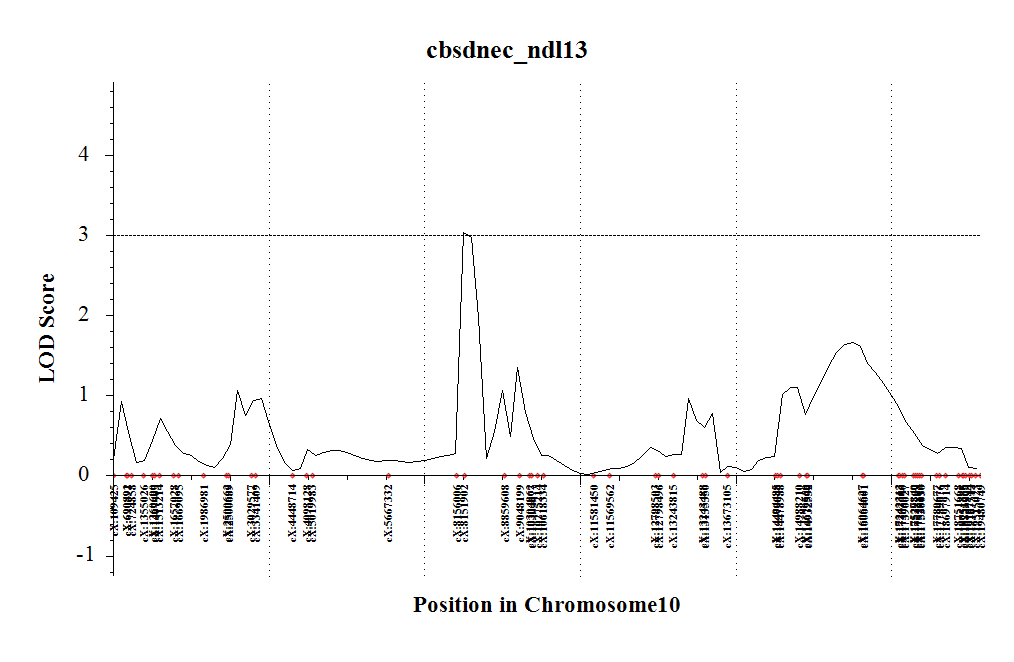


**N2**

qCBSDRNFc2Nm


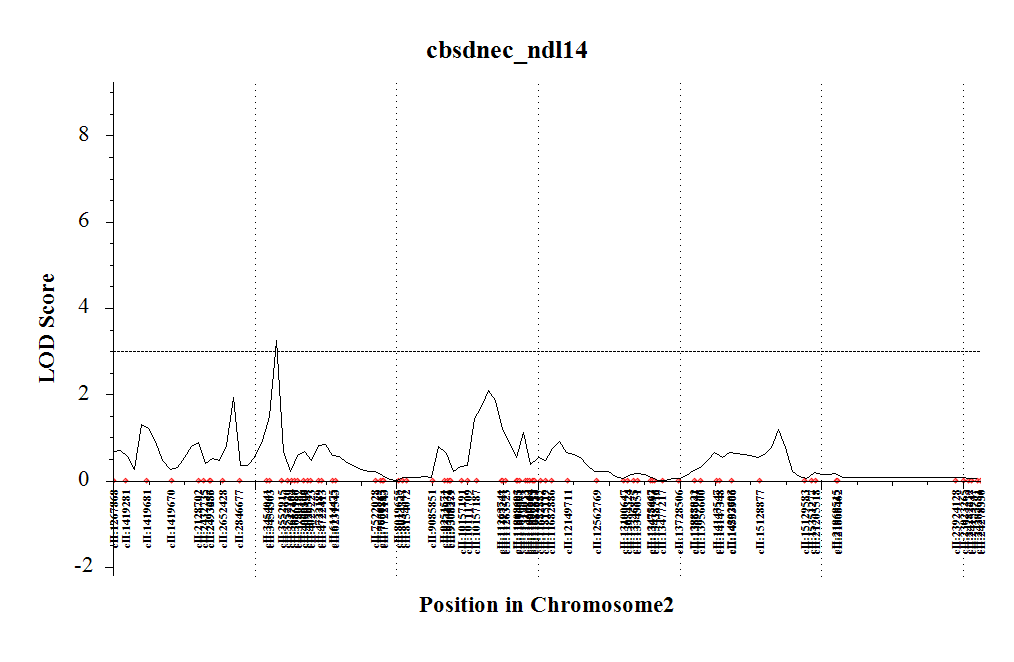


qCBSDRNc4Nm


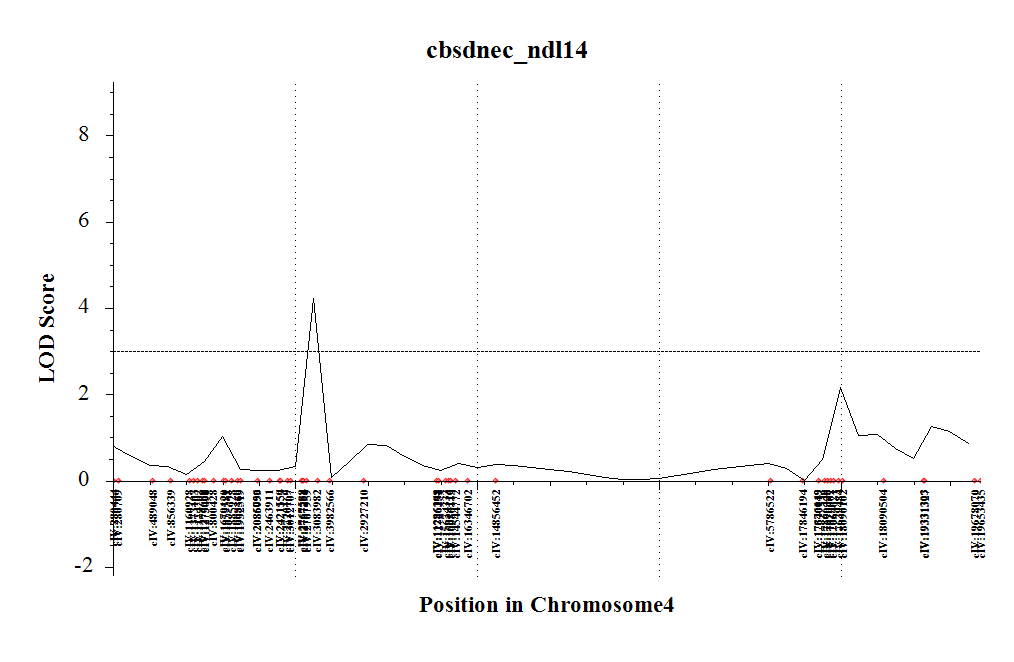


qCBSDRNc12Nm


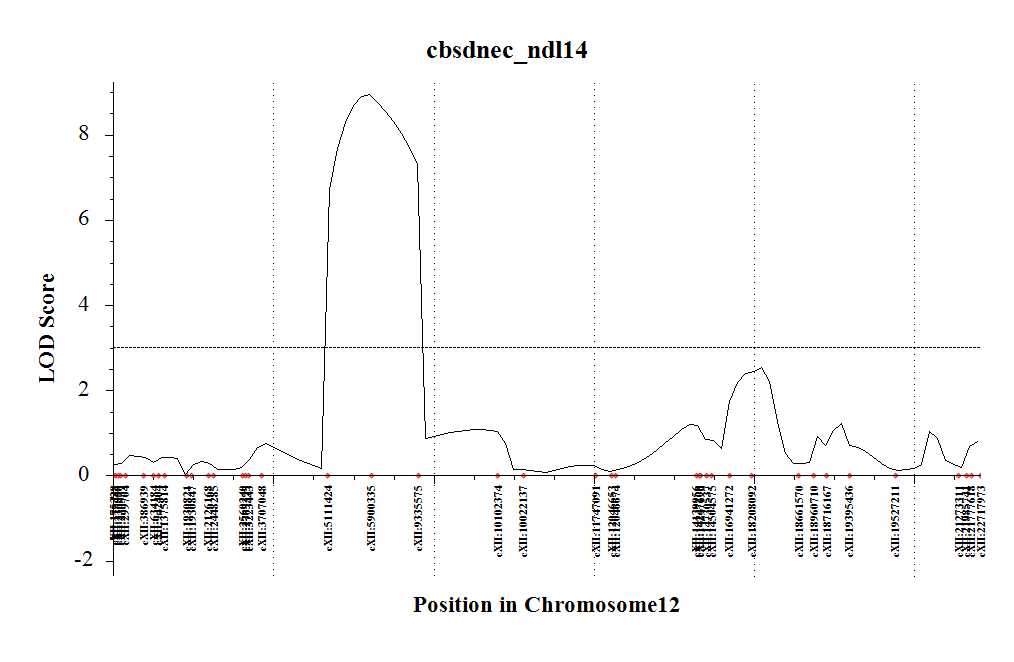


qCBSDRNc18Nm


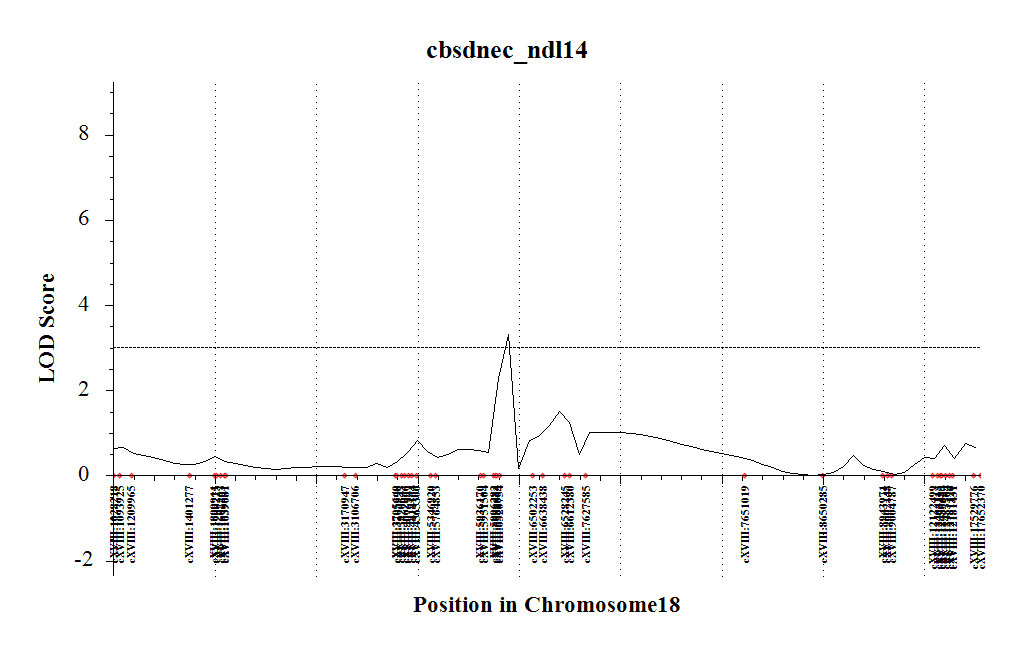


**C1**

CBSDRNc3Nm
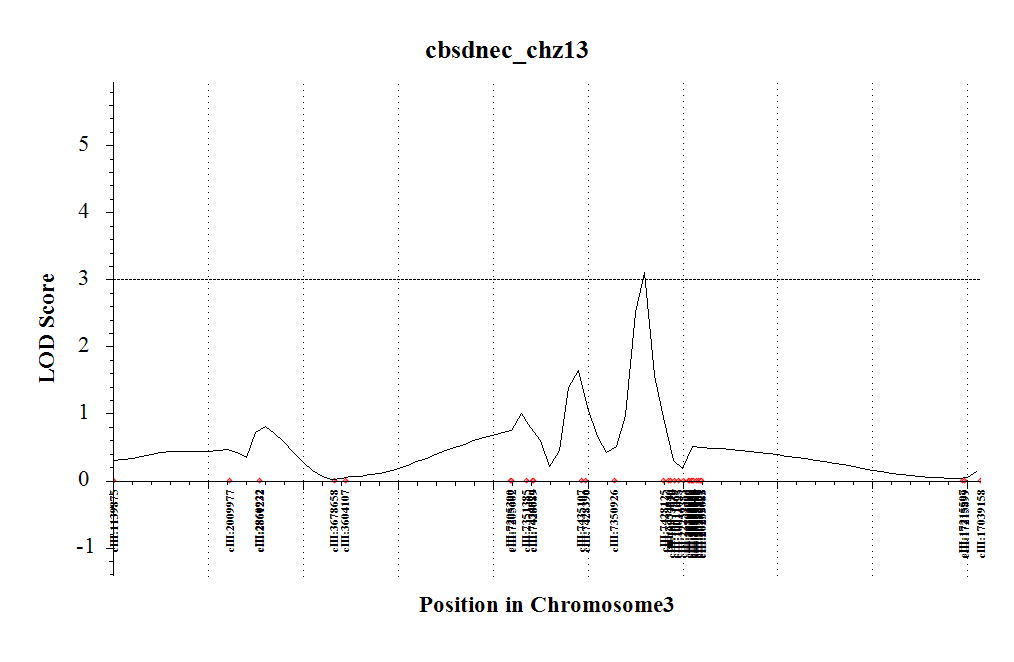


qCBSDRNc5Nm
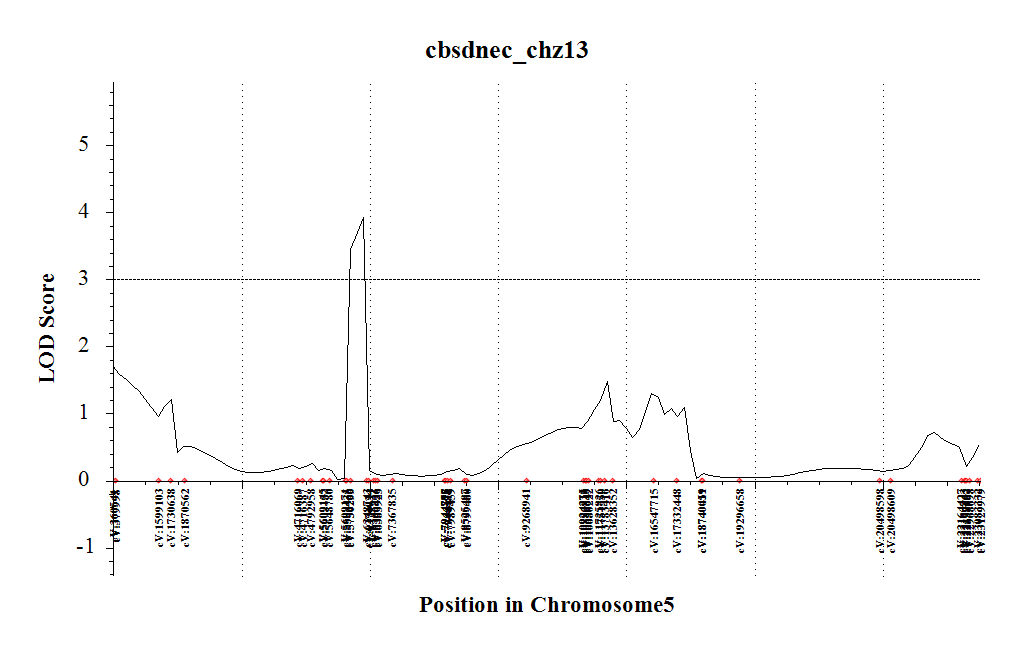


qCBSDRNc11Nm


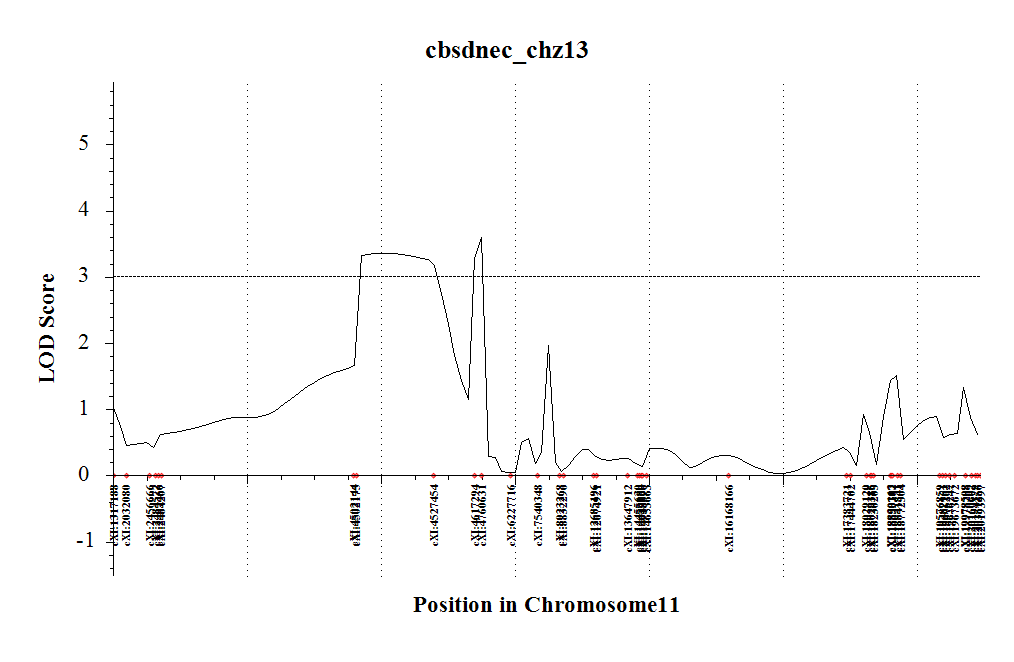


qCBSDRNc15Nm


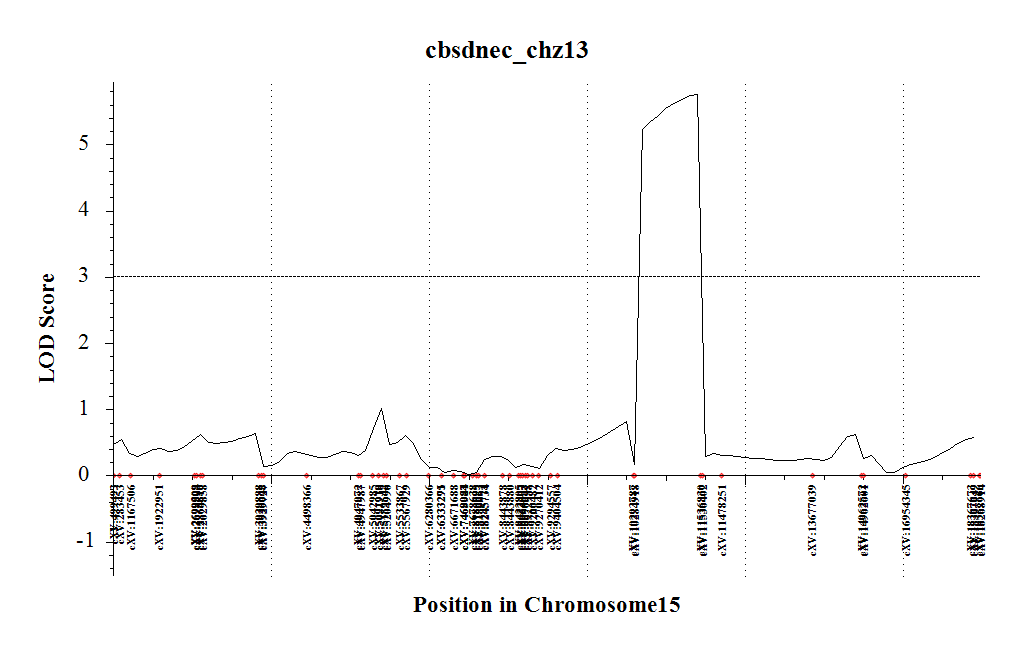


qCBSDRNc16Nm


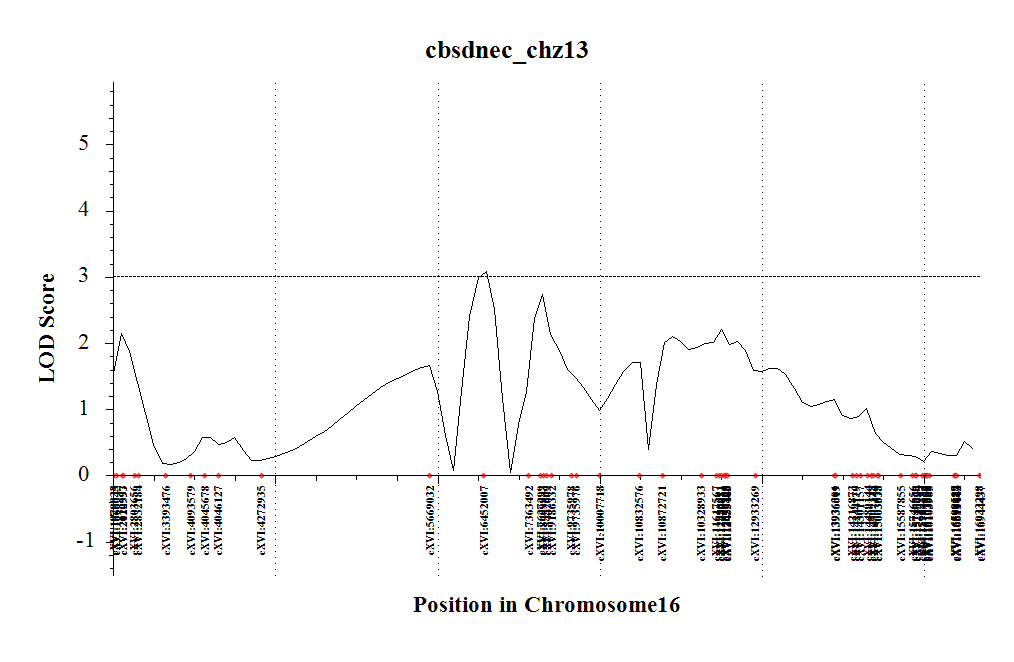


**C2**

qCBSDRNc6Nm
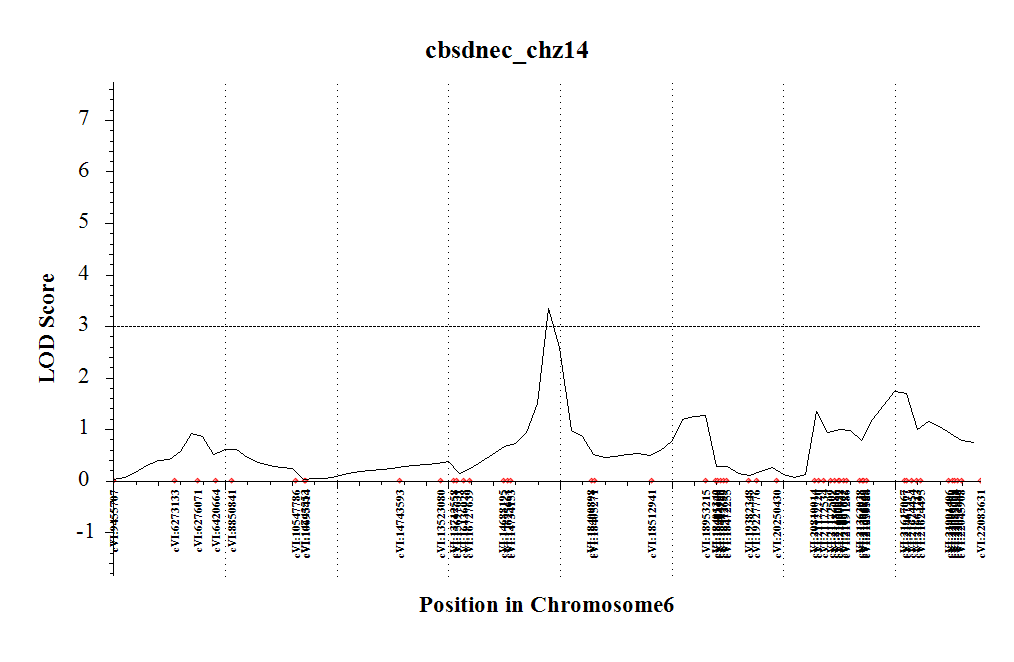


qCBSDRNc11Nm
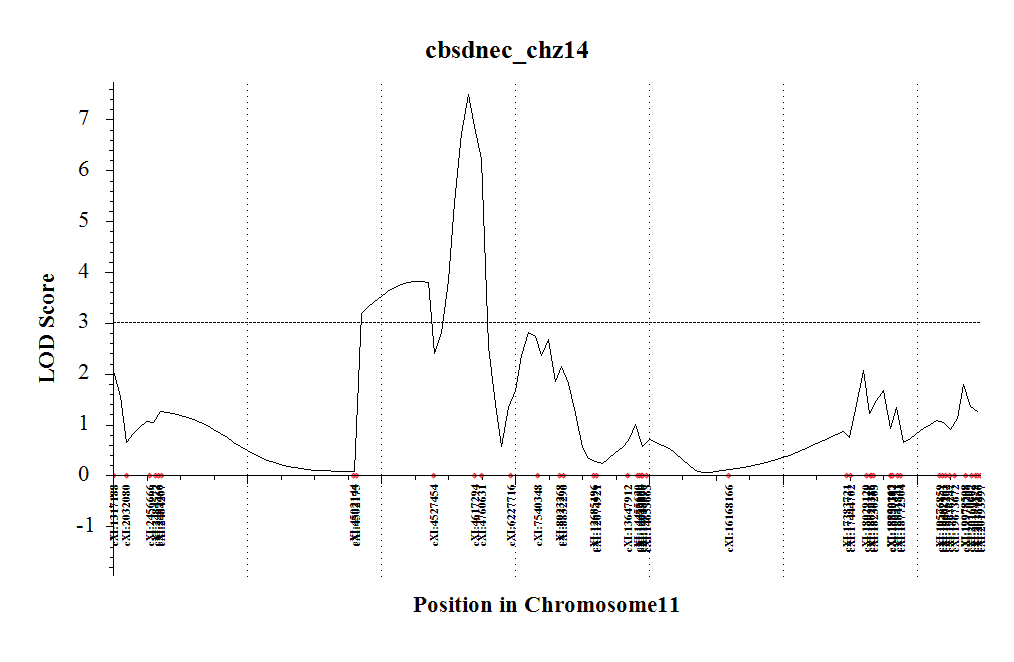


qCBSDRNc16Nm


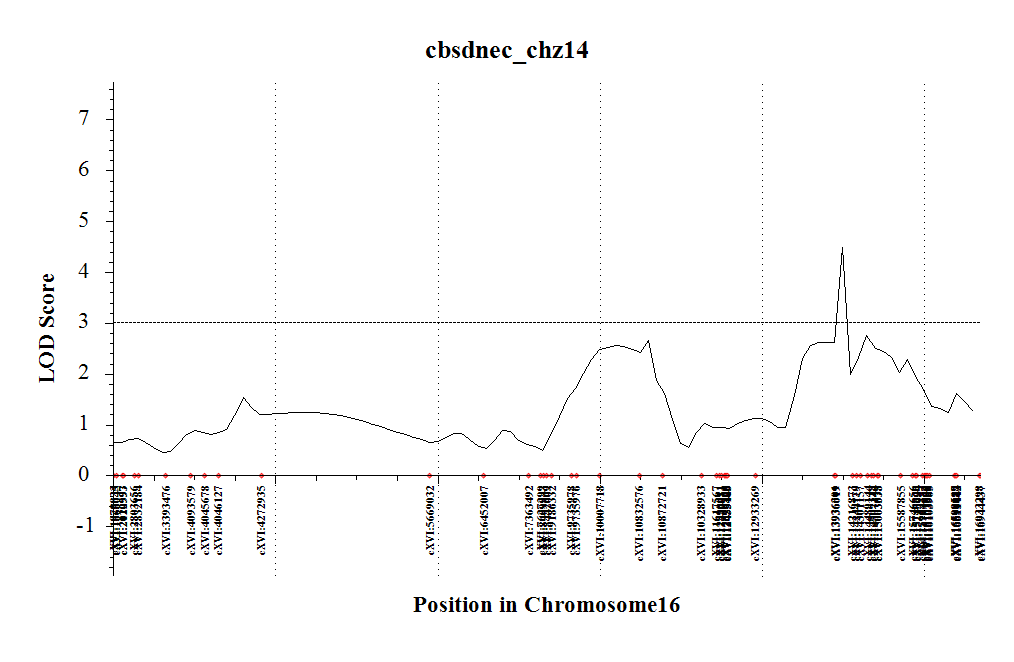


qCBSDRNc18Nm


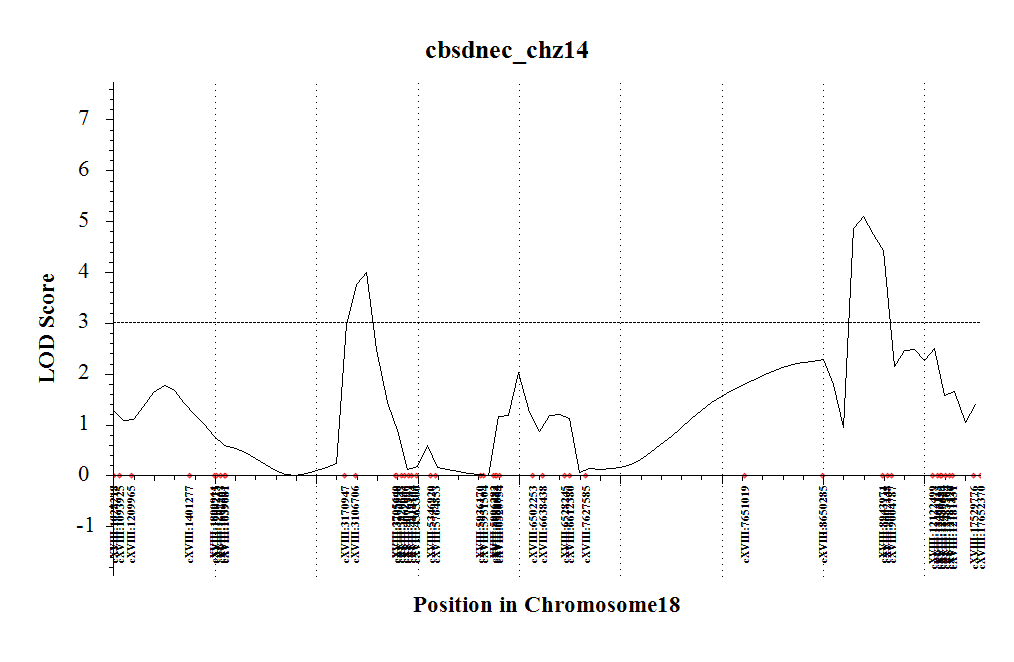

Supplement: Supplementary file 4 — Note 4: GACD based LOD profiles showing the QTL regions associated with CBSD root necrosis resistance in ‘Namikonga’ (DOCX 560 kb) [file 122_2017_2943_MOESM4_ESM.docx]
