# Supplementary material for: QTL associated with resistance to cassava brown streak and cassava mosaic diseases in a bi-parental cross of two Tanzanian farmer varieties, Namikonga and Albert
Source: Theor Appl Genet. 2017 Jul 13;130(10):2069–90. doi: 10.1007/s00122-017-2943-z (PMC5606945; doi:10.1007/s00122-017-2943-z)
Supplement: Supplementary file 5 — Note 5: MapQTL Profiles showing the QTL regions putatively associated with CBSDRN resistance in ‘Namikonga’ based on N1, N2, C1 and C2 phenotyping experiments (DOCX 50 kb) [file 122_2017_2943_MOESM5_ESM.docx]

**SUPPLEMENTARY NOTE 5:**

**Article title**: QTL associated with resistance to cassava brown streak and cassava mosaic diseases in a bi-parental cross of two Tanzanian farmer-varieties, Namikonga and Albert

**Journal Name**: Theoretical and Applied Genetics

**Author names**: E. A. Masumba, F. Kapinga, G. Mkamilo, S. Kasele, H. Kulembeka, S. Rounsley, J. V. Bredeson, J. B. Lyons, D. S. Rokhsar, E. Kanju, M. S. Katari, A. A. Myburg, N. A. van der Merwe and M. E. Ferguson

**Affiliation and email of corresponding author:** Morag Ferguson, International Institute of Tropical Agriculture (IITA), P.O. Box 30709, Nairobi 00100, Kenya; m.ferguson@cgiar.org

MapQTL Profiles showing the QTL regions putatively associated with CBSDRN resistance in ‘Namikonga’ based on N1, N2, C1 and C2 phenotyping experiments.

**N1**

**Chromosome 1**

**N2**

**Chromosome 4**

**C1**

**Chromosome 11**

**Chromosome 5**

**C2**

**Chromosome 11**

**Chromosome 14**

Table 4: Statistics of the traits data obtained by permutation test using MapQTL v6.0 software.

| Trait | Chromosome | QTL name | Detected experiment | LOD QTL Peak | LOD (LG) | LOD (GW) |
| --- | --- | --- | --- | --- | --- | --- |
| CBSD | 11 | qCBSDRNc11Nm | N-1, C-1, C-2 | 5.02 | 2.8 | 4.2 |
|  | 10 | qCBSDRNc10Nm | N-1 | 3.07 | 2.8 | 4.2 |
|  | 14 | qCBSDRNc14Nm | N-1, C-2 | 3.51 | 2.7 | 4.2 |
|  | 4 | qCBSDRNc4Nm | N-2 | 3.76 | 2.9 | 4.4 |
|  | 5 | qCBSDRNc5Nm | C-1 | 3.02 | 2.8 | 4.2 |

Env. = Environment, Chr. = Chromosome, N = Naliendele, C = Chambezi, LOD (QTL) = Highest QTL LOD score, LOD (LG) = Linkage group wise significance; LOD (GW) = Genome wise significance.
