# Supplementary material for: QTL associated with resistance to cassava brown streak and cassava mosaic diseases in a bi-parental cross of two Tanzanian farmer varieties, Namikonga and Albert
Source: Theor Appl Genet. 2017 Jul 13;130(10):2069–90. doi: 10.1007/s00122-017-2943-z (PMC5606945; doi:10.1007/s00122-017-2943-z)
Supplement: Supplementary file 8 — Note 8: MapQTL outputs showing QTL associating with CBSD foliar symptoms resistance in ‘Namikonga’ (DOCX 98 kb) [file 122_2017_2943_MOESM8_ESM.docx]

**SUPPLEMENTARY NOTE 8:**

**Article title**: QTL associated with resistance to cassava brown streak and cassava mosaic diseases in a bi-parental cross of two Tanzanian farmer-varieties, Namikonga and Albert

**Journal Name**: Theoretical and Applied Genetics

**Author names**: E. A. Masumba, F. Kapinga, G. Mkamilo, S. Kasele, H. Kulembeka, S. Rounsley, J. V. Bredeson, J. B. Lyons, D. S. Rokhsar, E. Kanju, M. S. Katari, A. A. Myburg, N. A. van der Merwe and M. E. Ferguson

**Affiliation and email of corresponding author:** Morag Ferguson, International Institute of Tropical Agriculture (IITA), P.O. Box 30709, Nairobi 00100, Kenya; m.ferguson@cgiar.org

MapQTL outputs showing QTLs associating with CBSD foliar symptoms resistance in Namikonga.

**N1 - 3MAP**

**Chromosome 10**

| Position | Locus | LOD | mu_ac{00} | mu_ad{00} | mu_bc{00} | mu_bd{00} | Variance | % Expl. | GIC_1 | GIC_2 | GIC_m |
| --- | --- | --- | --- | --- | --- | --- | --- | --- | --- | --- | --- |
| 0.00 | cX:19480749 | 3.94 | 1.74491 | 2.0296 | 0.475183 | 0.258127 | 0.11288 | 8.4 | 0.012 | 0.986 | 0.499 |
| 1.00 | - | 3.94 | 1.7358 | 2.01091 | 0.482924 | 0.27788 | 0.112872 | 8.4 | 0.013 | 0.958 | 0.485 |
| 2.00 | - | 3.94 | 1.7269 | 1.99269 | 0.490865 | 0.296643 | 0.112869 | 8.4 | 0.013 | 0.943 | 0.478 |
| 3.00 | - | 3.94 | 1.71822 | 1.97493 | 0.499059 | 0.314391 | 0.112869 | 8.4 | 0.014 | 0.942 | 0.478 |
| 4.00 | - | 3.94 | 1.70976 | 1.95761 | 0.507526 | 0.331154 | 0.112873 | 8.4 | 0.014 | 0.954 | 0.484 |
| 5.00 | - | 3.94 | 1.70151 | 1.94068 | 0.516253 | 0.347019 | 0.112881 | 8.3 | 0.015 | 0.979 | 0.497 |

**N1-6MAP**

**Chromosome 14**

**Chromosome 10**

| Position | Locus | LOD | mu_ac{00} | mu_ad{00} | mu_bc{00} | mu_bd{00} | Variance | % Expl. | GIC_1 | GIC_2 | GIC_m |
| --- | --- | --- | --- | --- | --- | --- | --- | --- | --- | --- | --- |
| 44.27 | Peak | 4.97 | 1.31934 | 0.979147 | 1.2172 | 1.63473 | 0.229301 | 10.4 | 0.505 | 0.891 | 0.698 |
| 40.27 | cXIV:6254981 | 4.79 | 1.28849 | 0.98895 | 1.25306 | 1.62221 | 0.230226 | 10 | 0.533 | 0.996 | 0.765 |
| 49.339 | cXIV:5101564 | 4.63 | 1.33033 | 1.01181 | 1.20675 | 1.59699 | 0.231005 | 9.7 | 0.523 | 0.993 | 0.758 |
| 0 | cX:19480749 | 4.17 | 1.76158 | 2.65267 | 0.713585 | -0.06254 | 0.233361 | 8.8 | 0.012 | 0.986 | 0.499 |
| 54.435 | cXIV:4738351 | 4.01 | 1.34607 | 1.04589 | 1.2056 | 1.54729 | 0.234175 | 8.5 | 0.597 | 0.999 | 0.798 |
| 57.078 | cXIV:2176277 | 3.75 | 1.33829 | 1.06317 | 1.20339 | 1.5274 | 0.235541 | 7.9 | 0.657 | 0.997 | 0.827 |
| 26.584 | cXIV:8274328 | 3.68 | 1.25794 | 1.09527 | 1.26985 | 1.51971 | 0.235887 | 7.8 | 0.922 | 0.936 | 0.929 |
| 33.065 | cXIV:6817485 | 3.53 | 1.26743 | 1.03817 | 1.30537 | 1.53689 | 0.236694 | 7.5 | 0.677 | 0.999 | 0.838 |
| 62.773 | cXIV:601892 | 3.46 | 1.32654 | 1.12158 | 1.19432 | 1.49223 | 0.237059 | 7.3 | 0.851 | 0.888 | 0.87 |

**N1-9MAP**

**Chromosome 14**

| Position | Locus | LOD | mu_ac{00} | mu_ad{00} | mu_bc{00} | mu_bd{00} | Variance | % Expl. | GIC_1 | GIC_2 | GIC_m |
| --- | --- | --- | --- | --- | --- | --- | --- | --- | --- | --- | --- |
| 44.27 | Peak | 3.1 | 1.82264 | 1.14959 | 1.43415 | 1.59231 | 0.440313 | 7 | 0.505 | 0.891 | 0.698 |
| 40.27 | cXIV:6254981 | 2.9 | 1.77819 | 1.17107 | 1.43663 | 1.59506 | 0.442389 | 6.5 | 0.533 | 0.996 | 0.765 |
| 49.339 | cXIV:5101564 | 2.85 | 1.80176 | 1.19349 | 1.4474 | 1.58388 | 0.442834 | 6.4 | 0.523 | 0.993 | 0.758 |
| 33.065 | cXIV:6817485 | 2.81 | 1.75013 | 1.21026 | 1.41113 | 1.62627 | 0.443219 | 6.4 | 0.677 | 0.999 | 0.838 |

**N2-3MAP**

**Chromosome 12**

| Position | Locus | LOD | mu_ac{00} | mu_ad{00} | mu_bc{00} | mu_bd{00} | Variance | % Expl. | GIC_1 | GIC_2 | GIC_m |
| --- | --- | --- | --- | --- | --- | --- | --- | --- | --- | --- | --- |
| 9.167 | Peak | 3.07 | 1.45771 | 2.12258 | 1.66434 | 1.70023 | 0.502777 | 7.5 | 0.694 | 0.939 | 0.817 |
| 7.167 | cXII:2448285 | 3.02 | 1.46618 | 2.11513 | 1.6724 | 1.69368 | 0.503322 | 7.4 | 0.701 | 0.995 | 0.848 |
| 13.334 | cXII:3352898 | 2.83 | 1.47924 | 2.09343 | 1.65209 | 1.70973 | 0.505848 | 6.9 | 0.723 | 0.996 | 0.86 |

**N2-6MAP**

- No significant QTL

**N2-9MAP**

**Chromosome 16**

| Position | Locus | LOD | mu_ac{00} | mu_ad{00} | mu_bc{00} | mu_bd{00} | Variance | % Expl. | GIC_1 | GIC_2 | GIC_m |
| --- | --- | --- | --- | --- | --- | --- | --- | --- | --- | --- | --- |
| 14.728 | Peak | 3.05 | 2.10393 | 2.04861 | 1.69671 | 2.85706 | 1.17301 | 7.6 | 0.282 | 0.884 | 0.583 |
| 18.78 | cXVI:4272937 | 2.96 | 2.09505 | 2.07193 | 1.73518 | 2.80333 | 1.17598 | 7.4 | 0.331 | 0.992 | 0.662 |
|  |  |  |  |  |  |  |  |  |  |  |  |

**C1-3MAP**

- No significant QTL

**C1-6MAP**

**Chromosome 1**

**Chromosome 13**

| Position | Locus | LOD | mu_ac{00} | mu_ad{00} | mu_bc{00} | mu_bd{00} | Variance | % Expl. | GIC_1 | GIC_2 | GIC_m |
| --- | --- | --- | --- | --- | --- | --- | --- | --- | --- | --- | --- |
| 15.00 | Peak | 3.42 | 3.03766 | 3.02386 | 3.73061 | 3.35715 | 0.82048 | 6.8 | 0.812 | 0.768 | 0.79 |
| 19.52 | cXIII:16309346 | 3.34 | 3.06603 | 3.0277 | 3.67078 | 3.35977 | 0.82172 | 6.7 | 0.944 | 0.92 | 0.932 |
| 134.27 | cI:24804445 | 3.13 | 3.12792 | 3.74258 | 3.07568 | 3.17227 | 0.825431 | 6.3 | 0.597 | 0.947 | 0.772 |
| 22.89 | cXIII:17105456 | 3.09 | 3.09615 | 3.01833 | 3.63238 | 3.37121 | 0.826018 | 6.2 | 0.992 | 0.798 | 0.895 |

**C1-9MAP**

**Chromosome 5**

**Chromosome 12**

| Position | Locus | LOD | mu_ac{00} | mu_ad{00} | mu_bc{00} | mu_bd{00} | Variance | % Expl. | GIC_1 | GIC_2 | GIC_m |
| --- | --- | --- | --- | --- | --- | --- | --- | --- | --- | --- | --- |
| 33.722 | Peak | 12.35 | 2.26798 | 1.60559 | 2.30517 | 1.46143 | 0.477996 | 22.6 | 0.654 | 0.959 | 0.806 |
| 34.699 | cXII:9335575 | 12.26 | 2.25947 | 1.60079 | 2.29086 | 1.47428 | 0.478964 | 22.4 | 0.648 | 0.996 | 0.822 |
| 27.722 | cXII:6221132 | 9.55 | 2.24648 | 1.68856 | 2.26446 | 1.51903 | 0.506568 | 18 | 0.738 | 0.995 | 0.867 |
| 43.864 | cXII:10102374 | 8.65 | 2.17761 | 1.67072 | 2.25561 | 1.49403 | 0.516203 | 16.4 | 0.719 | 0.972 | 0.846 |
| 21.208 | cXII:4057645 | 8.07 | 2.23783 | 1.67506 | 2.19158 | 1.52235 | 0.522439 | 15.4 | 0.938 | 0.947 | 0.943 |
| 52.823 | cXII:9773230 | 6.01 | 2.12232 | 1.68434 | 2.25987 | 1.55615 | 0.545239 | 11.7 | 0.992 | 0.838 | 0.915 |
| 13.334 | cXII:3352898 | 4.95 | 2.16244 | 1.77838 | 2.13018 | 1.59294 | 0.557306 | 9.8 | 0.723 | 0.996 | 0.86 |
| 57.188 | cXII:11747091 | 4.17 | 2.03595 | 1.73936 | 2.22132 | 1.6412 | 0.566393 | 8.3 | 0.959 | 0.95 | 0.954 |
| 67.528 | cXII:18208092 | 3.62 | 2.05412 | 1.70289 | 2.25913 | 1.65614 | 0.572927 | 7.2 | 0.977 | 0.711 | 0.844 |
| 79.758 | cV:13710209 | 3.39 | 2.30843 | 1.62049 | 1.9214 | 1.80069 | 0.575704 | 6.8 | 0.995 | 0.656 | 0.825 |

**C2-3MAP**

- No significant QTL

**C2-6MAP**

- No significant QTL

**C2-9MAP**

**Chromosome 16,**

**Chromosome 13**

**Chromosome 8**

| Position | Locus | LOD | mu_ac{00} | mu_ad{00} | mu_bc{00} | mu_bd{00} | Variance | % Expl. | GIC_1 | GIC_2 | GIC_m |
| --- | --- | --- | --- | --- | --- | --- | --- | --- | --- | --- | --- |
| 49.825 | cVIII:15126728 | 4.91 | 2.25289 | 2.35299 | 3.08924 | 2.93291 | 1.04935 | 10.3 | 0.947 | 0.901 | 0.924 |
| 45.313 | cVIII:17792779 | 3.66 | 2.3663 | 2.38695 | 3.04866 | 2.89675 | 1.0788 | 7.8 | 0.997 | 0.762 | 0.88 |
| 56.46 | cVIII:11658622 | 3.46 | 2.29106 | 2.42639 | 2.97819 | 2.92104 | 1.08368 | 7.4 | 0.968 | 0.614 | 0.791 |
| 53.192 | cXVI:9735964 | 3.36 | 2.52213 | 2.37404 | 2.66607 | 3.19426 | 1.08609 | 7.2 | 0.974 | 0.993 | 0.983 |
| 22.89 | cXIII:17105456 | 3.2 | 2.48218 | 2.33111 | 2.96213 | 2.96214 | 1.0899 | 6.8 | 0.992 | 0.798 | 0.895 |
| 47.158 | cXVI:11802348 | 3.01 | 2.55741 | 2.29716 | 2.65373 | 3.15242 | 1.0944 | 6.5 | 0.975 | 0.813 | 0.894 |
