# Supplementary material for: QTL associated with resistance to cassava brown streak and cassava mosaic diseases in a bi-parental cross of two Tanzanian farmer varieties, Namikonga and Albert
Source: Theor Appl Genet. 2017 Jul 13;130(10):2069–90. doi: 10.1007/s00122-017-2943-z (PMC5606945; doi:10.1007/s00122-017-2943-z)
Supplement: Supplementary file 9 — Note 9: MapQTL Profiles showing QTL putatively associated with CMD resistance in ‘Albert’ (DOCX 86 kb) [file 122_2017_2943_MOESM9_ESM.docx]

**SUPPLEMENTARY NOTE 9:**

**Article title**: QTL associated with resistance to cassava brown streak and cassava mosaic diseases in a bi-parental cross of two Tanzanian farmer-varieties, Namikonga and Albert

**Journal Name**: Theoretical and Applied Genetics

**Author names**: E. A. Masumba, F. Kapinga, G. Mkamilo, S. Kasele, H. Kulembeka, S. Rounsley, J. V. Bredeson, J. B. Lyons, D. S. Rokhsar, E. Kanju, M. S. Katari, A. A. Myburg, N. A. van der Merwe and M. E. Ferguson

**Affiliation and email of corresponding author:** Morag Ferguson, International Institute of Tropical Agriculture (IITA), P.O. Box 30709, Nairobi 00100, Kenya; m.ferguson@cgiar.org

MapQTL Profiles showing QTLs putatively associated with CMD resistance in Albert.

**N1**


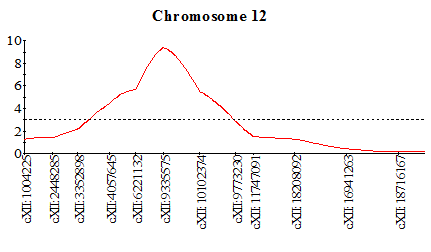


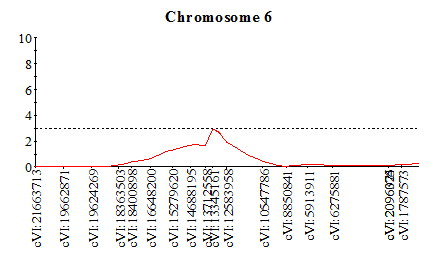


**N2**


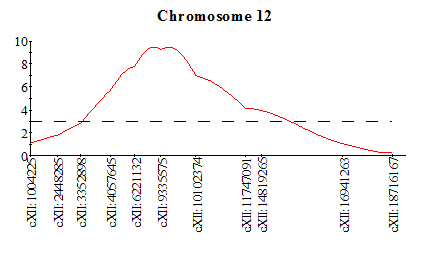


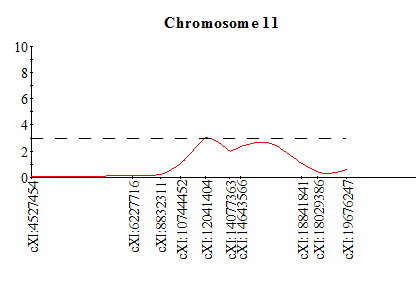


**C1**


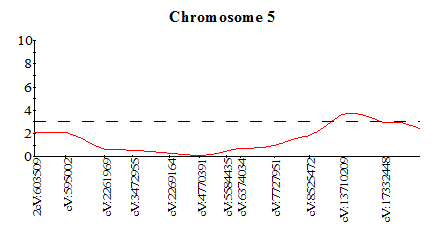


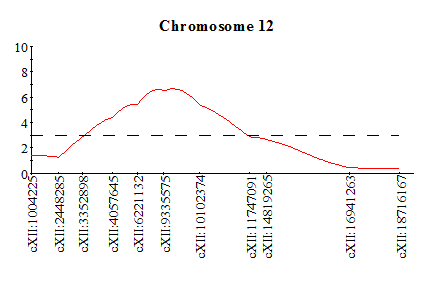


**C2**


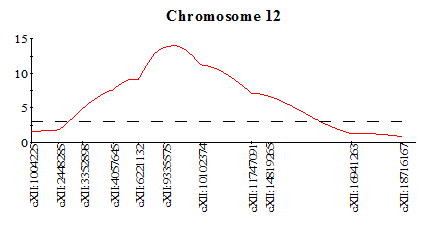


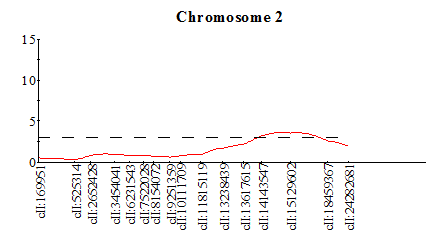


Table 4: Statistics of the traits data obtained by permutation test using MapQTL v6.0 software.

| Trait | Chromosome | QTL name | Detected experiment | LOD QTL Peak | LOD (LG) | LOD (GW) |
| --- | --- | --- | --- | --- | --- | --- |
| CMD | 12 | qCMDc12 | N-1, N-2, C-1, C-2 | 14.03 | 2.8 | 4.1 |
|  | 6 | qCMDc6 | N-1 | 2.94 | 3.0 | 4.1 |
|  | 11 | qCMDc11 | N-2 | 3.08 | 2.8 | 4.2 |
|  | 5 | qCMDc5 | C-1 | 3.74 | 2.9 | 4.2 |

Env. = Environment, Chr. = Chromosome, N = Naliendele, C = Chambezi, LOD (QTL) = Highest QTL LOD score, LOD (LG) = Linkage group wise significance; LOD (GW) = Genome wise significance.
