# Supplementary material for: QTL associated with resistance to cassava brown streak and cassava mosaic diseases in a bi-parental cross of two Tanzanian farmer varieties, Namikonga and Albert
Source: Theor Appl Genet. 2017 Jul 13;130(10):2069–90. doi: 10.1007/s00122-017-2943-z (PMC5606945; doi:10.1007/s00122-017-2943-z)

**SUPPLEMENTARY NOTE 10:**

**Article title**: QTL associated with resistance to cassava brown streak and cassava mosaic diseases in a bi-parental cross of two Tanzanian farmer-varieties, Namikonga and Albert

**Journal Name**: Theoretical and Applied Genetics

**Author names**: E. A. Masumba, F. Kapinga, G. Mkamilo, S. Kasele, H. Kulembeka, S. Rounsley, J. V. Bredeson, J. B. Lyons, D. S. Rokhsar, E. Kanju, M. S. Katari, A. A. Myburg, N. A. van der Merwe and M. E. Ferguson

**Affiliation and email of corresponding author:** Morag Ferguson, International Institute of Tropical Agriculture (IITA), P.O. Box 30709, Nairobi 00100, Kenya; m.ferguson@cgiar.org

GACD LOD profiles showing the genomic regions in Namikonga-Albert F1 individuals that are associated with CMD resistance.

**C1**

qCMDc2A


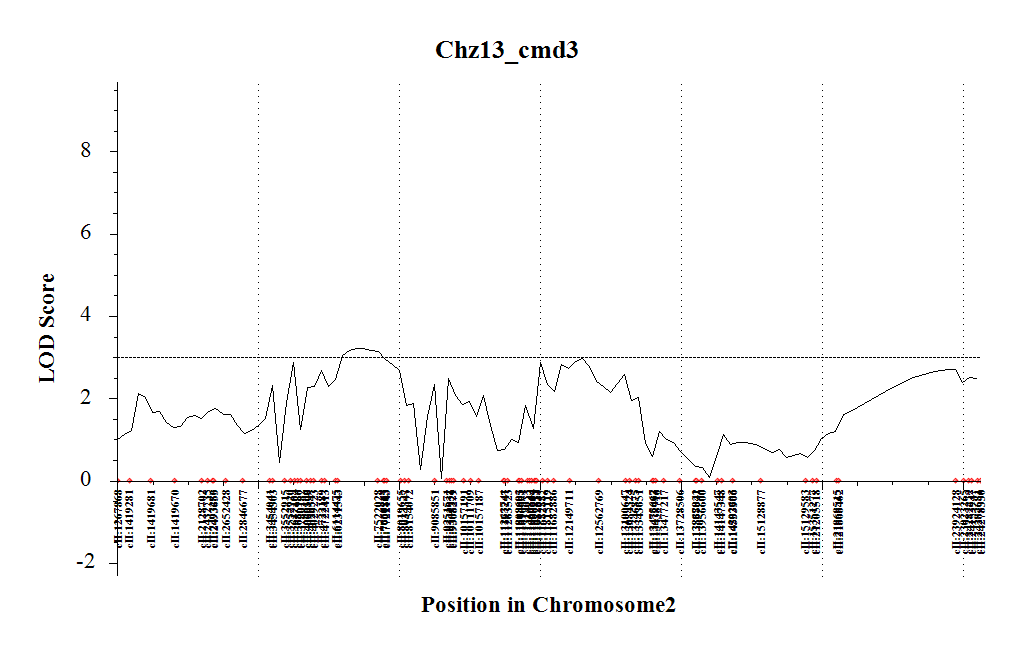


qCMDc4A


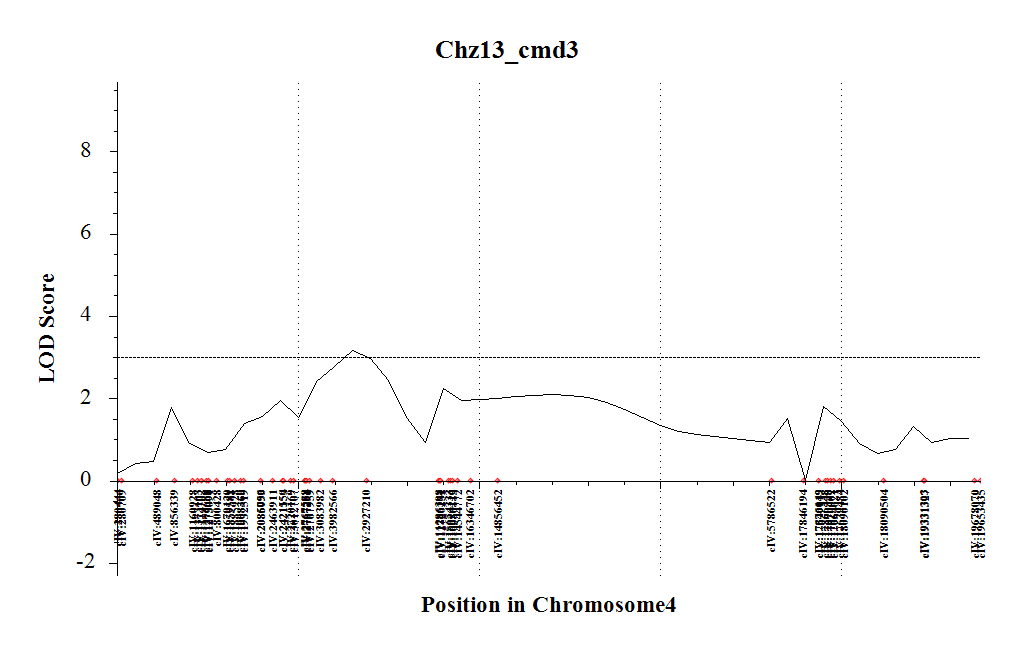


qCMDc10A


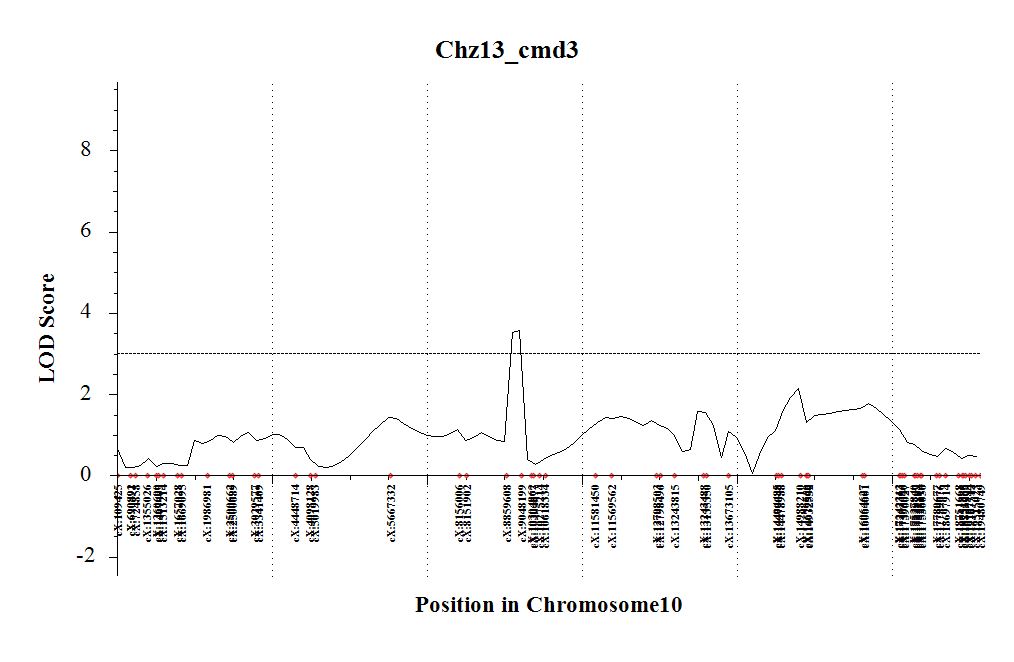


qCMDc12A


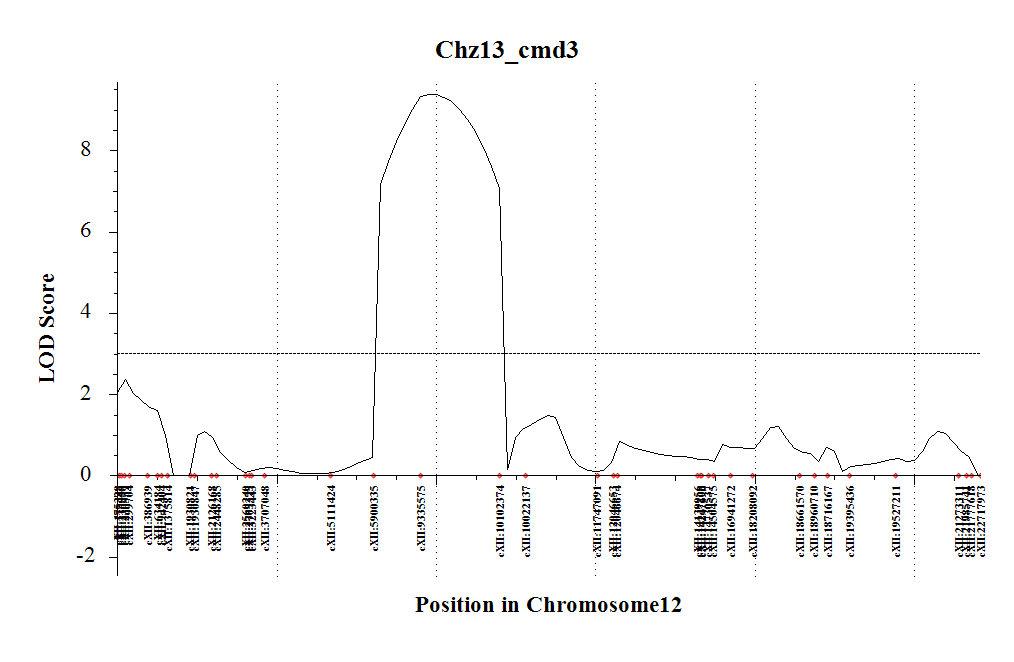


qCMDc17A


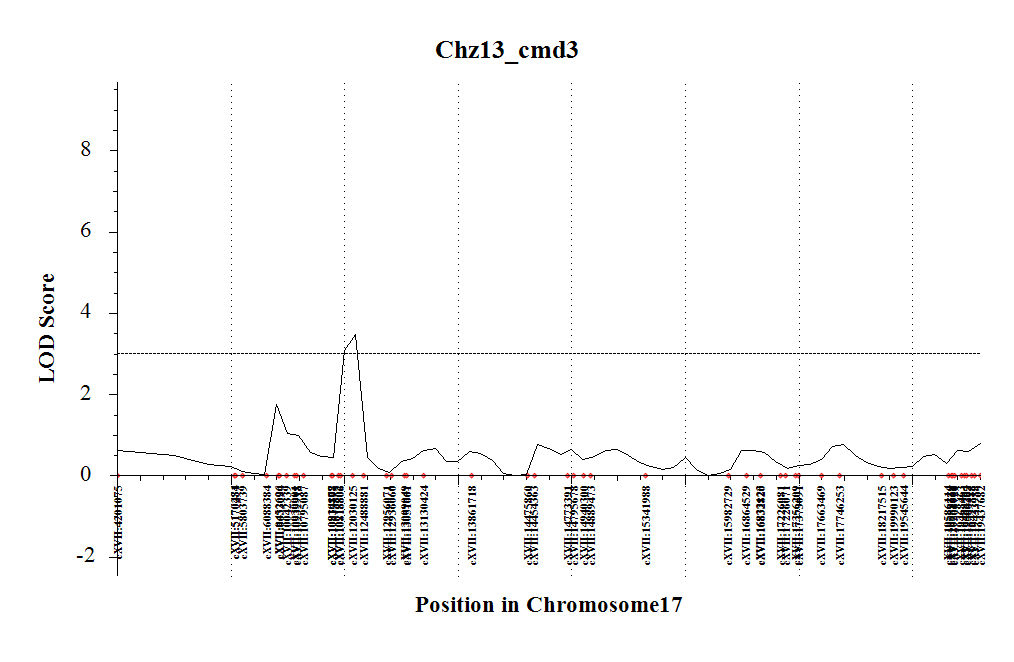


**C2**

qCMDc1A


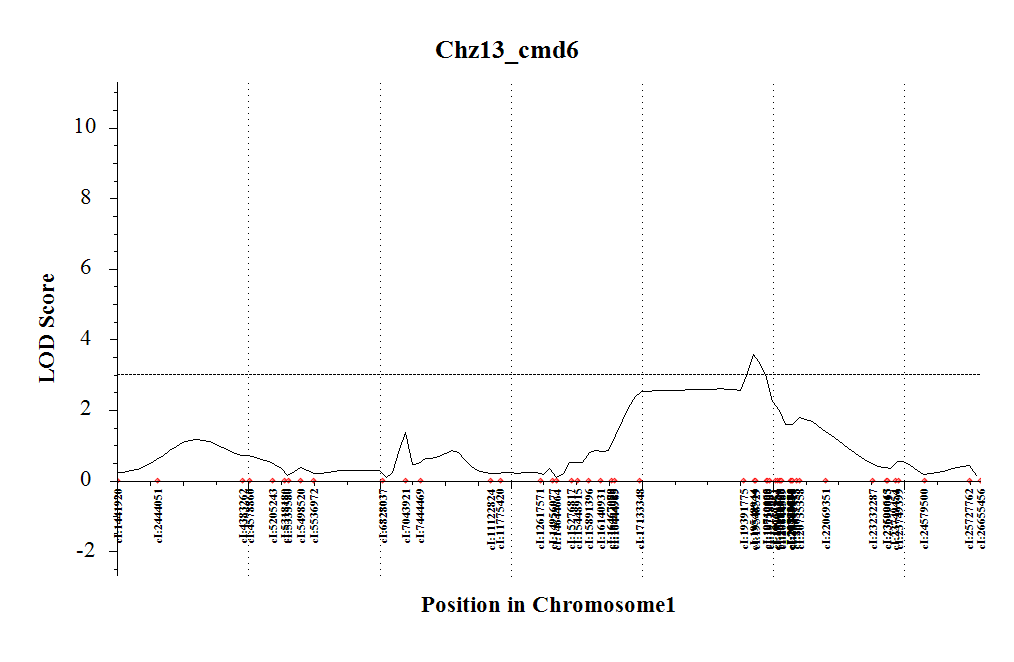


qCMDc3A


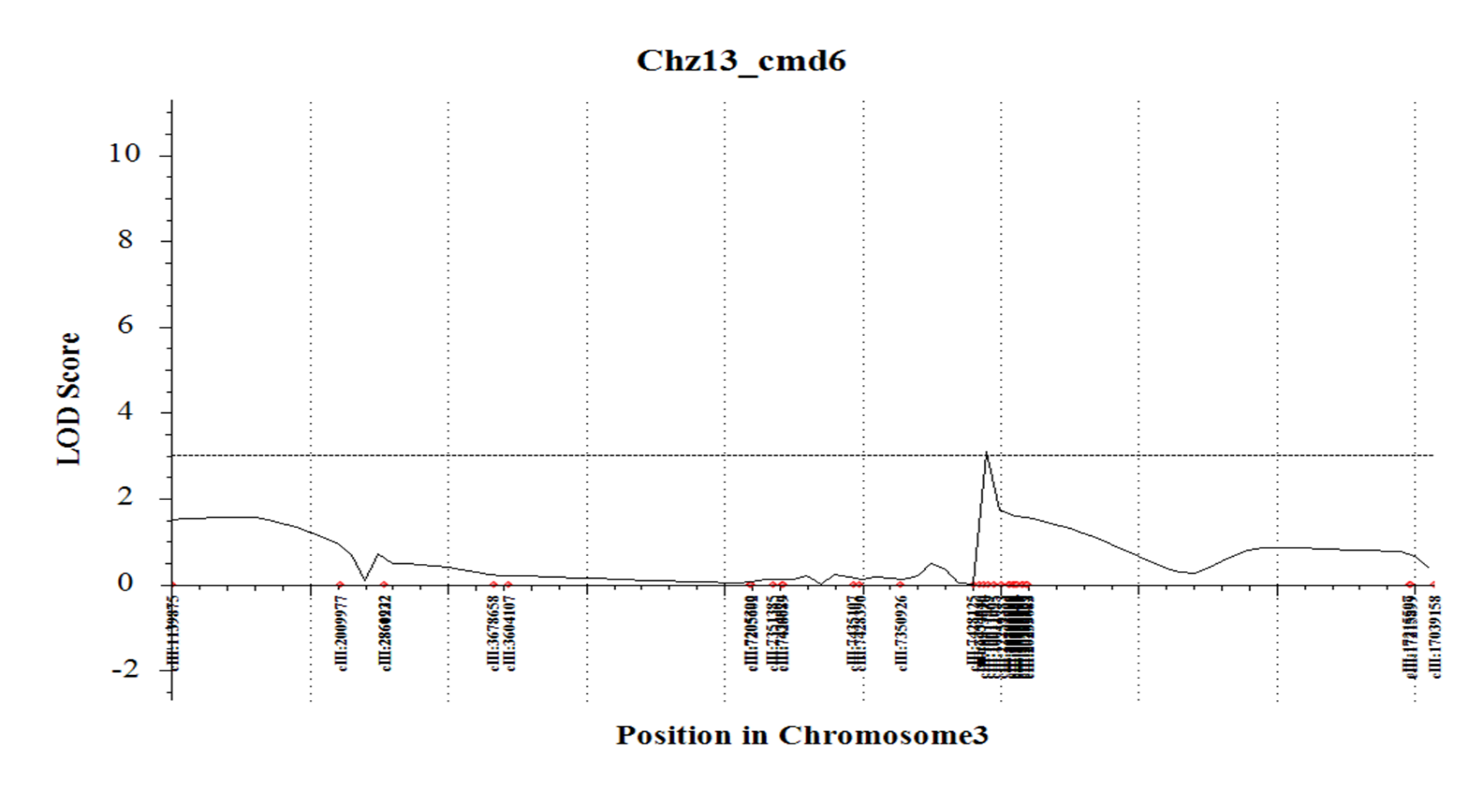


qCMDc5A


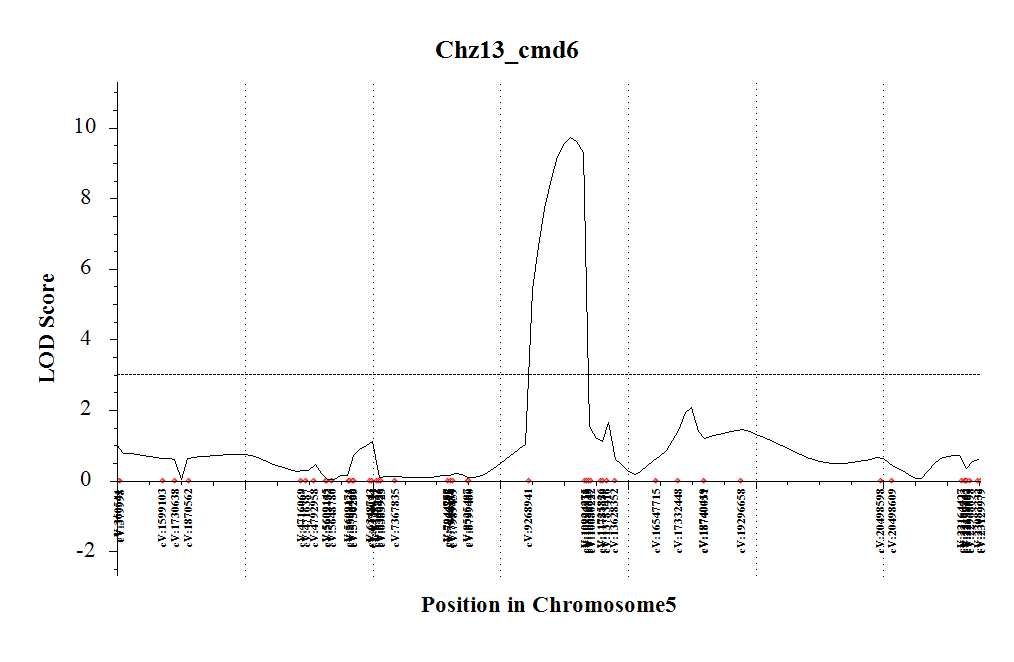


qCMDc12A


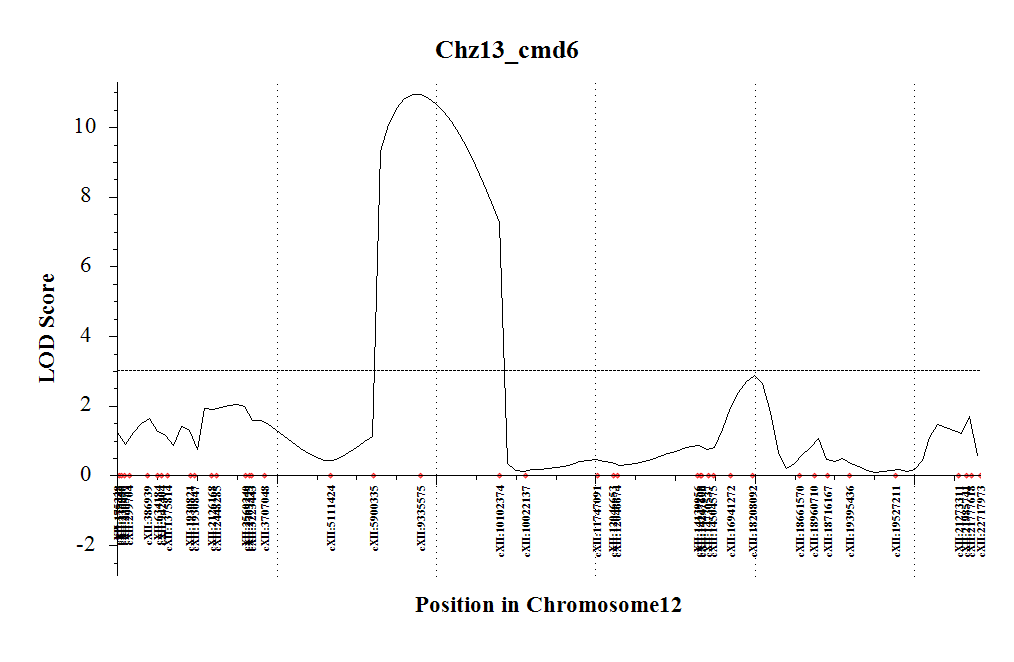


qCMDc16A


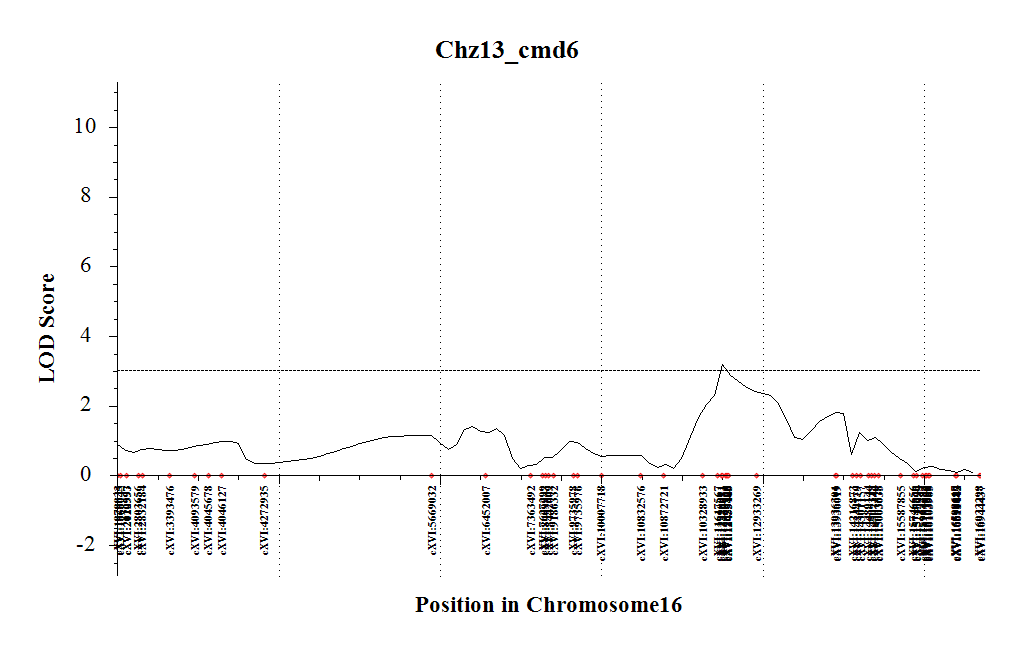


qCMDc10A


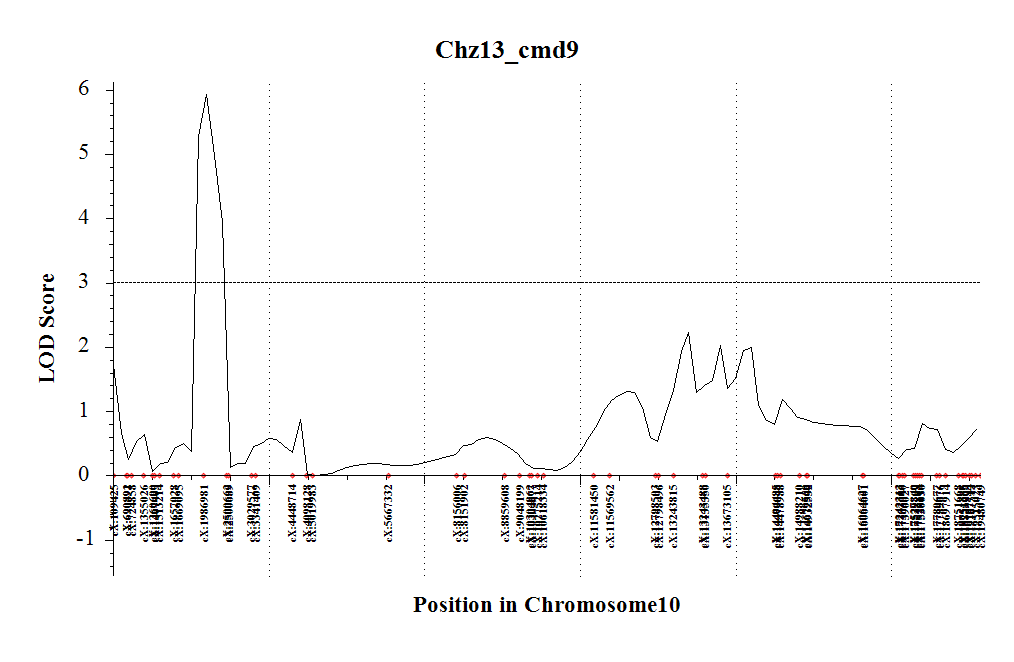


qCMDc18A


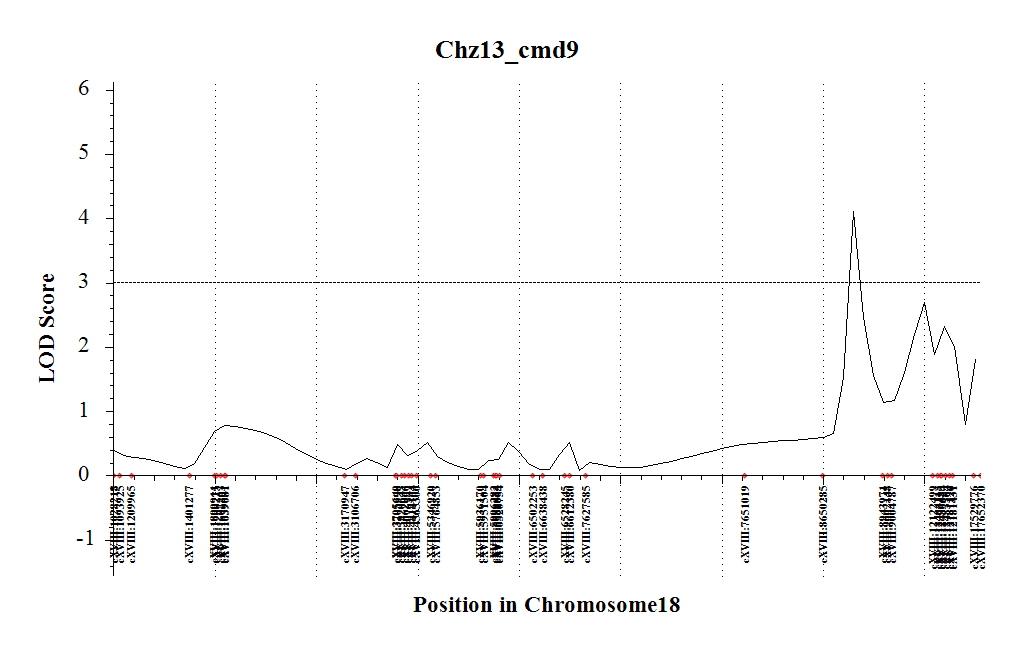


**N1**

qCMDc5A


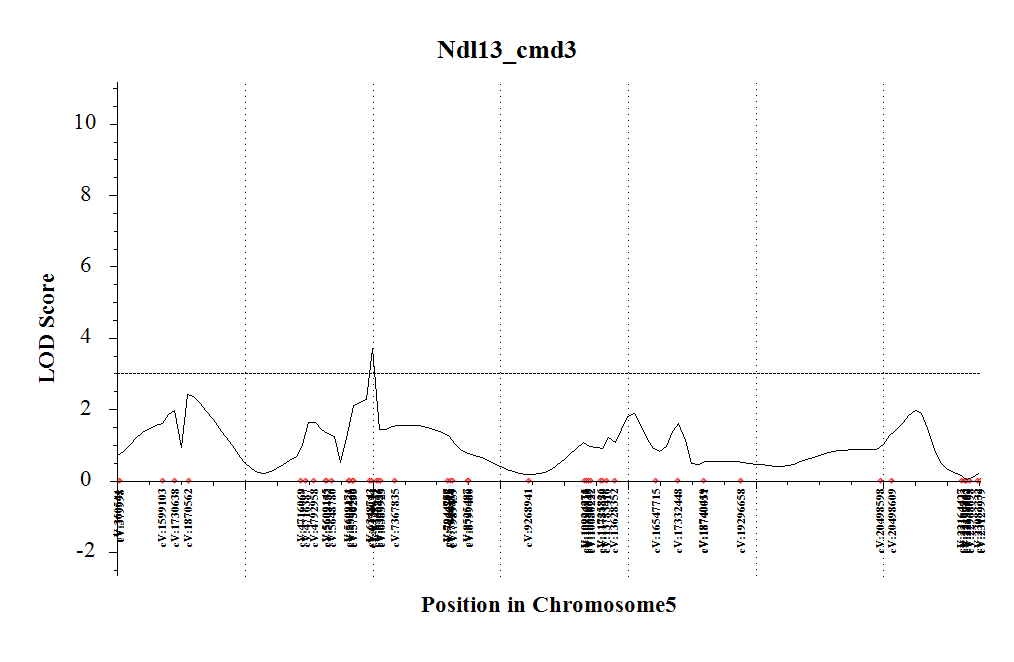


qCMDc12A


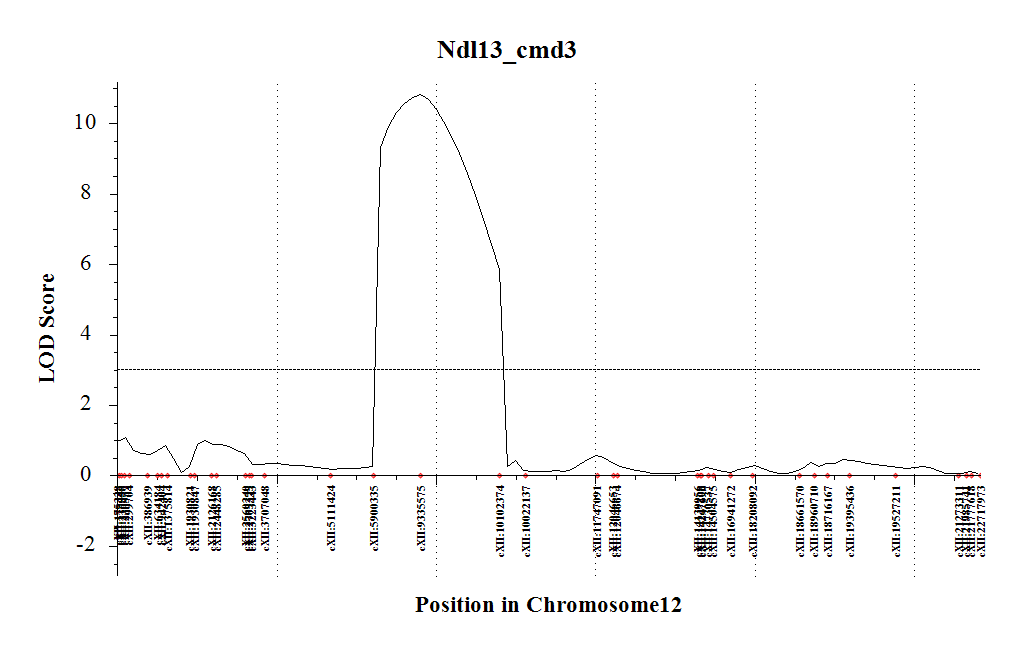


qCMDc12A


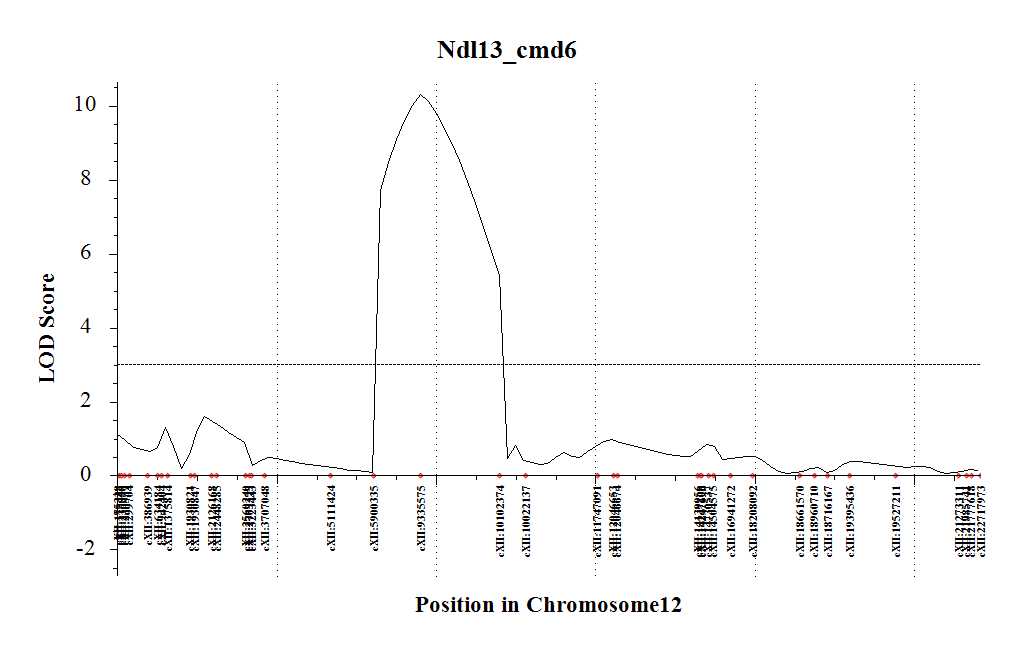


qCMDc6A


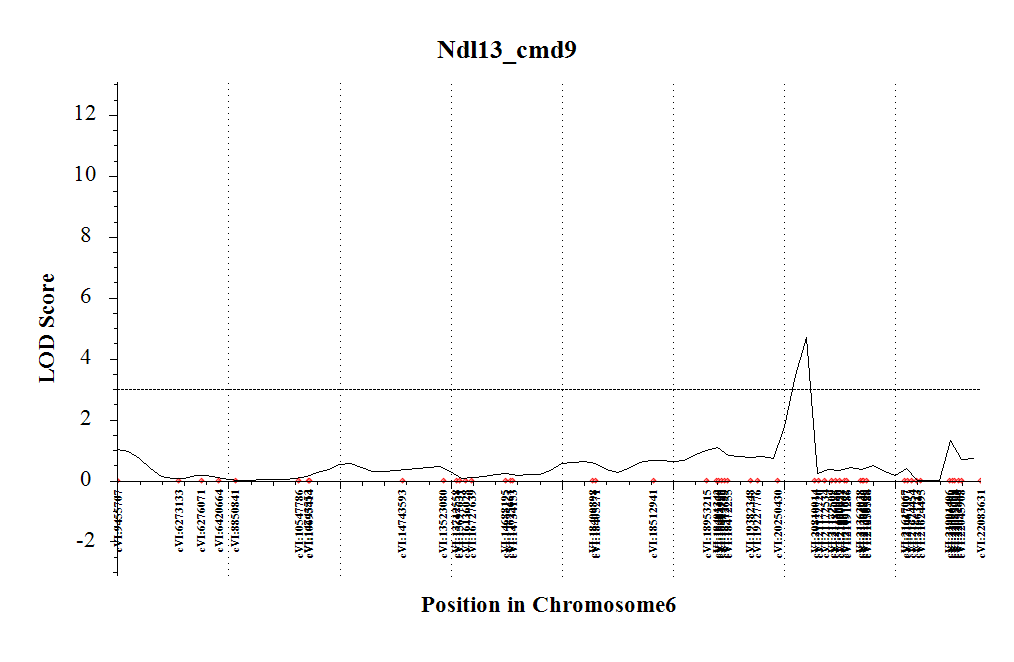


qCMDc12A


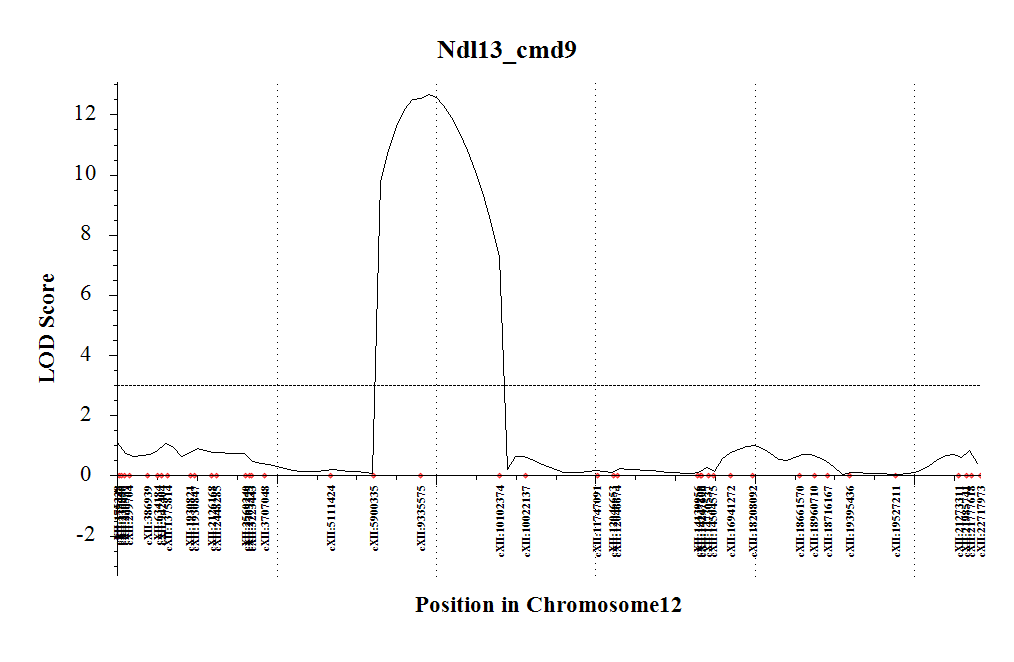


qCMDc1A


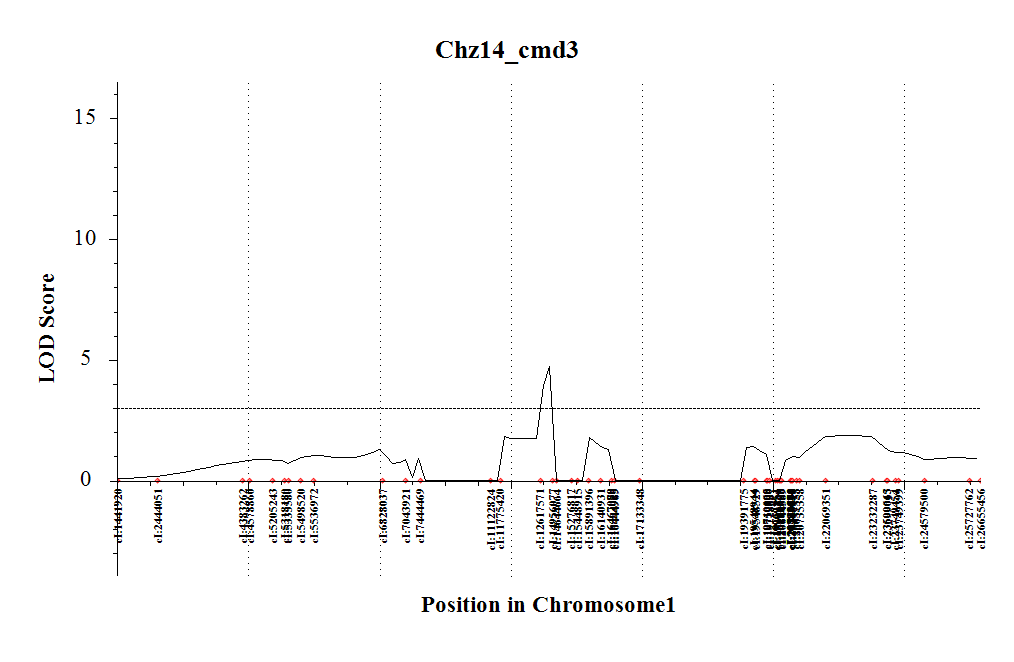


qCMDc2A
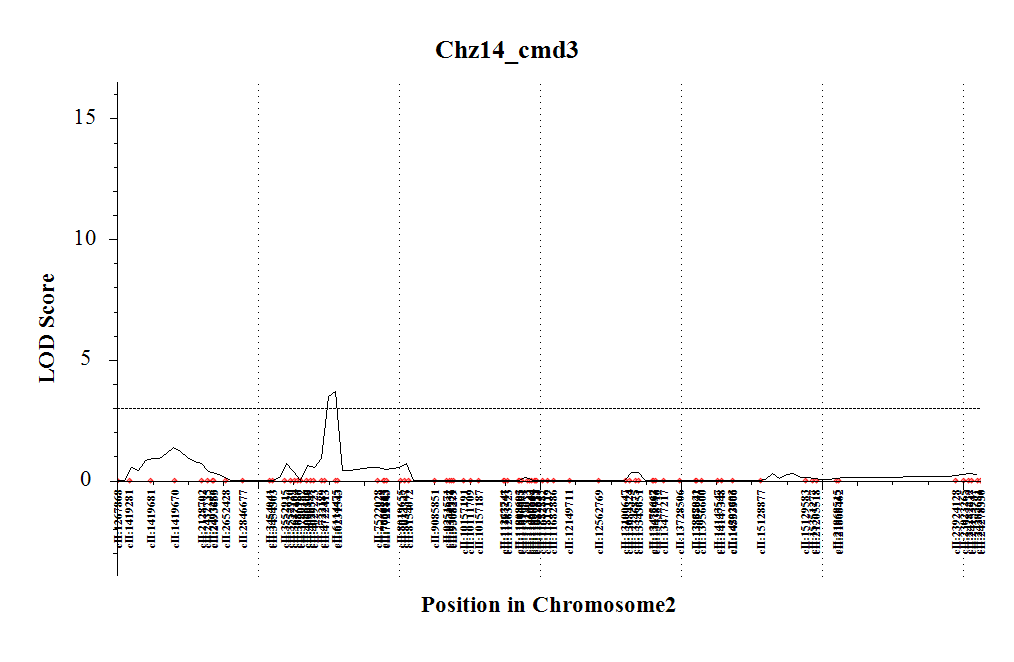


qCMDc12A
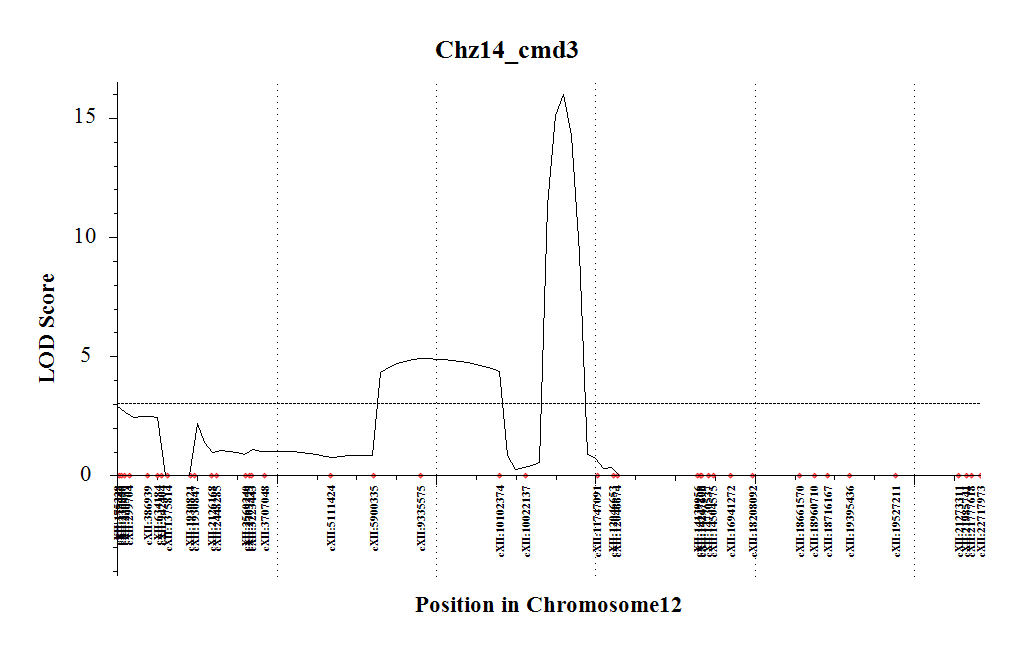


qCMDc3A


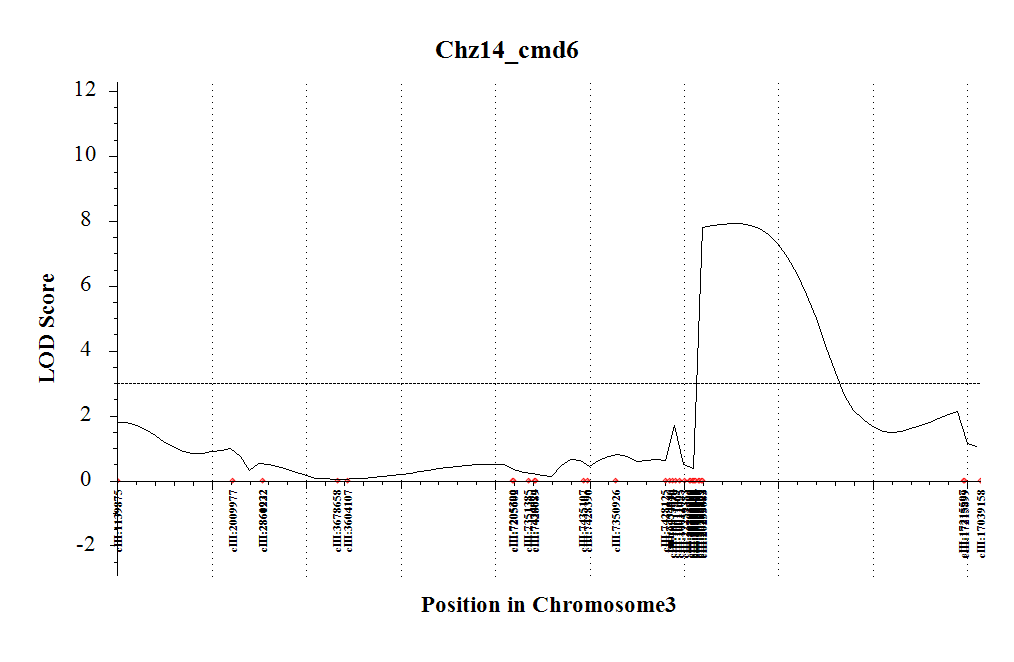


qCMDc12A


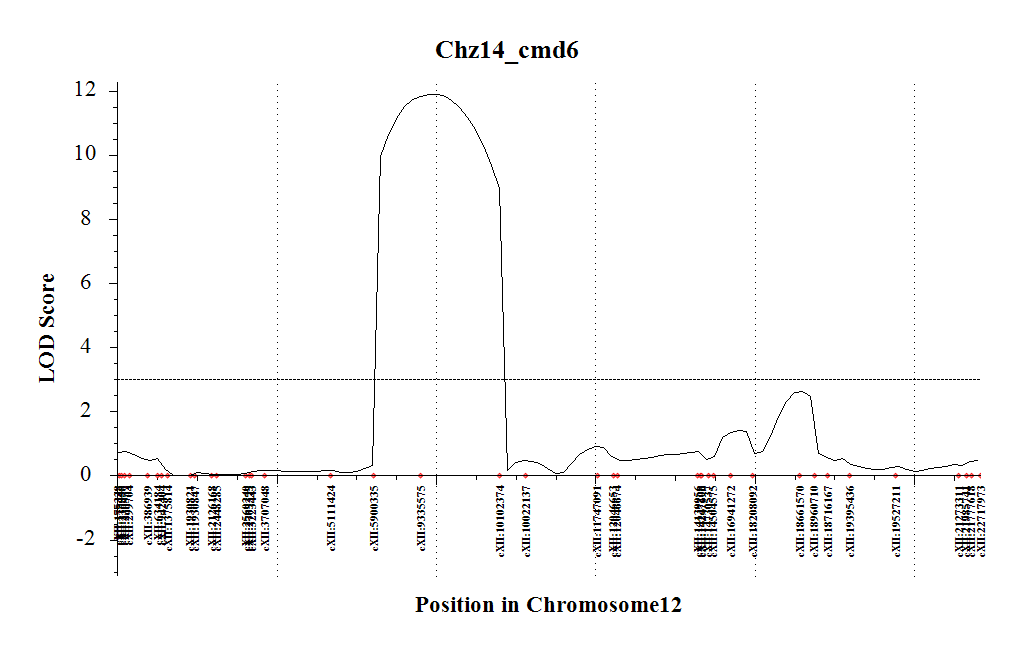


qCMDc16A


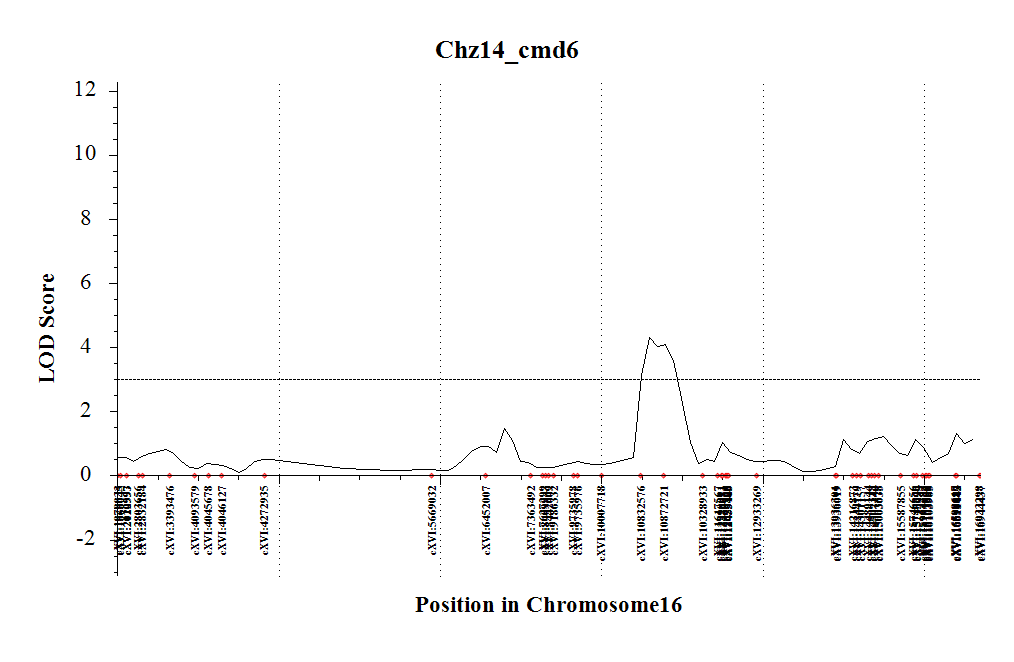


qCMDc1A


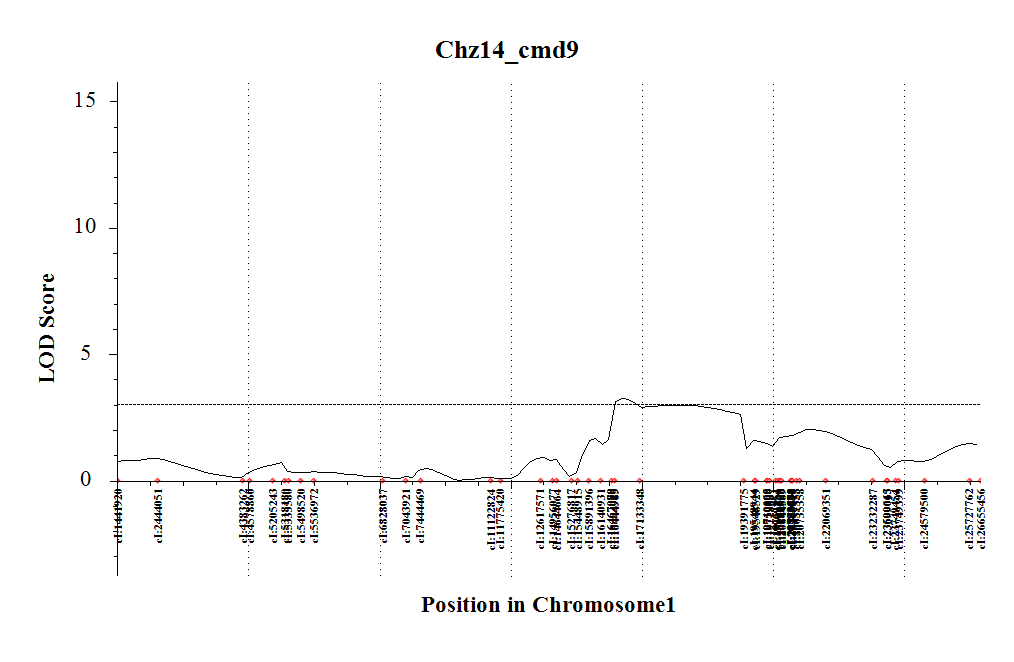


qCMDc3A


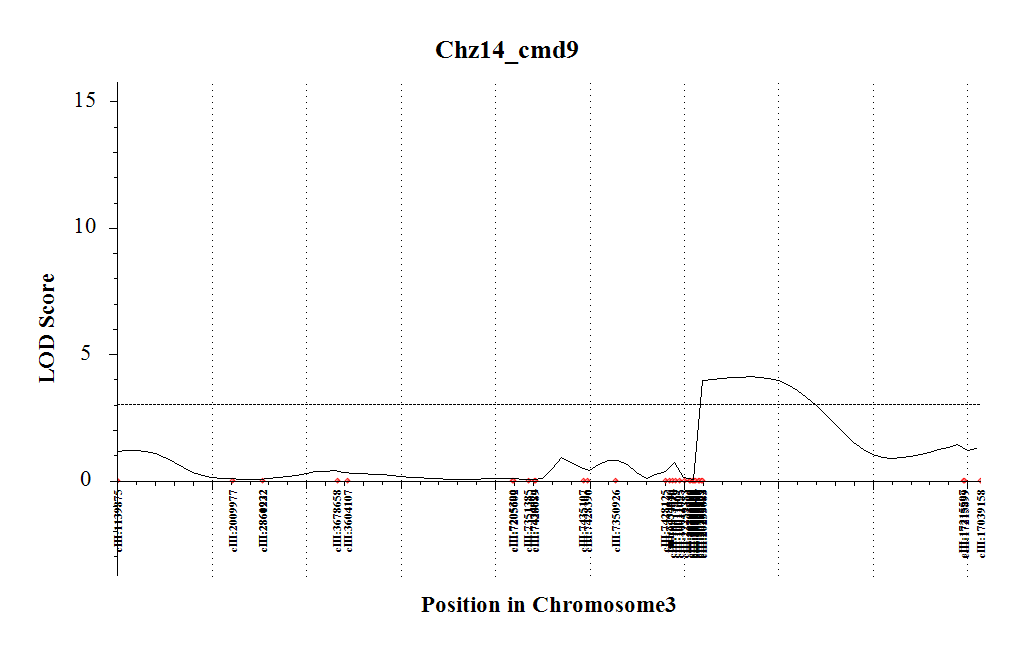


qCMDc5A


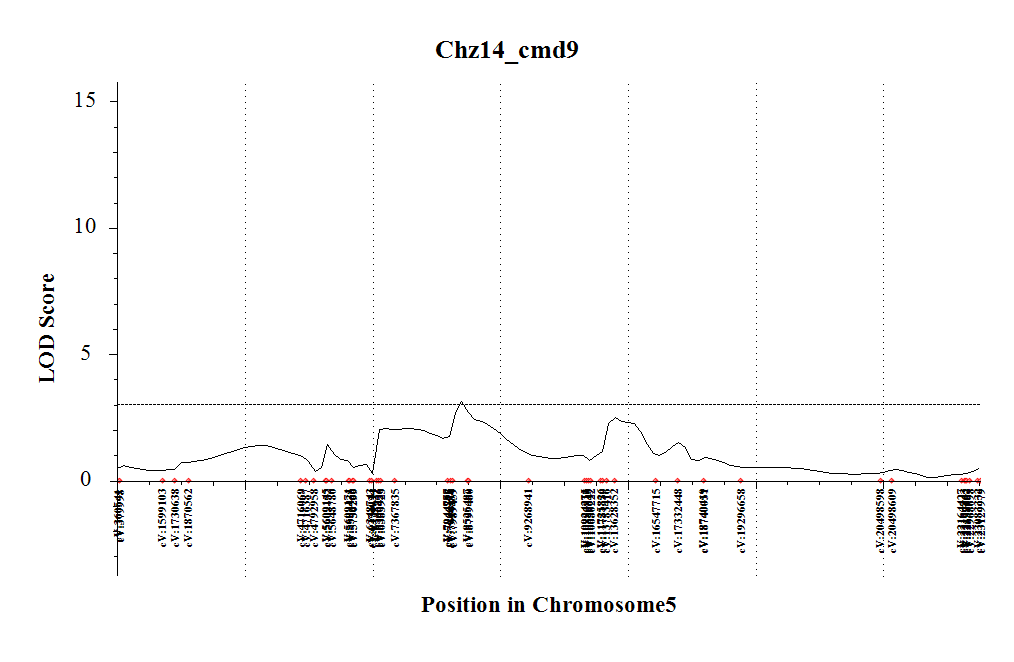


qCMDc6A


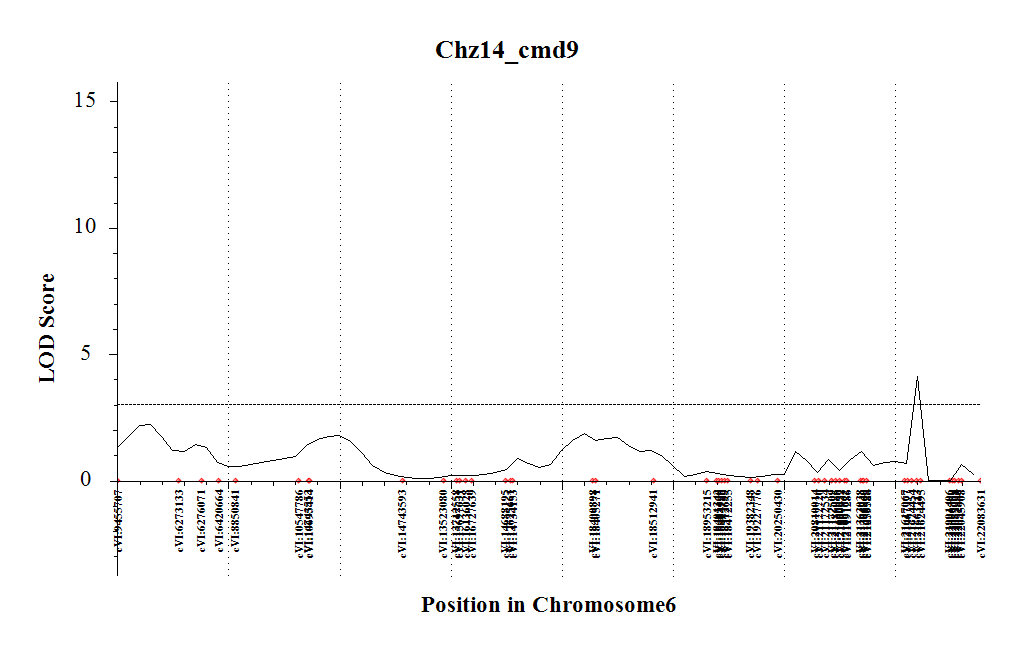


qCMDc12A


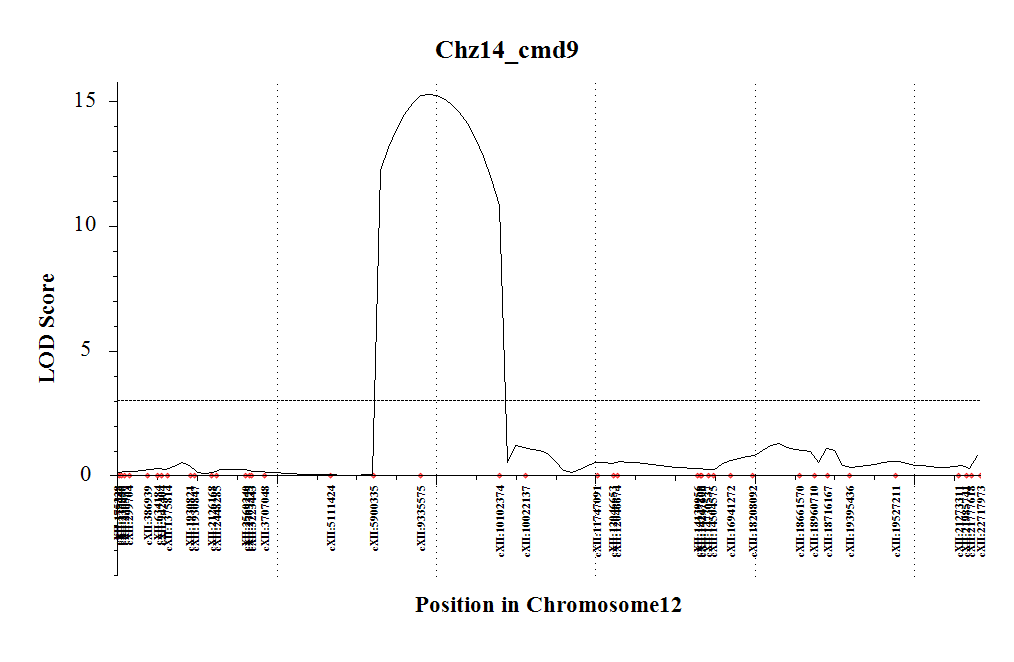


qCMDc2A


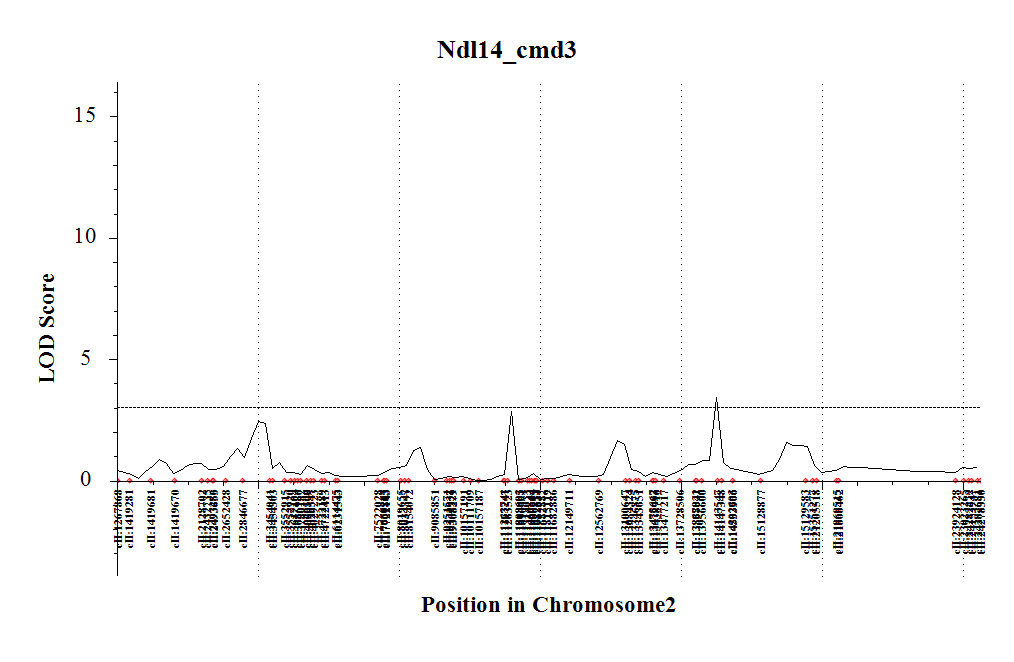


qCMDc5A


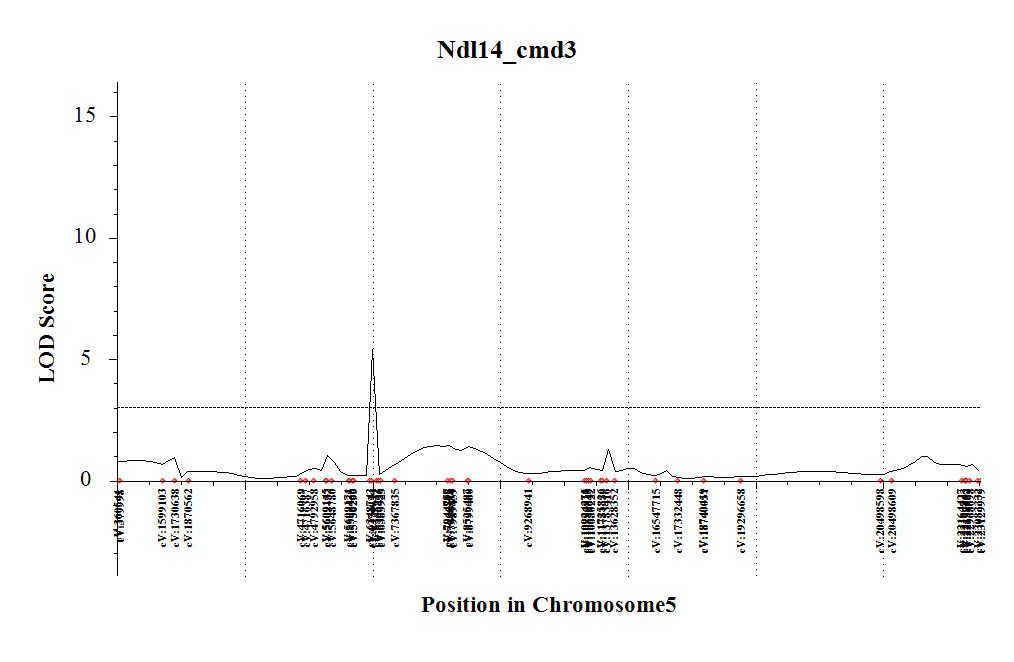


qCMDc6A


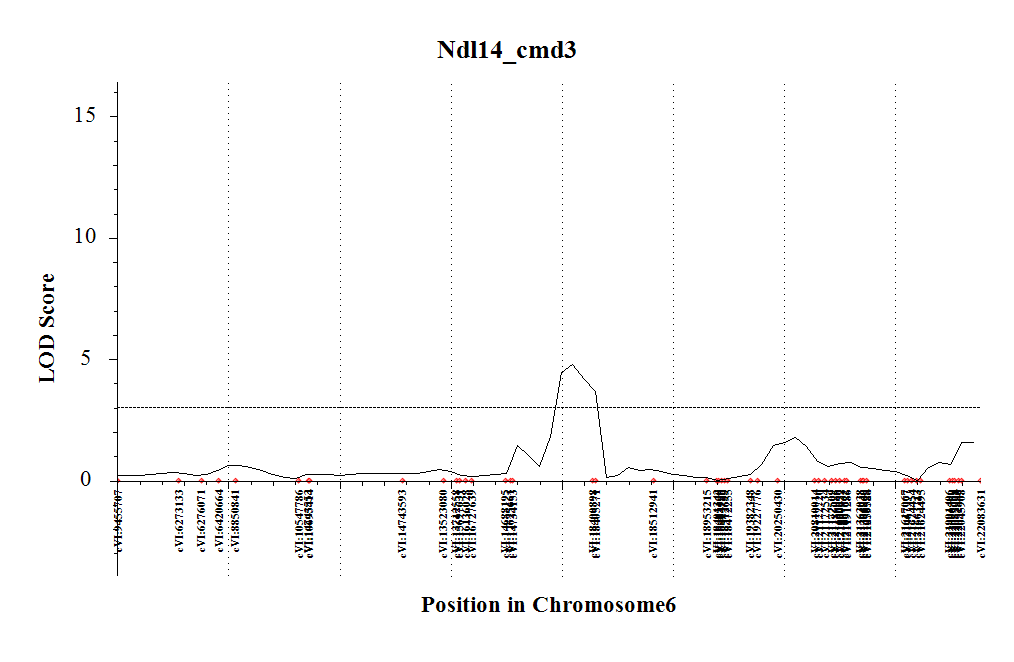


qCMDc9A


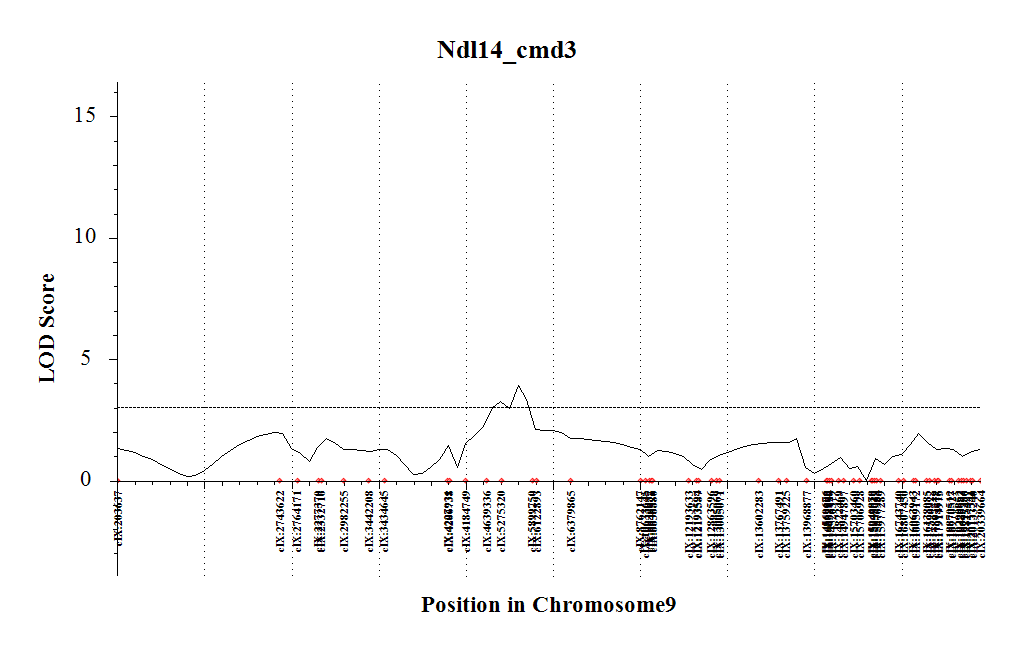


qCMDc10A


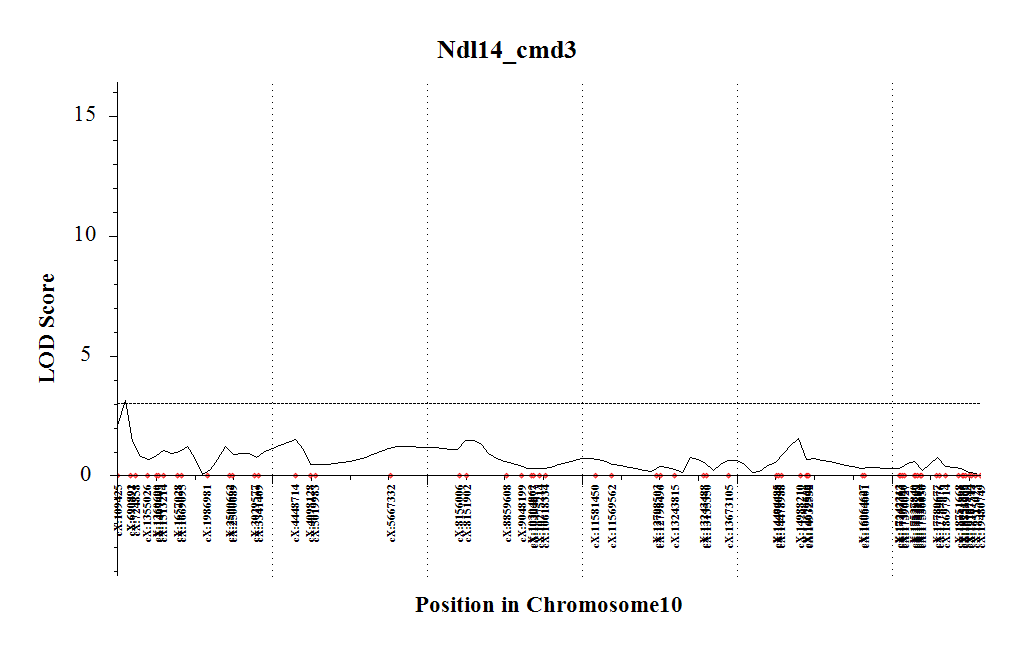


qCMDc12A


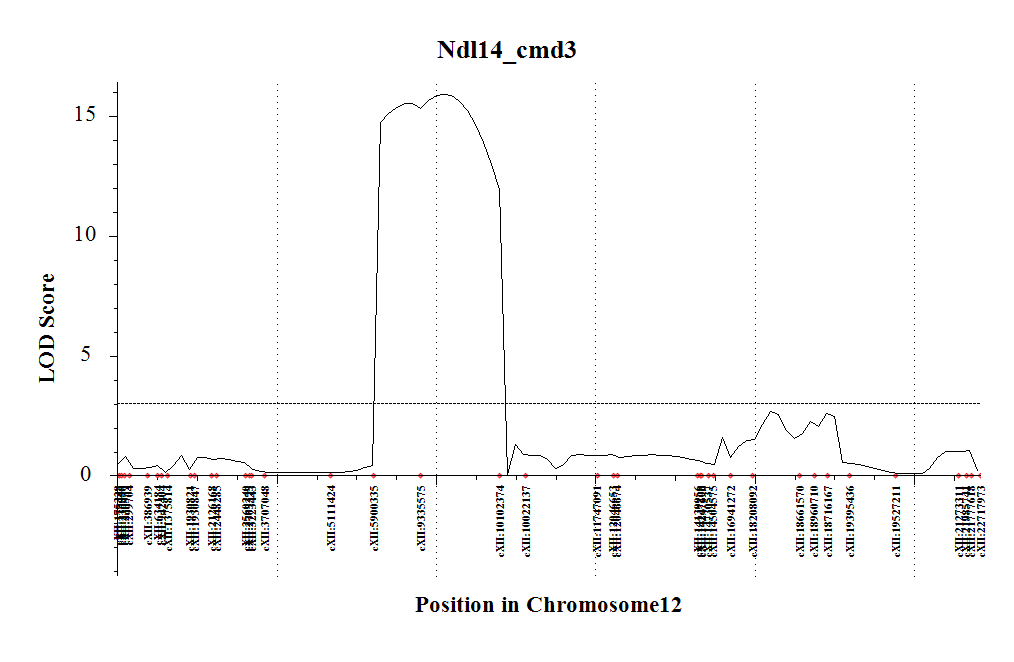


qCMDc15A


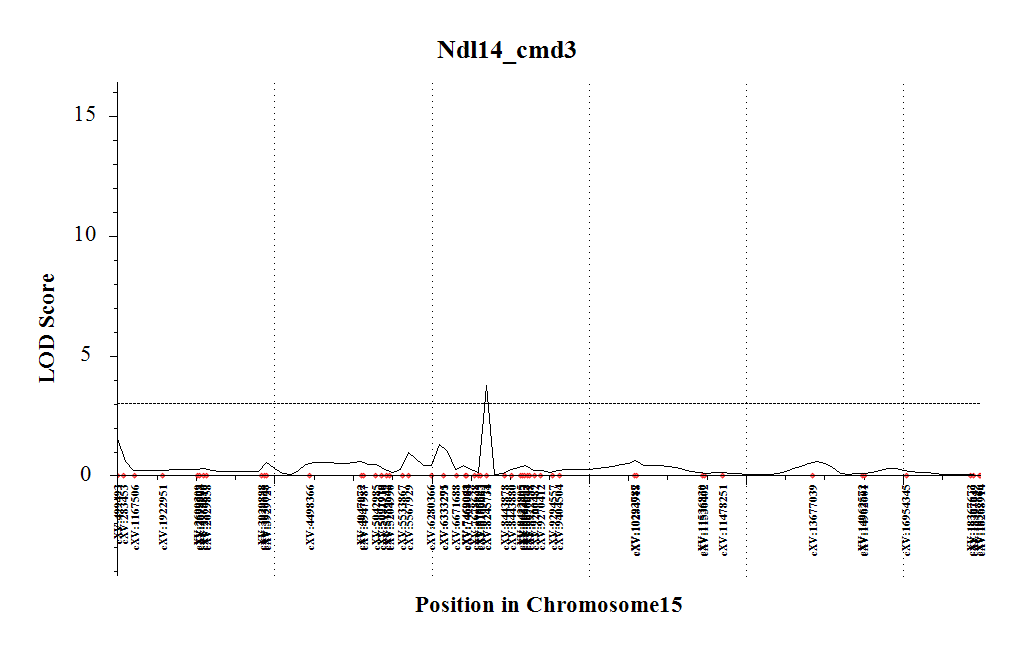


qCMDc6A


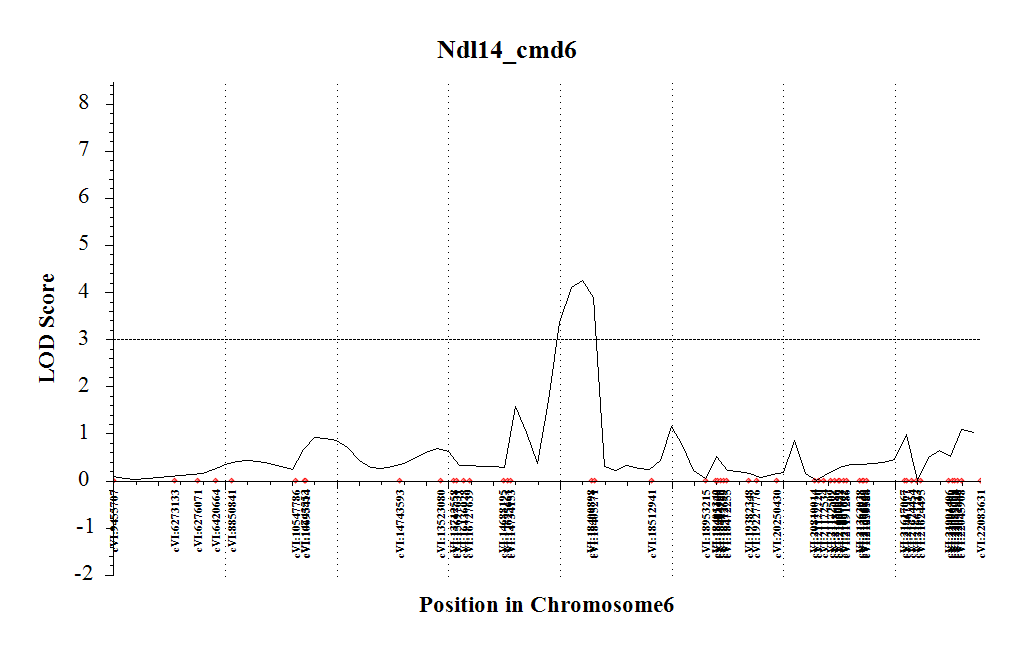


qCMDc8A


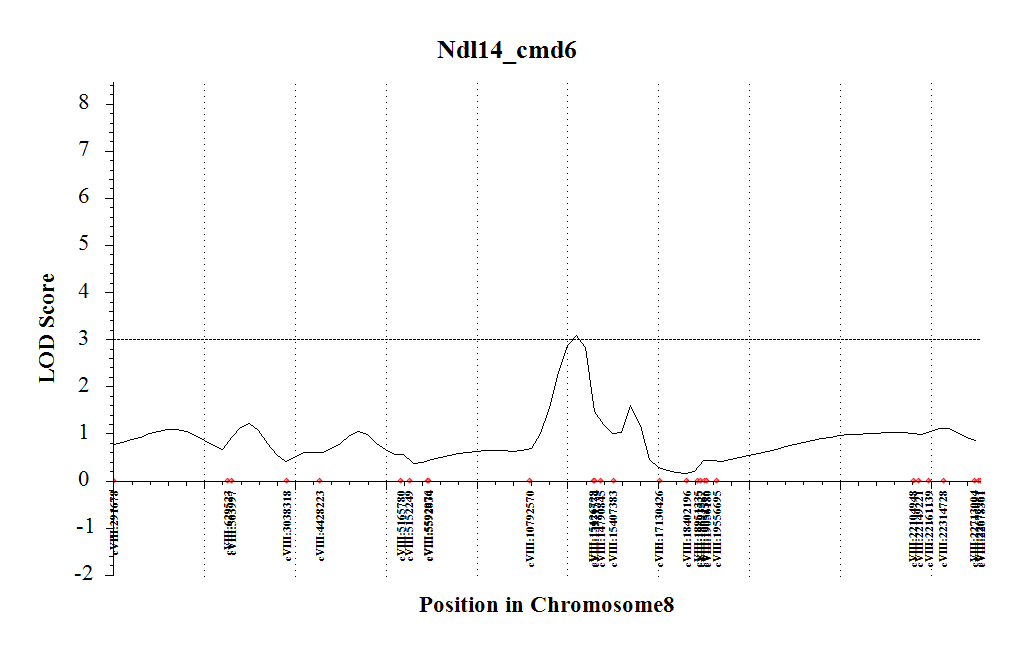


qCMDc10A


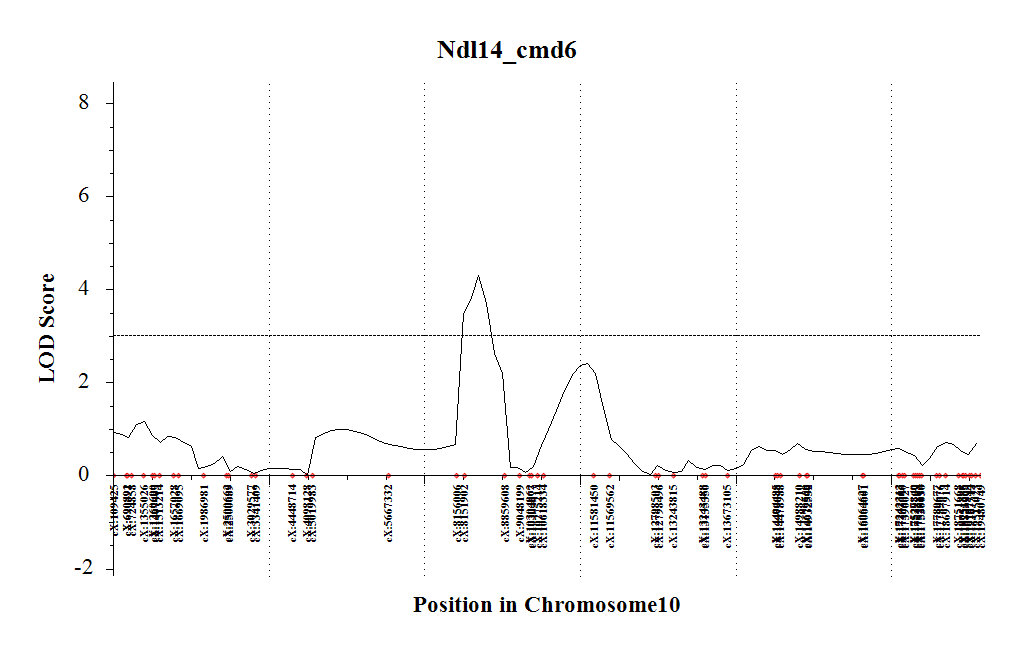


qCMDc12A


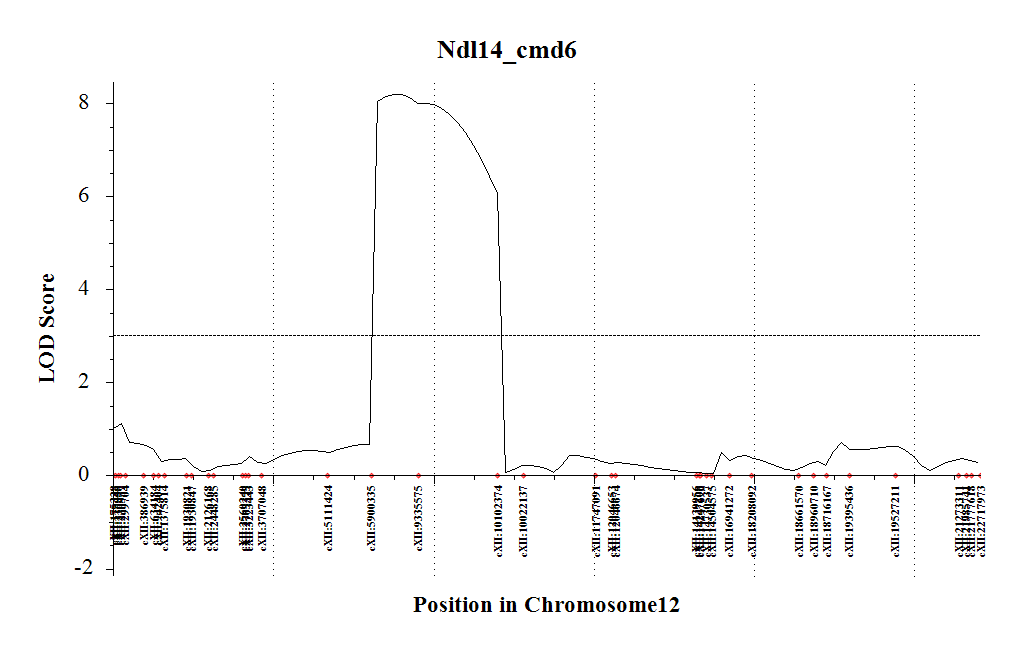


qCMDc1A


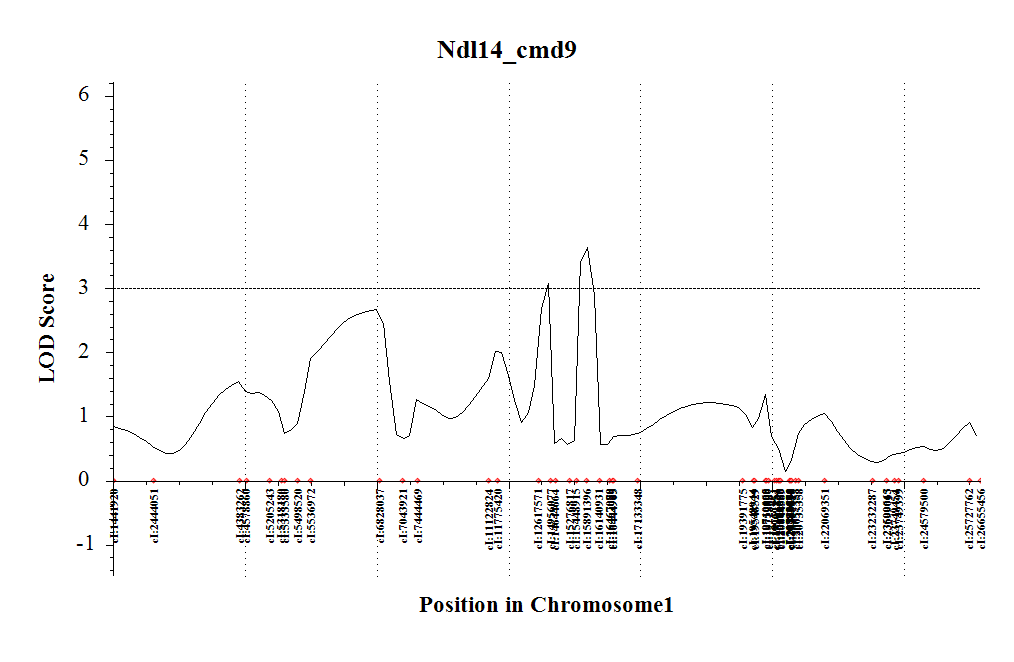


qCMDc10A


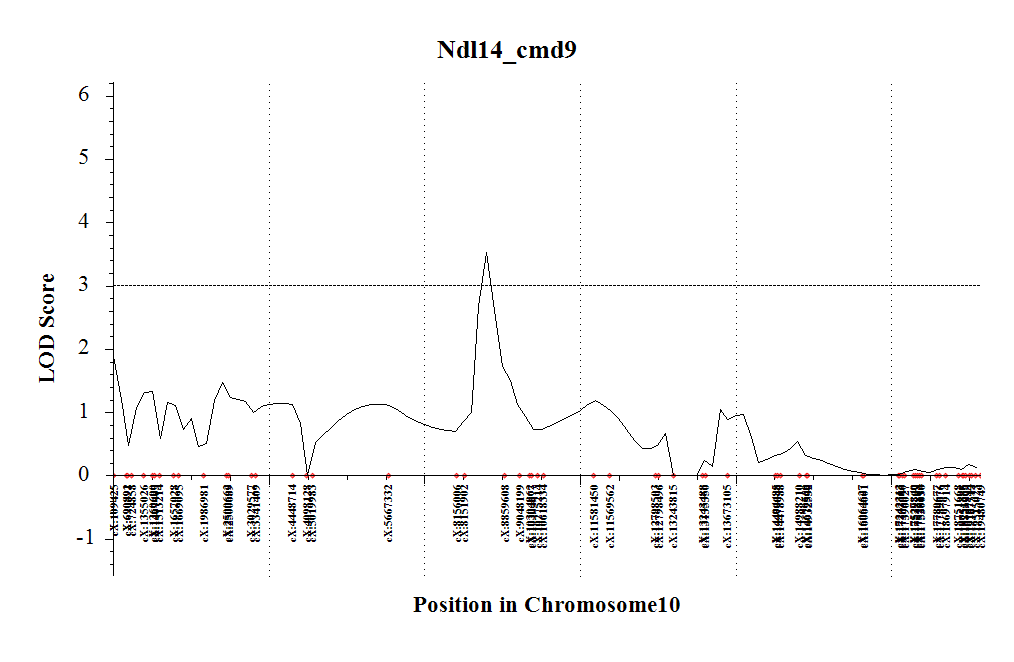


qCMDc12A


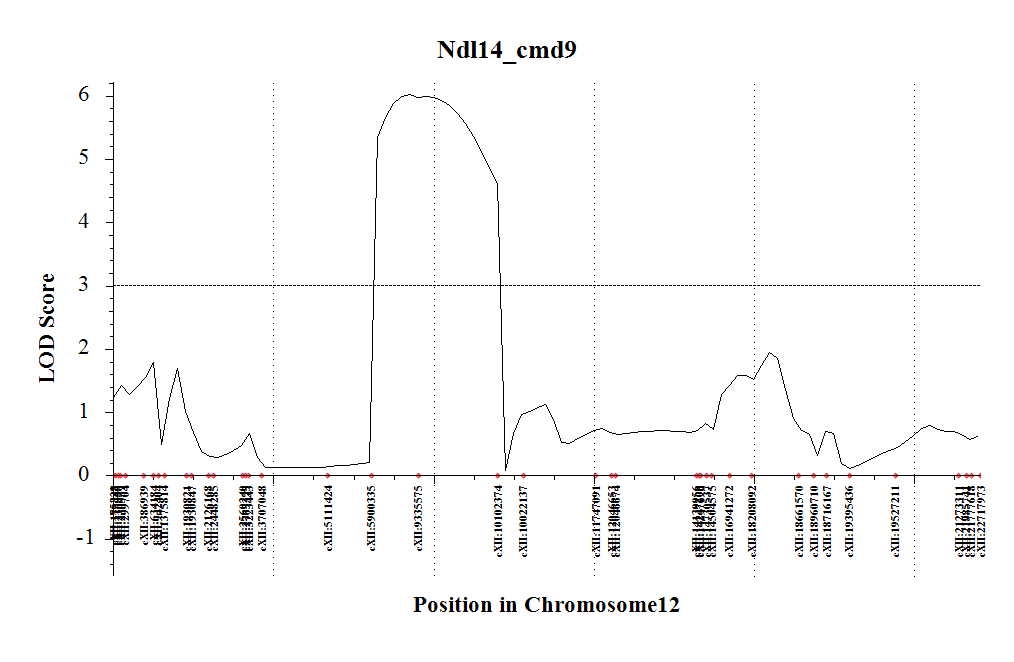


qCMDc13A


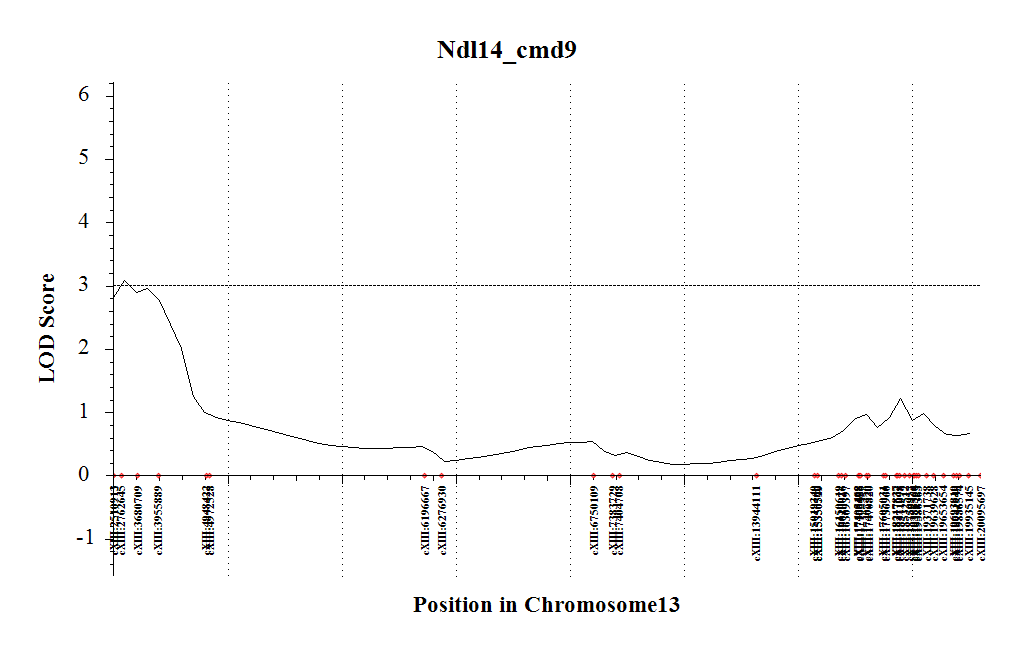

Supplement: Supplementary file 10 — Note 10: GACD LOD Profiles showing the genomic regions in Namikonga-Albert F1 individuals that are associated with CMD resistance (DOCX 1563 kb) [file 122_2017_2943_MOESM10_ESM.docx]
